# A prospective, randomized, non-blinded, non-inferiority pilot study to assess the effect of low-dose anti-thymocyte globulin with low-dose tacrolimus and early steroid withdrawal on clinical outcomes in non-sensitized living-donor kidney recipients

**DOI:** 10.1371/journal.pone.0280924

**Published:** 2023-03-01

**Authors:** Youngmin Ko, Yu-Mee Wee, Sung Shin, Mi Joung Kim, Monica Young Choi, Dong Hyun Kim, Seong Jun Lim, Joo Hee Jung, Hyunwook Kwon, Young Hoon Kim, Duck Jong Han

**Affiliations:** 1 Department of Surgery, Division of Kidney and Pancreas Transplantation, Asan Medical Center, University of Ulsan College of Medicine, Seoul, Korea; 2 Department of Medical Science, Asan Medical Institute of Convergence Science and Technology, Asan Medical Center, University of Ulsan College of Medicine, Seoul, Korea; Weill Cornell Medicine, UNITED STATES

## Abstract

**Background:**

The optimal dose of anti-thymocyte globulin (ATG) as an induction regimen in Asian living-donor kidney recipients is unclear.

**Methods:**

This is a pilot study in which 36 consecutive patients undergoing living-donor kidney transplantation were randomly assigned to receive either 4.5 mg/kg (n = 19) or 6.0 mg/kg (n = 17) of ATG; all patients had corticosteroid withdrawal within 7 days. The primary end point was a composite of biopsy-proven acute rejection, *de novo* donor-specific antibody formation, and graft failure.

**Results:**

At 12 months post-transplant, biopsy-proven acute rejection was more common in the ATG4.5 group (21.1%) than in the ATG6.0 group (0%)(*P* = .048). Importantly, the rate of the composite end point was significantly higher in the ATG4.5 group (36.8% vs 0%)(*P* = .006). There were significant differences in neither the renal function nor adverse events between the two groups. One case of death-censored graft failure occurred in the ATG4.5 group and no mortality was observed overall. Compared with pre-transplantation, T cells, natural killer (NK) cells, and natural killer T (NKT) cells were significantly decreased in the first week post-transplantation except for B cells. Although T and NKT cells in both groups and NK cells in the ATG4.5 group had recovered to the pre-transplant levels, NK cells in the ATG6.0 group remained suppressed until six months post-transplant.

**Conclusions:**

Compared with ATG 6.0 mg/kg, ATG 4.5 mg/kg with early corticosteroid withdrawal and low dose maintenance regimen was associated with higher rates of acute rejection in non-sensitized Asian living-donor kidney recipients.

**Trial registration:**

ClinicalTrials.gov: NCT02447822.

## Introduction

In recent decades, rabbit anti-thymocyte globulin (ATG) was the most commonly used induction agent for kidney transplantation worldwide. ATG was found to induce T-cell depletion and modulate cell surface molecules and adhesion molecules that regulate T-cell function and leukocyte-endothelial interaction, respectively [1–4]. Previous studies demonstrated that ATG is more effective than anti-IL2 receptors such as basiliximab and daclizumab in reducing the incidence and severity of acute rejection [5,6]. Although ATG is effective in preventing acute rejections, it can cause more complications such as infection and malignancy [7–10]. Therefore, it is important to set the minimum effective dose of ATG with early steroid withdrawal.

Several studies have reported the dose optimization of ATG as induction therapy in kidney transplantation [1,11–14]. In those studies, the minimal doses of ATG varied and ranged from 1.5 mg/kg to 7.5 mg/kg according to the respective protocols. However, most of the studies were performed in Western countries and only few have focused on determining the optimal dose of ATG in Asian kidney recipients. It has long been recognized that immunosuppressant pharmacokinetics exhibit ethnicity-specific differences in bioavailability and dose-dependent systemic exposure. Recently, several studies reported a higher incidence of infectious complications in Asian kidney recipients with ATG induction which is the main reason why a physician is reluctant to use ATG as induction regimen [15–17]. Therefore, it is necessary to understand clinical outcomes and adverse events according to the dose of ATG in Asian patients. In addition, it is important to understand which subsets of lymphocytes are depleted after ATG administration and whether they are recovered during follow-up.

The aim of this study was to compare the efficacies of 4.5 mg/kg ATG and 6.0 mg/kg ATG in non-sensitized living-donor kidney recipients with early steroid withdrawal in an Asian population, and to investigate the immunologic profiles thereof during follow-up.

## Research design and methods

### Study design, patient selection, and randomization

The study design was an a prospective, open-label, randomized, non-blinded, non-inferiority pilot study in which 36 consecutive adult patients undergoing living-donor kidney transplantation were randomly assigned to receive either 4.5 mg/kg (n = 19) or 6.0 mg/kg (n = 17) of ATG at Asan Medical Center (Seoul, South Korea); the enrollment of patients was initiated in January 2016. The data safety monitoring board at our center ceased the trial in September 2017, which was due to the more frequently observed composite events of biopsy-proven acute rejection (BPAR) and *de novo* donor-specific antibody formation during 12 months follow-up after kidney transplantation in the ATG4.5 group. After enrollment at kidney transplantation, each patient was followed up until at least twelve months post-transplant. Inclusion criteria were adult patients who undergo living donor kidney transplantation. Patients were excluded if they were a multi-organ transplant recipient, had a panel-reactive antibody of more than 20% or pre-transplant donor-specific antibody, prepared for ABO- or HLA-incompatible kidney transplantation, had a kidney allograft from an HLA-identical donor, had re-transplantation, or had a known contraindication to the administration of ATG. All recipients had a kidney allograft from a living donor at Asan Medical Center. The study was approved by the institutional review board of Asan Medical Center (approval number: 2014–1213) and written informed consent was achieved from all recipients and donors one day before transplantation or donation. The study is listed on http://clinicaltrials.gov (NCT02447822) and was performed under full compliance with the principles of the Declaration of Helsinki. None of the transplant donors was from a vulnerable population and all donors or next of kin provided written informed consent that was freely given. Neither medical costs were covered nor other cash payments were provided to the family of the donor.

All patients were randomly assigned in a computer-generated 1:1 variable-block randomization performed by a study coordinator at our center to receive ATG (Thymoglobuline^®^, Sanofi Genzyme) of either 1.5 mg/kg/day × 3 doses (4.5 mg/kg total dose) or 1.5 mg/kg/day × 4 doses (6.0 mg/kg total dose) as an induction therapy.

### Induction and maintenance immunosuppression and prophylaxis against infection

Before the administration of ATG (1.5 mg/kg/day intravenously), chlorpheniramine and acetaminophen were given intravenously as a premedication. The dose of ATG was reduced by 50% in patients with thrombocytopenia (platelet count 50,000–75,000 per cubic millimeter) or neutropenia (absolute neutrophil count 2000–3000 per cubic millimeter). ATG was discontinued when the patiet developed severe thrombocytopenia (platelet count < 50,000 per cubic millimeter) or severe neutropenia (absolute neutrophil count < 2000 per cubic millimeter).

The maintenance immunosuppressants consisted of tacrolimus, mycophenolate mofetil, and seven-day methylprednisolone taper. Tacrolimus was initiated two days before kidney transplantation at a dose of 0.05 mg/kg twice a day, and the target trough level was 6–8ng/ml until one year post-transplant. Mycophenolate mofetil was given 750 mg twice a day in both groups. Methylprednisolone was administered at a dose of 500 mg intravenously on day 0, 250 mg on day 1, and 125 mg on day 2 and 3. Thereafter, a fast taper was carried out with oral prednisone in the first week post-transplant.

All recipients received oral doses of trimethoprim 80 mg-sulfamethoxazole 400 mg daily for six months for bacterial and *Pneumocystis jiroveci* prophylaxis. Valganciclovir was administered for cytomegalovirus (CMV) prophylaxis for six months when a seronegative recipient had kidney transplantation from a seropositive donor. For low-to-intermediate risk patients, CMV monitoring was performed on a weekly basis using CMV-PCR assay for preemptive treatment. If the viral load of CMV was more than 4.0 log in the CMV-PCR assay, intravenous ganciclovir or oral valganciclovir was administered until CMV viremia was eliminated. In addition, BKV-PCR assay was also done on a monthly basis for BKV monitoring.

### HLA antibody testing and HLA typing

HLA antibody testing and HLA typing were performed before transplantation. Specificities of antibodies were reported by LABScreen® Single Antigen Class I and Class II assay (One Lambda Inc., Canoga Park, CA). Single antigen beads were used to test for antibodies against HLA-A, -B, -C, -DRB1, -DRB3, -4 and -5, and -DQB1. Using BioSewoom™, typing of low-to-medium resolution HLA-A, -B, -C and DR was done. PCR/SSP kit (BioSewoom Inc., Seoul, Korea) and high resolution HLA-DQB1 typing was performed by AVITA™ plus HLA-DQB1 SBT kits (BioWithus Inc., Seoul, Korea).

After transplantation, LABScreen® Single Antigen Class I and Class II assay was performed every one or two months and when there was an acute deterioration of renal function.

### Peripheral blood mononuclear cells (PBMC) isolation and flow cytometry and data analysis

We acquired blood samples from the recipients at two days before transplantation, one-week post-transplant, and one, three, and six months post-transplant. PBMCs were separated by density-gradient centrifugation using lymphocyte isolation sterile solution Ficoll-Paque^TM^ PLUS (GE Healthcare, Sweden). PBMCs were collected from the cell fraction in the interface between blood and ficoll, and washed with RPMI-1640 10% FBS medium and re-suspended in freezing media (heat-inactivated FBS with 10% dimethyl sulfoxide). A total of 1 × 10^5^ PBMCs were incubated in 30 minutes with three different antibody mixtures in each flow cytometry analysis set, and the stained cells were fixated with 6% of paraformaldehyde.

In flow cytometry analysis, subsets of natural killer (NK) cells were stained with the following antibodies: PE-conjugated anti-CD56 antibody (5.1H11, IgG1κ, BioLegend), FITC-conjugated anti-CD3 antibody (UCHT1, IgG1κ, BioLegend), PerCP/Cy5.5-conjugated anti-CD57 antibody (HNK1, IgM, BioLegend), PE/Cy7-conjugated anti-NKG2D (CD159c) antibody (1D11, IgG1κ, BioLegend), APC-conjugated anti-NKG2A (CD159a) antibody (REA110, IgG1κ, Miltenyi Biotec), and APC/Cy7-conjugated anti-CD16 antibody (B73.1, IgG1κ, BioLegend). Lymphocytes that were CD3^-^CD56^+^ and positive for at leat one of the other surface markers (CD57, NKG2D, NKG2A, and CD16) were considered as NK cells. T cells were defined as CD3^+^CD56^-^ lymphocytes whereas CD3^+^CD56^+^ lymphocytes were designated as natural killer T (NKT) cells. For the second set, subsets of B cells were measured with the following antibodies: PE-conjugated anti-CD5 antibody (UCHT2, IgG1κ, BioLegend), FITC-conjugated anti-HLA-DR antibody (L243, IgG2aκ, BioLegend), PerCP/Cy5.5-conjugated anti-CD27 antibody (O323, IgG1κ, BioLegend), APC-conjugated anti-CD1d antibody (51.1, IgG2bκ, BioLegend), and APC/Cy7-conjugate anti-CD19 antibody (HIB19, IgG1κ, BioLegend). All the CD3^-^CD19^+^ lymphocytes were defined as B cells. The last set was prepared to analyze the correlation between NK cells and B cells in lymphocytes and B cell populations in monocytes by using PE-conjugated anti-CD56 antibody (5.1H11, IgG1κ, BioLegend), FITC-conjugated anti-CD3 antibody (UCHT1, IgG1κ, BioLegend), PerCP/Cy5.5-conjugated anti-CD27 antibody (O323, IgG1κ, BioLegend), PE/Cy7-conjugated anti-CD11b antibody (ICRF44, IgG1κ, BioLegend), APC-conjugated anti-CD25 antibody (BC96, IgG1κ, BioLegend), and APC/Cy7-conjugated anti-CD16 antibody (B73.1, IgG1κ, BioLegend). PBMCs were prepared to measure the expression of immune cells by using Becton Dickinson FACSCanto II Flow Cytometry (BD Biosciences). Compensation controls were single-stained PBMCs for each antibody. Isotype control antibodies were added to compensation control samples to determine the unspecific antibody-binding to cell membranes. Spillover from each fluorechrome was mathematically removed. Negative/positive population was gated in consideration of data spreading. The acquired raw data were analyzed by FlowJo version 10.0 software and exported to Excel spreadsheets [18,19].

### End points

The primary efficacy end point was a composite of BPAR, *de novo* donor-specific antibody formation, and graft failure at 12 months after kidney transplantation. BPAR was determined by pathological evidence interpreted by the Banff criteria [20–23]. Delayed graft function was defined as the need for dialysis during the first week after transplantation, excluding a single session for the treatment of hyperkalemia. Kidney graft failure was defined as the need for a transplant nephrectomy, retransplant, or recommencement of dialysis. The secondary efficacy end point was renal function determined by eGFR (CKD-EPI) at one and six months post-transplant and one and two years post-transplant. The safety end points included infection, leukopenia, thrombocytopenia, and malignancy. Leukopenia was defined as a white cell count of less than 2500 per cubic millimeter. Thrombocytopenia was defined as a platelet count of less than 80,000 per cubic millimeter.

### Statistical analysis and data availability

Mann-Whitney U test was used to compare continuous variables between the two groups. Categorical variables were compared using the chi-squared test. Survival rates related to the composite outcomes were calculated using the Kaplan-Meier method and compared using the log-rank test. A linear mixed model was applied to analyze the difference in the pattern of time-dependent change according to the dose of ATG. Time was entered as a categorical fixed-effects variable and the patient identity as a random effect. We first analysed the effect of time on expression of each immune cell separately. Thereafter we constructed a model to look at the effect of the dose of ATG on expression of each immune cell, taking time into account. All covariates for models were chosen *a priori*. Optimal covariance structures (among Unstructured, Toeplitz, Compound symmetry (CS), and First-order autoregressive (AR(1)) for each outcome were selected through a likelihood ratio test. In the comparison of CS and AR(1), the model with a small AIC was chosen. For the sample size calculation, we assessed comparability in primary efficacy end point between the two groups. The expected background rate of primary efficacy failure for the ATG 6.0 mg/kg group was estimated to be 40% [6]. A sample size of 154 patients (77 in each arm) was estimated to provide 80% power at one-sided 5% significance level to get an evidence of non-inferiority of ATG 4.5 mg/kg versus ATG 6.0 mg/kg for the primary efficacy end point based on a non-inferiority margin of 8%.

For the primary endpoint, it is judged whether the upper limit of the 90% two-sided confidence interval for the difference between the two groups exceeds the non-inferiority margin (10%). In addition, a one-sided Z test for non-inferiority was performed. *P* values < .05 were considered statistically significant. All statistical analyses were performed using SPSS version 21.0 for Windows (SPSS Inc., Chicago, IL, USA). Except for the privacy of individuals that participated in the study, the data is provided within supporting information files.

## Results

### Characteristics of the enrolled patients

**Fig 1:**
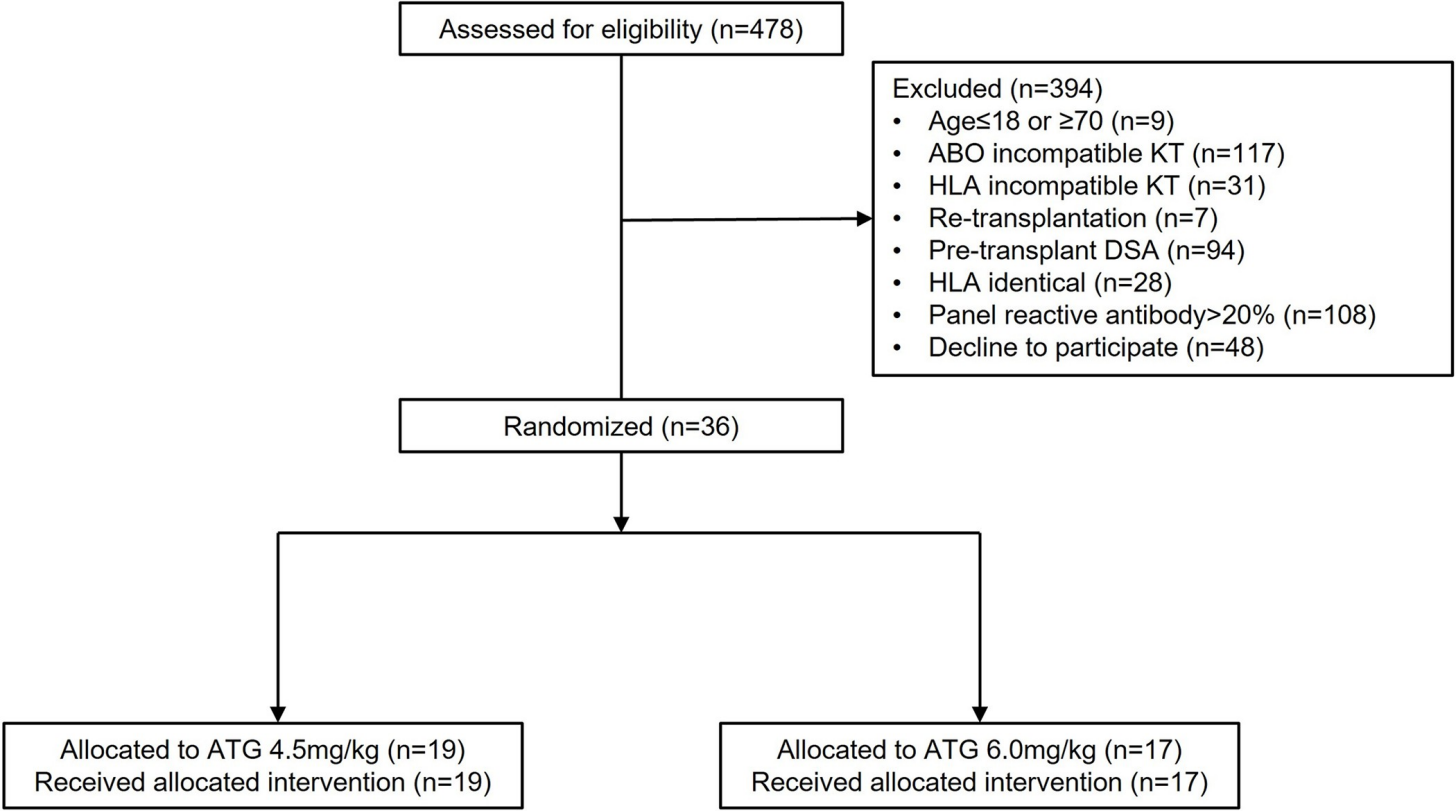
Flow diagram of enrollment, randomization, follow-up, and analysis. ATG, anti-thymocyte globulin; KT, kidney transplantation.

A total of 478 patients were screened for potential enrollment in this study. Among the 478 recipients, 394 were excluded due to age≤18 or ≥70 (n = 9), ABO incompatible KT (n = 117), HLA incompatible KT (n = 31), re-transplantation (n = 7), pre-transplant DSA (n = 94), HLA identical donor (n = 28), panel reactive antibody>20% (n = 108), and declination to participate (n = 48) (Fig 1). After excluding 394 recipients, 84 recipients met criteria, of which 36 patients agreed to participate and were enrolled. After randomization, 19 were assigned to receive 4.5 mg/kg ATG and 17 were assigned to receive 6.0 mg/kg ATG. Follow-up data were collected until February 2020. There were no significant differences in the baseline characteristics of recipients and donors between the two groups including the numbers of ABDR, DR, and DQ mismatches (Table 1).

**Table 1:** Baseline characteristics according to the dosage of antithymocyte globulin.

**Variables** **Recipient** Age, y, median [IQR] Female gender, n (%) Body mass index, kg/m^2^, median [IQR] Hypertension, n (%) Diabetes, n (%) Primary cause of ESRD, n (%) Hypertension Diabetes Glomerulonephritis IgA nephropathy Unknown Others Preemptive transplant, n (%) ABDR mismatch, median [IQR] DR mismatch, median [IQR] DQ mismatch, median [IQR] **Donor** Age, y, mean [range] Female gender, n (%) Body mass index, kg/m^2^, median [IQR] 24 hours creatinine clearance, ml/min, median [IQR] 24 hours protein excretion, mg/day, median [IQR]	**Antithymocyte globulin** **6.0mg** **(N = 17)** 43.0 [36.0–48.0] 10 (58.8) 25.3 [20.2–27.9] 16 (94.1) 3 (17.6) 2 (11.8) 3 (17.6) 2 (11.8) 2 (11.8) 5 (29.4) 3 (17.6) 5 (29.4) 3.0 [2.0–4.0] 1.0 [1.0–2.0] 1.0 [0.5–2.0] 49.0 [44.0–56.5] 9 (52.9) 24.1 [21.7–25.8] 104.2 [92.9–125.8] 75.4 [62.3–98.6]	**Antithymocyte globulin** **4.5mg** **(N = 19)** 49.0 [33.0–57.0] 12 (63.2) 21.8 [19.6–25.6] 17 (89.5) 5 (26.3) 2 (10.5) 4 (21.1) 2 (10.5) 5 (26.3) 5 (26.3) 1 (5.3) 6 (31.6) 3.0 [2.0–5.0] 1.0 [1.0–2.0] 1.0 [1.0–1.0] 49.0 [39.0–58.0] 9 (47.3) 23.9 [21.6–26.5] 109.0 [94.2–129.7] 82.5 [67.0–99.7]	**P-value** 0.186 0.827 0.300 0.827 0.661 0.531- - - - - - 0.612 0.900 0.754 0.827 0.650 0.778 0.925 0.684 0.573

IQR, Interquartile range; ESRD, end-stage renal disease.

### Efficacy end points

**Fig 2:**
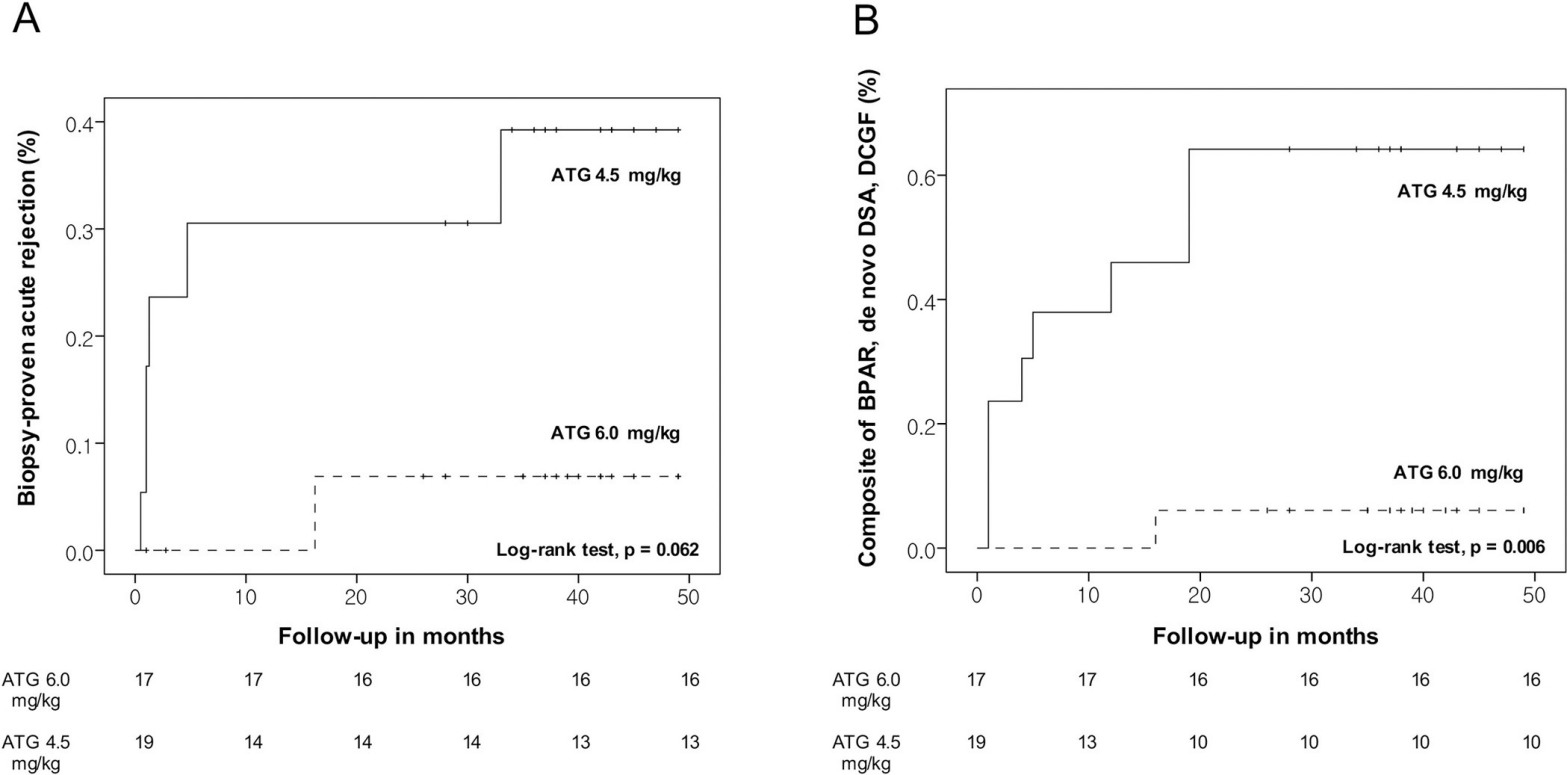
Efficacy end points. Kaplan-Meier curves for BPAR (A) and a composite end point of BPAR, *de novo* DSA, and DCGF (B) according to the dosage of ATG. BPAR, biopsy-proven acute rejection; DSA, donor-specific antibody; DCGF, death-censored graft failure; ATG, anti-thymocyte globulin.

At 12 months post-transplant, BPAR was more common in the ATG4.5 group (21.1%) than in the ATG6.0 group (0%)(log-rank test, *P* = .048) (Fig 2A). Importantly, the rate of the composite end point at 12 months post-transplant was significantly higher in the ATG4.5 group (36.8% vs 0%)(log-rank test, *P* = .006) (Fig 2B). There were no cases of delayed graft function occurring during the first week post-transplant. *De novo* donor-specific antibody (DSA) developed in six (31.6%) patients in the ATG4.5 group and in one (5.9%) patient in the ATG6.0 group (log-rank test, *P* = .092). In the ATG4.5 group, six recipients developed de novo DSA of which three are HLA class I antibodies and the other three are HLA class II antibodies. In the ATG6.0 group, on the other hand, only one had de novo DSA which was HLA class II antibody. There was one (5.3%) who had death-censored graft failure of the patients who had experienced an acute rejection in the ATG4.5 group and none in the ATG6.0 group. There were no mortality cases in both groups during the follow-up period. There were no significant differences in the renal function determined by eGFR (CKD-EPI) between the two groups during the follow-up period (S6 File).

### Adverse events

There were no significant differences in adverse events between the two groups (Table 2). Leukopenia was observed in 10 (56%) cases in the ATG4.5 group and in nine (53%) cases in the ATG6.0 group. Thrombocytopenia was only observed in two (12%) cases in the ATG6.0 group. Although there was an intermittent adjustment of the ATG dose due to leukopenia or thrombocytopenia, all the patients had a designated total dose of ATG according to the protocol. CMV infection was less common in the ATG4.5 group (10.5%) than in the ATG6.0 group (35.3%). BK viremia was only noted in one (5.3%) patient in the ATG4.5 group. The incidence of bacterial infection requiring hospital admission was 15.8% in the ATG4.5 group and 17.6% in the ATG6.0 group. There were no cases of malignancy in both groups during the follow-up period.

**Table 2:** Adverse outcomes according to the dosage of anti-thymocyte globulin.

**Adverse Event** Leukopenia, n (%) Thrombocytopenia, n (%) Infection Cytomegalovirus, n (%) Polyomavirus, n (%) Bacterial infections, n (%) Malignancy, n (%)	**Anti-thymocyte globulin** **6.0mg** **(N = 17)** 9 (52.9) 2 (11.8) 6 (35.3) 0 3 (17.6) 0	**Anti-thymocyte globulin** **4.5mg** **(N = 19)** 10 (52.6) 0 2 (10.5) 1 (5.3) 3 (15.8) 0	**P-value** 1.000 0.216 0.114 1.000 0.925 1.000

### Histopathologic characteristics

During the follow-up period, six patients in the ATG4.5 group and three patients in the ATG6.0 group underwent a for-cause biopsy (Table 3). Among the six patients in the ATG4.5 group, five patients had TCMR only and one patient had both TCMR and ABMR. Among the three patients in the ATG6.0 group, only one patient had pathologic features compatible with acute rejection. There were no significant differences between the two groups in terms of positive C4d staining, interstitial fibrosis and tubular atrophy, and Banff scores.

**Table 3:** Comparison of histopathologic characteristics according to the dosage of anti-thymocyte globulin.

**Variables**	**Anti-thymocyte globulin** **6.0mg/kg** **(n = 3)**	**Anti-thymocyte globulin** **4.5mg/kg** **(n = 6)**	***P* value**
Histopathology, n (%) TCMR only TCMR + ABMR No rejection Positive C4d staining, n (%) IFTA, n (%) Minimal Mild Moderate-to-severe Mean Banff score, mean (SD) g cg mm i ci t ct v cv ah ptc ti	0 1 (33.3) 2 (66.7) 1 (33.3) 2 (66.7) 0 1 (33.3) 0.67 (0.58) 0 0 1.33 (1.53) 1.00 (1.00) 1.00 (1.73) 1.33 (0.58) 0 0.33 (0.58) 0 0.67 (1.15) 1.67 (1.15)	5 (83.3) 1 (16.7) 0 2 (33.3) 2 (33.3) 4 (66.7) 0 0.67 (1.21) 0.33 (0.82) 0 1.83 (1.17) 0.50 (0.55) 1.67 (0.82) 1.00 (0) 0 0.67 (0.52) 0.33 (0.52) 1.33 (1.51) 2.00 (0.89)	0.014 1.000 1.000 0.714 0.714 1.000 0.714 0.548 0.381 0.548 1.000 0.548 0.548 0.548 0.714

NR, no rejection; TCMR, T-cell-mediated rejection; ABMR, antibody-mediated rejection; IFTA, interstitial fibrosis and tubular atrophy; SD, standard deviation.

### Comparison of reconstitution of peripheral lymphocytes and monocytes

**Fig 3:**
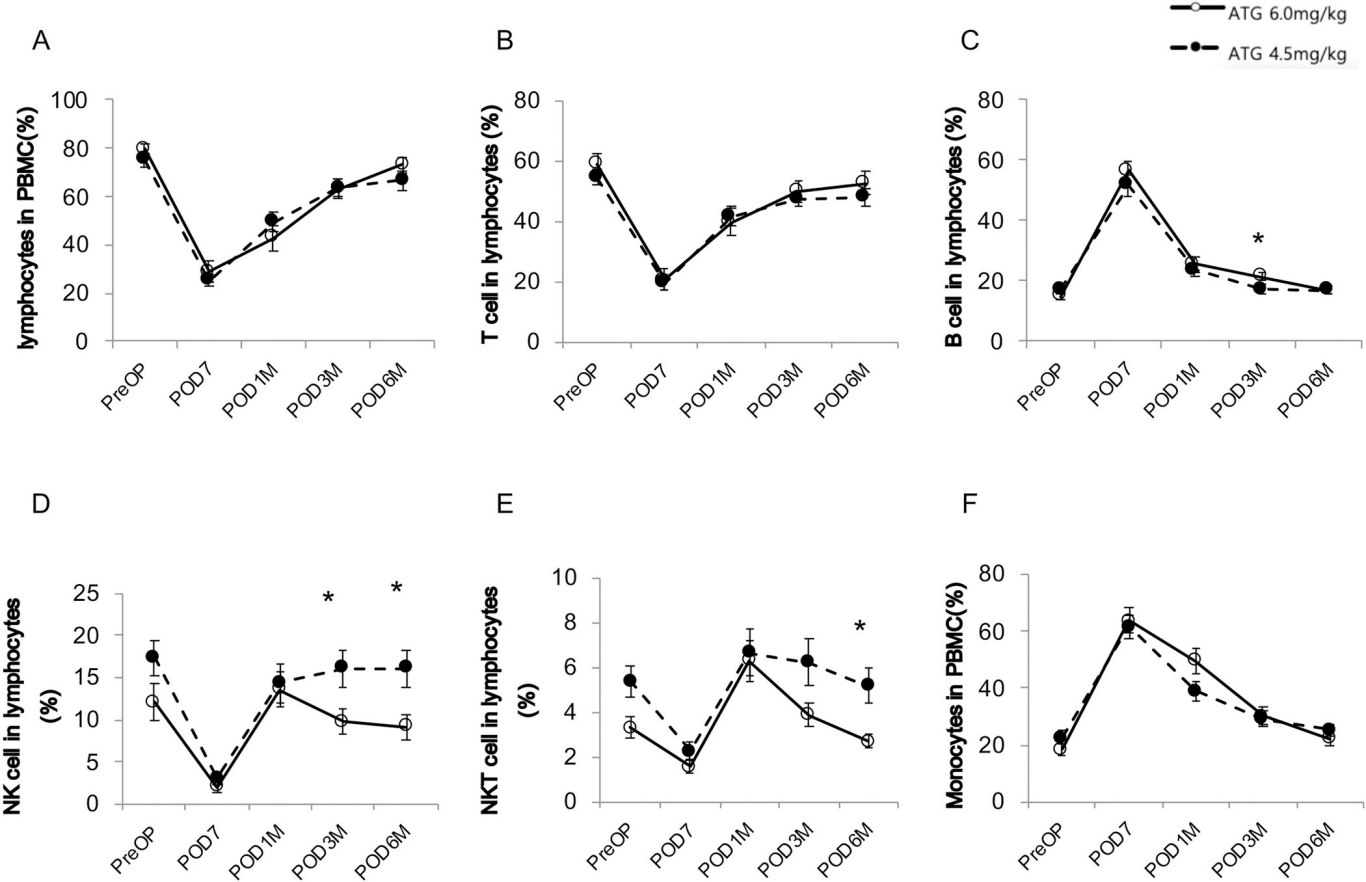
Changes in the proportions of immune cell populations. Total lymphocytes (A), T cells (B), B cells (C), NK cells (D), NKT cells (E), and monocytes (F) during follow-up after kidney transplantation are shown according to the dosage of ATG.

At pre-transplantation, the two groups did not show significant differences in the proportions of lymphocytes and monocytes among PBMCs and the proportions of T, B, NK, and NKT cells among lymphocytes (Fig 3). At seven days post-transplantation, the proportions of T, NK, and NKT cells were significantly decreased and those of B cells and monocytes were significantly increased in both groups compared with pre-transplantation. Thereafter, T cells, B cells, and monocytes reached baseline values from one to six months post-transplant. Importantly, the proportion of NK cells among lymphocytes was significantly lower in the ATG6.0 group than in the ATG4.5 group at three and six months after transplantation (Fig 3D).

**Fig 4:**
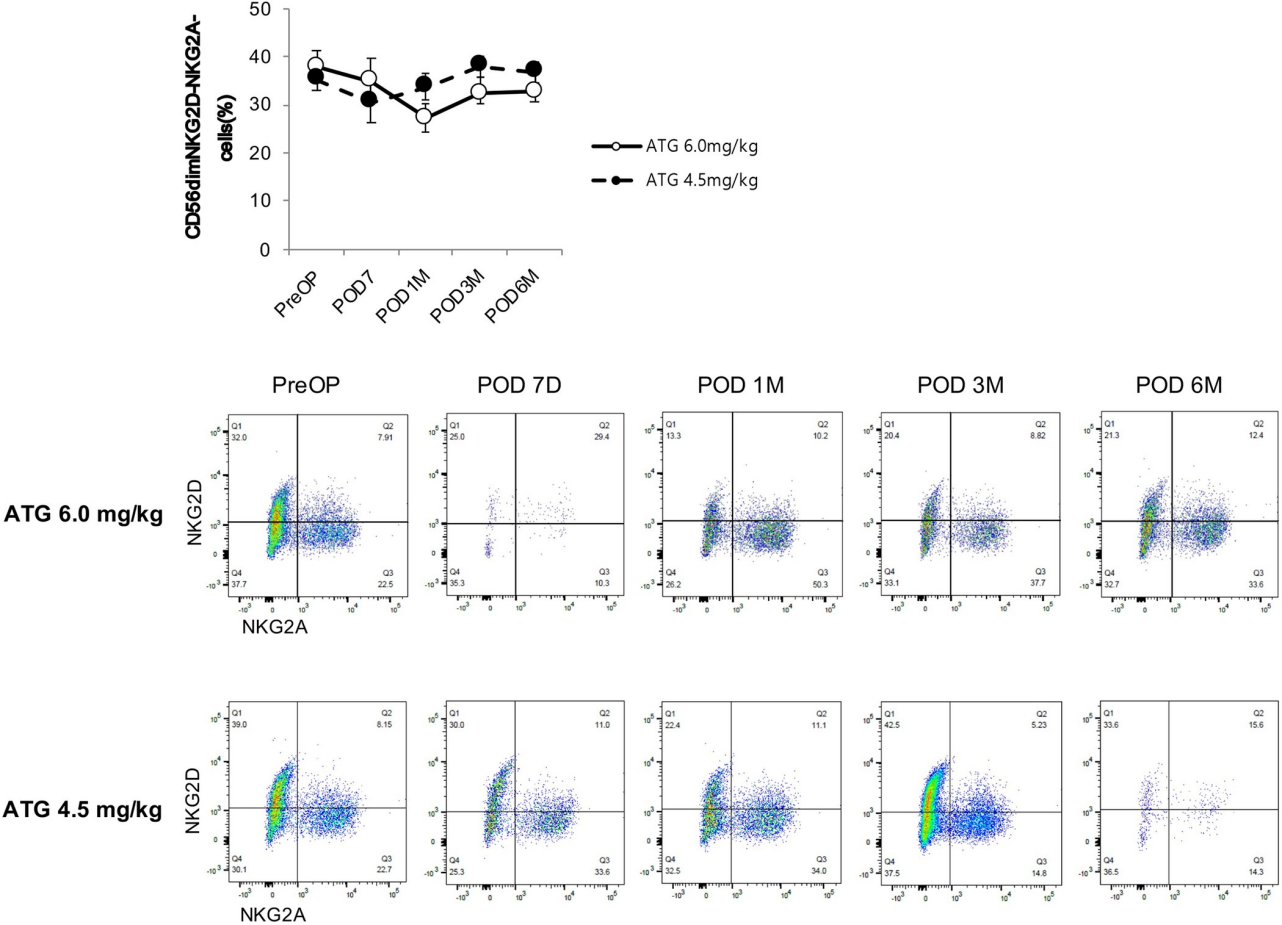
Proportion of NKG2D^-^NKG2A^-^ cells among CD56^dim^ NK cells according to the dose of anti-thymocyte globulin. ATG, anti-thymocyte globulin.

**Fig 5:**
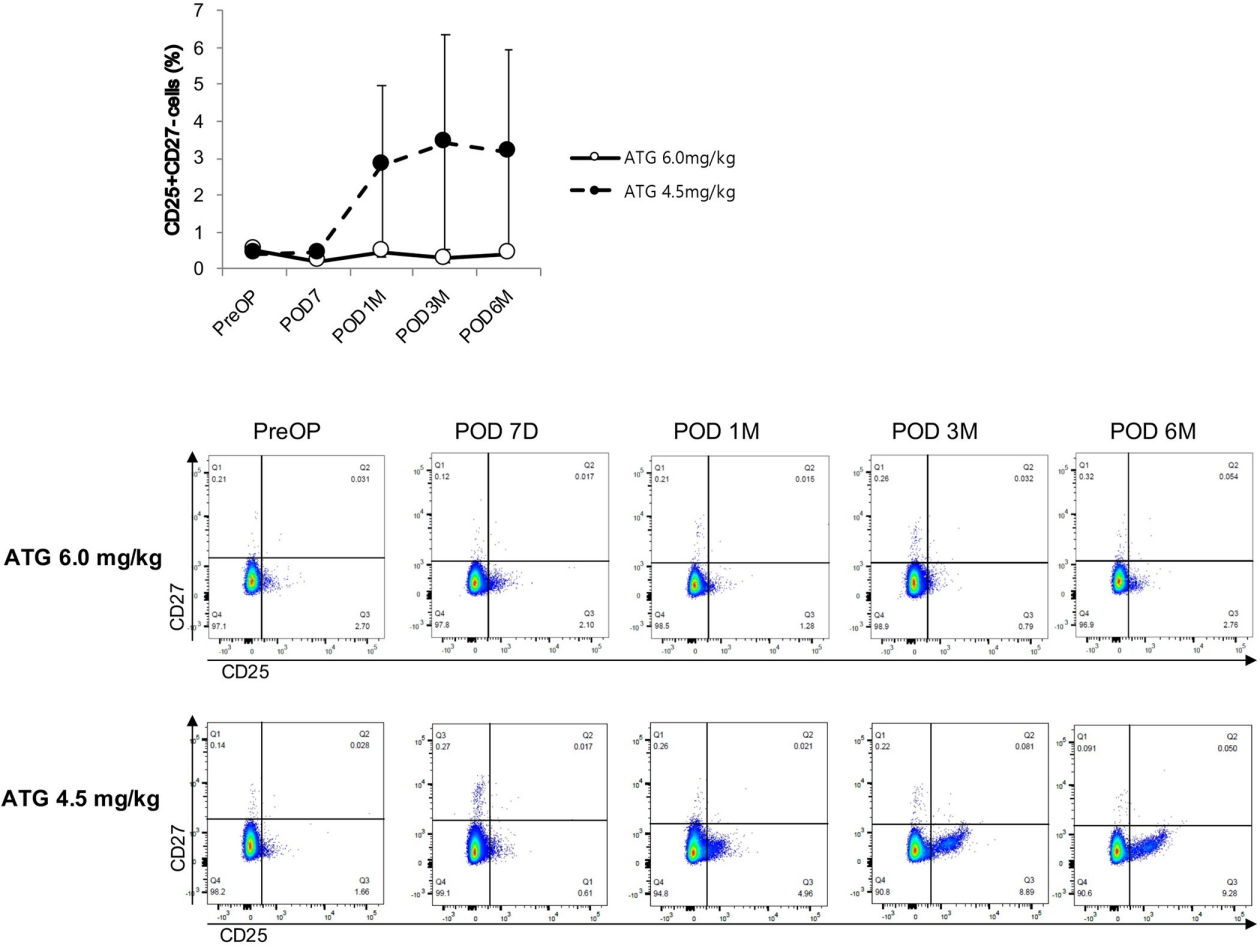
Proportion of CD25^+^CD27^-^ cells among CD3^-^CD11b^+^ cells according to the dose of anti-thymocyte globulin. ATG, anti-thymocyte globulin.

**Fig 6:**
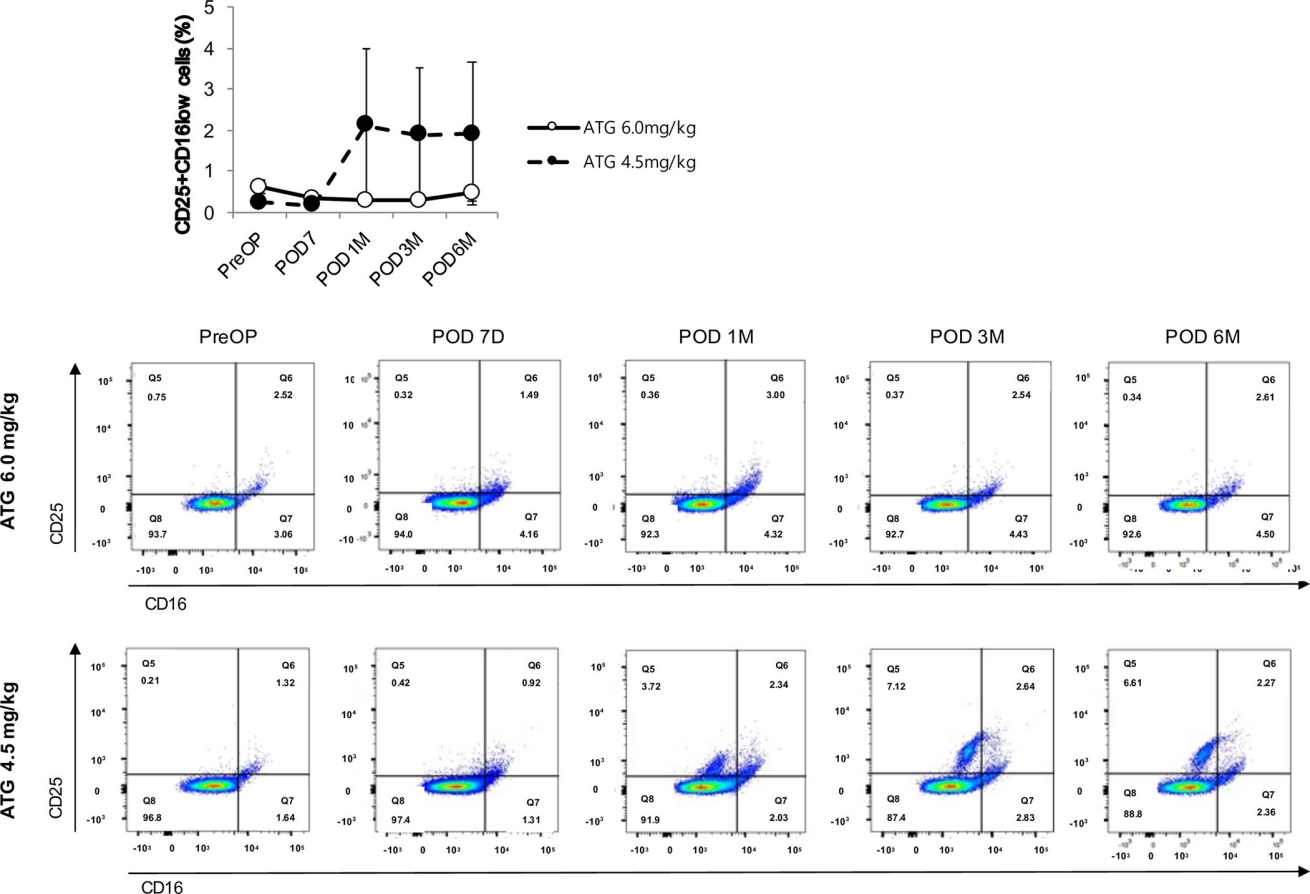
Proportion of CD25^+^CD16^low^ cells among CD3^-^CD11b^+^ cells according to the dose of anti-thymocyte globulin. ATG, anti-thymocyte globulin.

We performed flow cytometry analysis to identify the specific subsets of lymphocytes and monocytes that are differentially expressed according to the dosage of ATG. The linear mixed model revealed that the proportion of NKG2D^-^NKG2A^-^ cells among CD56^dim^ NK cells in the ATG4.5 group increased in a time-dependent manner compared with the ATG6.0 group (Fig 4). Furthermore, the proportion of CD25^+^CD27^-^ cells (Fig 5) and CD25^+^CD16^low^ cells (Fig 6) in the ATG4.5 group also increased in a time-dependent manner compared with the ATG6.0 group.

## Discussion

Our study results showed that compared with kidney transplant recipients who received ATG 6.0 mg/kg as an induction regimen with early steroid withdrawal, those who received ATG 4.5 mg/kg were more likely to show composite outcomes of biopsy-proven acute rejection, *de novo* donor-specific antibody formation, and graft failure. Furthermore, the proportion of NK cells among lymphocytes was significantly lower in the ATG6.0 group than in the ATG4.5 group during follow-up, whereas those of T, B, NKT cells, and monocytes were similar between the two groups. As far as we know, this is the first randomized trial to compare the clinical outcomes and immunologic profiles according to the dose of ATG in Asian kidney transplant recipients.

The dose of ATG ranged from 5.4 to 10 mg/kg in previous randomized trials that compared ATG with IL-2 receptor antagonist as an induction regimen in kidney transplant recipients [6,24–28]. On the other hand, the dose of ATG in randomized trials of ATG induction therapy with steroid withdrawal versus standard steroid therapy in kidney transplant recipients ranged between 5 and 6 mg/kg [29,30]. Recently, several studies evaluated the efficacies of lower dosages ATG not only on clinical outcomes but also on peripheral immune cells. Büchler et al. showed that 6.0 mg/kg ATG induced significant depletion and subsequent reconstitution of CD3^+^, CD4^+^, and CD8^+^ T cells and CD3^-^CD56^+^ NK cells, while not showing significant effects on the population of B cells [28]. In a randomized pilot study by Grafals et al., low dose ATG (2.25 mg/kg) with early steroid withdrawal seemed to be efficacious in depleting T cells and preventing acute rejection in low immunological-risk kidney transplant recipients [1]. In addition, Kho et al. compared the post-transplant T, B, and NK cell counts of three ATG dosage groups (1.5, 3.0, and 6.0 mg/kg) to those in control patients who did not receive an induction regimen [11], and showed that the 6.0 mg/kg group had significantly lower counts of T cells compared with the control group until one year post-transplant. After reviewing the above-mentioned literature, we decided to use ATG 4.5 mg/kg as the lower dose group to compare with ATG 6.0 mg/kg as the standard group in this randomized trial.

Our results are in line with those of previous reports that the degree of T cell depletion and reconstitution did not significantly differ according to ATG dosage [11,31] and that the changes in B cell population after ATG administration were minimal [32–34]. Conversely, our results showed that the NK cell population regained baseline level after six-months follow-up only in the lower ATG group, which was significantly higher than that in the standard ATG group. Furthermore, the composite end point of biopsy-proven acute rejection, *de novo* donor-specific antibody formation, and graft failure occurred more frequently in the lower ATG group. It is yet to be confirmed whether the higher incidence of the composite end point in the lower ATG group can be attributed to the earlier reconstitution of NK cells. Yet, recent reports using microarray transcriptomic analysis showed that NK cells are actively involved in the pathophysiology of ABMR and graft failure after kidney transplantation [35–37]. Therefore, it is possible that NK cells are involved in the differences in the prevalence of the composite outcome in our study. Specifically, we found that the proportion of NKG2D^-^NKG2A^-^ cells among CD56^dim^ NK cells in the lower ATG group increased in a time-dependent manner. It has been reported that CD56^+^NKG2A^+^ and CD56^+^KIR^+^ cells inactivate NK cells by interacting with HLA-E and distinct sets of classical HLA class I molecules [38–40]. Also, chronic engagement of NKG2D can reduce responsiveness of both NK and T cells for several activating receptors, thus suggesting a role of NKG2D in the induction of peripheral tolerance [41]. In other words, the lack of NKG2D can affect the development of NK cells in the bone marrow, resulting in hyperreactive NK cells [42]. In addition, CD25^+^CD27^-^ cells and CD25^+^CD16^low^ cells also showed a time-dependent increase in the lower ATG group. Duggleby et al. reported that, of the markers associated with freshly isolated regulatory T cells, only the expression of CD27 correlated with the regulatory activity and could be used to isolate cells with regulatory activity from lines expanded from CD4^+^ CD25^+^ cells. Also, cells expressing high levels of the transcription factor forkhead box P3 were confined to the CD27^+^ population within these lines [43]. It is well-known that CD16a, along with donor-specific antibodies, triggers the release of interferon-gamma and antibody-mediated NK cell-mediated cytotoxicity [36]. Therefore, it is possible that CD25^+^CD27^-^ cells and CD25^+^CD16^low^ cells contributed to the composite outcomes.

The following limitations of our study should be addressed. First, this study was ceased earlier than expected due to concerns regarding the observed risks of the composite outcome in the ATG4.5 group; also, the number of enrolled patients was less than the designated sample size. This limitation stands out because the study cohort were low risk live donor transplants in which the targeted events would be predictably low frequency compared to higher immunological risk or deceased donor subjects. Therefore, our results fall short of being able to draw confirmative conclusions. Second, only for-cause biopsies were performed in this study. To exclude subclinical rejection, it would be better if a protocol biopsy was performed. Third, the occurrence of early composite outcomes might have affected the reconstitution of immune cells because physicians often re-used steroids when patients developed the composite outcomes. Fourth, the trough level of tacrolimus and the MMF dose were relatively low which could have contributed to a higher rate of composite outcomes in the ATG4.5 group. Fifth, we could not find significant differences in the proportion of specific immune cells at each time between the groups which may be due to the small number of PBMC samples.

In conclusion, we compared ATG 4.5 mg/kg regimen with ATG 6.0 mg/kg as an induction regimen in non-sensitized Asian recipients undergoing kidney transplantation from living donors, and found that ATG 4.5 mg/kg and early corticosteroid withdrawal in association with a low dose maintenance regimen resulted in higher rates of biopsy-proven acute rejection and *de novo* donor-specific antibody formation, albeit without statistical significance.

## Supporting information

S1 File Raw data of this study (SPSS). (SAV)

S2 File Raw data of this study (EXCEL). (XLS)

S3 File CONSORT checklist. (DOC)

S4 File The study protocol (Korean version). (DOCX)

S5 File Informed consent for clinical research (for living donors). (DOCX)

S6 File Comparison of estimated GFR after kidney transplantation according to the dosage of ATG. ATG, anti-thymocyte globulin; GFR, glomerular filtration rate.
(TIF)

S7 File Gating strategy using single color compensation controls. (PDF)

S8 File The study protocol (English version). (DOCX)
