# Supplementary material for: A prospective, randomized, non-blinded, non-inferiority pilot study to assess the effect of low-dose anti-thymocyte globulin with low-dose tacrolimus and early steroid withdrawal on clinical outcomes in non-sensitized living-donor kidney recipients
Source: PLoS One. 2023 Mar 1;18(3):e0280924. doi: 10.1371/journal.pone.0280924 (PMC9976999; doi:10.1371/journal.pone.0280924)
Supplement: S4 File — (DOCX) [file pone.0280924.s004.docx]

The clinical trial protocol

**Title: A prospective randomized controlled study to evaluate feasibility and safety of early steroid withdrawal after 6mg/kg vs 4.5mg/kg Thymoglobulin^®^ induction therapy in living donor kidney transplantation.**

Protocol No. 2016V5.3

Phase Phase IV IIT (Investigator initiated Trial)

Principle Investigator Duck Jong Han

Collaborator Young Hoon Kim

Collaborator Sung Shin

Collaborator Youngmin Ko

Collaborator Hyunwook Kwon

Collaborator Yu-Mee Wee

Collaborator Joo Hee Jung

**Summary of the protocol**

| Name of the clinical trial | A prospective randomized controlled study to evaluate feasibility and safety of early steroid withdrawal after 6mg/kg vs 4.5mg/kg Thymoglobulin^®^ induction therapy in kidney transplantation |
| --- | --- |
| Purpose of the clinical trial | The aim of this study was to compare the efficacies of 4.5 mg/kg ATG and 6.0 mg/kg ATG in non-sensitized living-donor kidney recipients with early steroid withdrawal in an Asian population, and to investigate the immunologic profiles thereof during follow-up. |
| Design of the clinical trial | This was a prospective, open-label, randomized, non-inferiority pilot study in living- donor kidney transplant recipients at Asan Medical Center (Seoul, South Korea) |
| Sample size | 154 |
| Study period | Recruitment of participants : 24 months, Follow up : 12 months |
| Drug | Thymoglobuline® (Rabbit Anti-thymocyte globulin) |
| Other immunosuppressants | Tacrolimus, Corticosteroids, Mycophenolate mofetil (or EC-MPS) |
| Inclusion criteria | • Patients above 18 and below 70 years of age who were prepared for living donor kidney transplantation  • Patients who have the ability and will to consent to participation in this study. |
| Exclusion criteria | • A multi-organ transplant recipient  • A panel-reactive antibody of more than 20% or pre-transplant donor-specific antibody  • A recipient who prepared for ABO- or HLA-incompatible kidney transplantation  • A recipient who had a kidney allograft from an HLA-identical donor  • A recipient who had re-transplantation  • A recipient who had a known contraindication to the administration of ATG. |
| Methods | Patients are randomly assigned to receive either 4.5 mg/kg or 6.0 mg/kg of ATG; all patients have corticosteroid withdrawal within 7 days. |
| End points | The aim of this study was to compare the efficacies of 4.5 mg/kg ATG and 6.0 mg/kg ATG in non-sensitized living-donor kidney recipients with early steroid withdrawal in an Asian population, and to investigate the immunologic profiles thereof during follow-up.  **The primary efficacy end point**  - A composite of biopsy-proven acute rejection (BPAR), de novo donor-specific antibody formation, and graft failure  **The secondary efficacy end point**  - Renal function determined by eGFR (CKD-EPI) at one and six months post-transplant and one and two years post-transplant.  **The safety end points**  - Infection, leukopenia, thrombocytopenia, and malignancy |

1. **연구 배경**

신장이식 후 급성 거부 반응을 예방하기 위해서는 면역억제제가 필수적이며, 대표적인 면역 억제제로는 Tacrolimus 나 Cyclosporin같은 CNI 제제, Steroid 제제, Mycophenolate mofetil (MMF) 와 같은 anti-metabolite 가 있다. CNI 제제는 거부반응 예방에 매우 효과적인 것으로 알려져 있지만 사용 기간 및 농도에 따른 신독성이 알려져 있고, Steroid 제제 역시 다양한 부작용이 알려져 있다.

급성 거부 반응을 예방하기 위한 또 하나의 방법으로는 유도요법을 사용하는 것이고, 현재 국내에서 사용하는 유도요법 제제는 IL-2 R 억제하는 Simulect 와 polyclonal Ab인 Thymoglobuline®를 사용하고 있다. 신장이식에서 유도요법의 이점으로는 급성 거부반응의 빈도를 줄일 수 있고, 수술 후 사용하는 면역억제제 (특히 CNI 제제) 의 용량을 줄여 부작용을 줄일 수 있다.

또한 potent 한 유도요법 제제인 Thymoglobuline®를 사용하여 이식 후 스테로이드를 줄이려는 시도는 많이 있었고 좋은 결과를 보고하고 있다.

하지만 대부분의 연구가 해외의 보고로 한국인에게 그 용량을 그대로 적용 할 수 있는지는 미지수이다.

이에 본 연구는 신장이식 후 Thymoglobuline® 유도요법을 통한 steroid 중단 요법 시 Thymoglobuline® 의 용량을 달리 할 경우 그 유효성 및 안전성을 비교 평가하기 위함이다.

1. **연구 목적**

일차목표

-신장이식 후 12개월 까지 조직학적으로 확인된 첫 번째 급성 거부반응, 이식 신장 기능 지연, 이식 신장 소실, 사망으로 조합된 사건 발생률

이차 목표

-거부반응의 조직학적 강도(Banff criteria))

-12개월까지 overall rejection 발생률

-12 개월 시점의 steroid free 비율

-12 개월간의 신기능 (GFR)

-12 개월간의 혈액 내 면역 세포 변화, 소변 내 여러 단백질, 싸이토카인의 변화 관찰

-CMV, BK virus 발현 빈도

-약물치료가 필요한 감염 발현 빈도 및 중증도

-Hematologic AE 빈도(anemia, Leukopenia, thrombocytopenia)

-NODM 발생률.

1. **연구설계**

3.1 피험자 참여 기간

연구에 등록된 피험자는 신장이식 수술 이후 12개월(365일 ± 30일)까지 관찰을 요한다.

3.2 임상 연구 기간

IRB 연구 승인 이후36 개월 (피험자 모집기간 24개월, 치료기간 12개월)3.3임상 연구 기간의 정의 및 활동

**스크리닝/베이스라인:** 피험자가 임상 연구에 동의한 시점부터 최종적으로 연구의 피험자 적합성 평가 및 베이스라인 검사가 완료되어 신장이식 수술 시행 직후까지로 정의되며, 임상연구 계획서와 관련된 어떤 과정도 피험자가 동의서에 서명한 이후에만 진행할 수 있다. 연구자는 환자의 임상 연구 대상 포함 여부의 결정을 선정/제외 기준의 에 따라 시행한다. 적합성에 대한 최종 평가는 환자가 동의서 작성 후 가능한 한 빠른 시간 내에 실시해야 하며, 임상 연구에 동의한 시점부터 12주가 경과하는 경우 새로이 동의 절차를 진행한다. 베이스라인 검사는 임상적인 경과 변동이 없는 한 신장 이식 수술 전 12주 이내 검사를 반영할 수 있다. (PRA 검사는 1년)

시행항목: *피험자 동의서, 선정/제외기준 확인, 수혜자 배경정보, 공여자 배경정보, 신장이식 수술 정보, 과거력 정보, 활력 징후, 이학적 검사, 병용 약물 투여기록,* ***혈청 바이러스 검사, 임신 검사,혈액학 검사, 혈액화학 검사, 지질검사, 당화혈색소 (HbA1c), PRA****검사*

- 임상 연구 약물 투여에 따른 면역 세포 농도와 단백질, 싸이토카인 변화 정도 확인을 위한 임상 연구용 채혈 및 채뇨: Thymoglobulin 투여에 따른 면역세포 (T세포, B세포, NK세포) 농도 및 성상 변화를 확인하기 위하여 수술 전, 수술 후 1주일, 1개월, 3개월, 6개월, 12개월 각각 임상 연구용 혈액 (Heparin bottle에 10 cc)과 소변 (무균병에 20cc)을 하고 아산생명과학 연구소에 의뢰하여 분석한다. 본 연구를 위한 분석 후 혈액과 소변은 모두 폐기한다.

**방문 1 (POD 14일, ± 7일):**피험자가 신장이식 수술을 시행 받고 14일± 7일 째 방문까지의 기간으로 정의하며, 면역억제 요법 및 평가항목을 시행한다.

시행항목: *활력증후, 이학적 검사, 병용약물 투여기록,* ***혈액학 검사, 혈액 화학 검사, 지질검사, 소변검사,*** *면역 억제제 투여 기록, 경구 혈당강하제 또는 인슐린 투여기록,CNItrough level, 이식편 거부 반응, 신생검 기록, (중대한) 이상반응, 감염, 이식 후 투석, 이식편 소실*

**방문 2 (POD 1개월, ± 7일):**피험자가 신장이식 수술을 시행 받고 30일± 7일 째 방문까지의 기간으로 정의하며, 면역억제 요법 및 평가항목을 시행한다..

시행항목: *활력증후, 이학적 검사, 병용약물 투여기록,* ***혈액학 검사, 혈액 화학 검사, 지질검사, 소변검사,*** *면역 억제제 투여 기록, 경구 혈당강하제 또는 인슐린 투여기록, CNItrough level, 이식편 거부 반응, 신생검 기록, (중대한) 이상반응, 감염, 이식 후 투석, 이식편 소실*

**방문 3 (POD 3개월,± 14일):**피험자가 방문2(1개월 ± 7일)이후부터이식 수술 후 3개월 ± 14일 째 방문까지의 기간으로 정의하며, 면역억제 요법 및 평가항목을 시행한다.

시행항목: *활력증후, 이학적 검사, 병용약물 투여기록,* ***혈액학 검사, 혈액 화학 검사, 지질검사, 소변검사, 당화혈색소 (HbA1c),*** *면역 억제제 투여 기록, 경구 혈당강하제 또는 인슐린 투여기록, CNItrough level, 이식편 거부 반응, 신생검 기록, (중대한) 이상반응, 감염, 이식 후 투석, 이식편 소실*

**방문 4 (POD 6개월 ± 14일):** 피험자가 방문 3(3개월± 14일) 이후부터 이식 수술 후 6개월 ± 14일 째 방문까지의 기간으로 정의한다.

시행항목: *활력증후, 이학적 검사, 병용약물 투여기록,* ***혈액학 검사, 혈액 화학 검사, 지질검사, 소변검사,*** *면역 억제제 투여 기록, 경구 혈당강하제 또는 인슐린 투여기록, CNItrough level, 이식편 거부 반응, 신생검 기록, (중대한) 이상반응, 감염, 이식 후 투석, 이식편 소실*

**방문 5 (POD 9개월 ± 30일):** 피험자가 방문 6(6개월± 14일) 이후부터 이식 수술 후 9개월 ± 30일 째 방문까지의 기간으로 정의한다.

시행항목: *활력증후, 이학적 검사, 병용약물 투여기록,* ***혈액학 검사, 혈액 화학 검사, 지질검사, 소변검사,*** *면역 억제제 투여 기록, 경구 혈당강하제 또는 인슐린 투여기록, CNItrough level, 이식편 거부 반응, 신생검 기록, (중대한) 이상반응, 감염, 이식 후 투석, 이식편 소실*

**방문 6 (POD 12개월 ± 30일):** 피험자가 방문 4 (6개월 ± 14일) 이후부터 이식 수술 후 12개월 ± 30일 째 방문까지의 기간으로 정의한다.

시행항목: *활력증후, 이학적 검사, 병용약물 투여기록,* ***혈액학 검사, 혈액 화학 검사, 지질검사, 소변검사, 당화혈색소 (HbA1c),*** *면역 억제제 투여 기록, 경구 혈당강하제 또는 인슐린 투여기록, CNItrough level, 이식편 거부 반응, 신생검 기록, (중대한) 이상반응, 감염, 이식 후 투석, 이식편 소실*

1. 피험자

선정기준

18세 이상 70세 이하의남성또는여성 말기 신부전증 환자

뇌사 및 심장사나, 비 혈연간또는혈연간생체기증자로부터신장을이식받을 환자

임상연구 참여에 동의할 수 있는 능력과의지가있고 연구 동의서에 적절한 절차에 따라 서명하였으며,연구 계획대로 방문하여 임상연구에참여할수있는환자

제외기준

신장이외복합장기또는 2개의 신장을 이식 받을 예정이거나, 또는 이전에 기타 장기이식 (췌장, 심장, 폐장, 골수, 줄기세포)을 받은 또는 받을 환자

PRA 가 50% 이상인 환자 (DSA 양성인 환자 :single bead PRA를 시행 할 경우)

ABO혈액형불일치기증자의신장이나임파구 교차시험 (LCM, lymphocyte cross-match) 양성공여자의신장을이식받을환자

최근 5년 이내 암진단을 받은 적이 있는 환자(치료 완료된 squamous cell 또는 basal cell carcinoma 피부암 제외)

활동성 감염이 있는 환자

HLA 형이 동일한 기증자로부터 이식을 받을 환자

HIV, HBsAg 또는항-HCV RNA 검사결과가양성인공여자로부터신장을이식받거나 수여자가 양성인 환자

임상연구에사용되는의약품이나유사한화학구조를갖는의약품에대해급성 (최근 4주이내의) 혹은만성적인치료를요하는심한알러지나과민성의이력이있는경우또는 ATG 투여 금기가 있는 경우

연구등록전 30일이내에다른임상연구약물을투여받은환자

임신을계획중인임신가능성이있는여성, 임신한그리고/또는수유중인여성, 연구 기간 효과적인피임법을사용할의도가없는여성

조절되지않은질환을가지고있거나지속적인치료가필요한의학적상태

최근 24주 내에 알코올 또는 약제에 중독된 기왕력이 있는 환자

신장이식 수술 전 평가 시 다음과같은상태가있는환자

- 간기능 검사 (AST, ALT, ALKP, total bilirubin) 수치 중 두 항목 이상 정상 범주 보다 3배 이상 증가한 경우

- 절대호중구수치가<1,000/mm3이거나백혈구수치가<3,000/mm3, 또는혈소판수치가<75,000/mm3인환자

- 기증자 나이가 65세 이상인 경우

**※**가임기 여성의 임신 및 피임에 관한 사항: 60 세 이하의 폐경 이전 여성 피험자가 참여하는 경우에는 임상연구 시작 시점에 소변 또는 혈청 임신반응검사를 실시하고 검사 결과가 음성인 여성만 연구에 참여하며, 검사 결과는 임상시험용 약 복용전에 확인할 수 있어야 한다. 임신이 가능한 모든 여성은 신뢰할만한 피임법을 임상연구 전 기간 동안 지속해야 한다. 신뢰할만한 피임법은 완전 금욕 또는 자궁 내 장치나 기타 에슈어, 콘돔, 질격막, 실드, 캡, 스폰지 및 살정제가 포함된다. 주기적인 금욕(예, 배란 주기법, 증상 체온법 등)이나 체외사정 등은 신뢰할만한 피임법에 포함되지 않는다. 일부 병용될 수 있는 타 면역억제제는 경구용 피임약의 피임 효과를 감소시킬 수 있으므로 모든 종류의 호르몬 피임법은 본 연구 기간에 허용되지 않는다. 폐경후 여성(자연적인 무월경 기간이 12 개월인 경우)은 피임을 하지 않고도 참여할 수 있다.

목표 피험자 수 산정 근거

Composite end point (mortality, graft failure, biopsy proven acute rejection, delayed graft function)를 기준으로 이전 보고(Brennan DC, Daller JA, Lake KD, et al. N Engl J Med 2006; 355: 1967-1977)에서의 발생률 50.4%, 본원에서의 후향적 분석 자료를 바탕으로 평가했을 대 실험군에서의 발생률 40%로 정하고 Non-inferior study로 산출하였을 때 대조군과 실험군의 연구대상자 수는 각각 77명/77명이 된다.

1. 치료

5.1 연구약제

**연구약물**:

Thymoglobuline® (ATG) : Rabbit anti-thymocyte globulin

**투여일정:**

Dose : 1~1.5mg/kg/dose (Total 6mg/kg vs. 4.5mg/kg)

Frequency : 수술일 기준 0,1,2,3,4,5일째

첫 투여는 수술일 관류 전에 투여를 시작 한다.

체중은 수술 전 체중을 기준으로 한다

- 1. 병용 면역억제제:

CNI제제

- Tacrolimus를 사용한다.
- 이식 수술 전 후 2일 이내 (이식 수술 전 2일부터 이식 수술 후 2일 이내) 에 초기 경구 용량0.05 mg/kg BID 로(권장) 시작하여 기저치 혈중농도 (C0: trough blood level) 도달목표를 수술후 3개월 이내는 7-10 ng/mL, 3-12 개월 이내는 5-8 ng/mL, 수술 후 12개월 이후에는 4-7 ng/mL 로 유지한다.

코티코스테로이드(methylprednisolone / prednisone)

- 모든 피험자에게 신장이식 수술당일methyl-prednisolone 500 mg, 수술 후 1일째 250 mg, 수술 2일째 125mg, 수술 3일째 60mg, 수술 5일째 40mg, 수술 6일째 20mg 정주점적 투여하는 것을 권장하고 center 마다 조절 가능하다.
- DGF 등의 경우 연구자 판단으로 연장 사용이 가능하지만 14일 이내에 중단하여야 한다

Antimetabolite

- MMF (Mycophenolate Mofetil) 또는 Myfortic (enteric-coated mycophenolate sodium)를 권장함
- 이식 수술 전 후 2일 이내 (이식 수술 전2일부터 이식 수술 후 2일 이내) 에 MMF (Mycophenolate Mofetil) 500 mg – 1,000 mg BID 경구 투여하며, 신장 임상 경과에 감량 또는 증량한다. Myfortic(Enteric Coated Mycophenolate Sodium)은 mycophenolate mofetil용량의 0.72배로 계산하여 처방하여 복용 한다. 예를 들어, mycophenolate mofetil 500 mg BID 복용하던 환자는Myfortic® 360 mg BID 로 전환한다.
- 연구약인Thymoglobulin® 투여 전 Anti-histamin 과 acetaminophen을 30분 전 투여한다 (연구 기관의 통상적인 절차에 따라 처방되어 투약한다)

5.3 치료군 배정

본 임상시험은 무작위배정 (Block Random)에 의해 시험군/대조군 배정한다.

5.4 병용 면역 억제제 처방과 복용에 대한 사항

연구자는 환자가 각 방문 기간을 충족할 수 있는 적절한 수량의 약제를 분실이나 훼손, 예약 상의 문제 등을 대비한 추가 분을 감안하여 면역 억제제를 처방한다. Tacrolimus 의생체이용율변화를막기위하여자몽이나자몽주스는자제하여야한다.

연구자는 병용 면역억제제를 처방한 대로 정확히 복용하도록 피험자를 지도하고 복약순응이 피험자의 안전과 연구의 유효성을 위해 필수적이라는 사실을 언급함으로써 복약순응도를 높여야 한다.

모든면역억제제약물은다음과같은예외를제외하고경구로투여된다:

a. Methylprednisolone은정맥주사한다

b.Thymoglobulin ® 은정맥주사한다;

c.위장관계 합병증으로 약제의 경구 투여가 불가능하거나, 약제의 흡수에 장애가 있으리라 판단되는 경우 한시적으로 면역억제 약제를 정맥 주사 하며, 정맥 주사 약제의 용량은 bioavailability 지표에 따라 결정한다. (예를 들어, Prograf® 을 정맥 주사하는 경우 경구 용량의 1/3 의 용량을 투여)

이식 신장 기능지연(DGF: delayed graft function)은 이식 수술 후 첫7일 이내 투석이 필요한 경우로 정의되며, 이 경우 Tacrolimus 용량을 감량 또는 일시적 중단을 할 수 있다.5.5임상약의 용량 조정 및 중단

연구에 등록된 환자는 연구약(Thymoglobulin)의 투여 기간동안 매일 일반 혈액 검사 시행 후 아래와 같은 기준에 맞추어 용량을 조적한다.

- 백혈구> 3,000 cell/mm3 : 정량 투여

3,000> 백혈구 > 2,000 :1/2 감량 투여

2,000> 백혈구 : 투여 중단

- 혈소판 > 75,000 cell/mm3 : 정량 투여

75,000> 혈소판 > 50,000 : 1/2 감량 투여

50,000> 혈소판 : 투여 중단

피험자가 연구에 등록되어 CNI 및 antimetabolite 복용중 선택적인수술이필요하거나 연구 과정 중 응급 수술이 진행되어CNI 및 antimetabolite 를 시험자의판단에따라피험자의 연구등록을 유지하면서 최대14일동안정맥 주사, 감량 또는 중단할수있다.

연구계획서에 명시된 면역억제제 (Tacrolimus, MMF 또는Myfortic®, Azathioprine등) 으로 인하여 표준치료에 반응하지 않는 중등도 이상의 혈소판 감소증, 빈혈, 백혈구 감소증. 기타 심각한 이상 반응이 있는 경우 이에 대한 보편적이며 통상적인 치료를 하여야 한다. 보편적이며 통상적인 치료에 반응이 없거나 악화되는 과정이면 해당 면역억제제를 감량 또는 중단할 수 있다. 연구계획서에 명시된 면역억제제 일부를 감량 또는 중단할필요가 있는 경우 피험자는 연구에서 중도 탈락하고, 연구 기관의 표준치료로 전환한다.

본 연구의 임상약을포함하여 모든 병용 면역억제제는 의학적 사유 및 연구 계획에 의거하여 변경 투약 할 수 있다.

위와 같은 여러 가지 사유로 ATG 를 충분히 투여하지 못한 경우는(Thymoglobulin ® < 4.3mg/kg)Steroid 중단요법을 시행하지 않는다.

1. 병용약물 또는 치료

피험자의 병용 치료에 관한 모든 정보는 기록해야 하며,치료 명 또는 약물 명과 치료 기간 및 치료 사유가 포함되어야 한다. 모든 면역억제 요법Tacrolimus, MMF 또는 Myfortic® Corticosteroids기타 항체요법 등)은 증례기록 프로그램에 기록하며 약물 명, 총 1일 용량 및 치료 기간을 기록한다.

## 6.1 필수 치료

Pneumocystis infection 폐렴(PneumocystisCarinii)의 발병을 막기 위한 표준 예방법은 모든 피험자에게 신장이식 후 6개월이상 시행하며, 구체적인 예방법은 연구기관의 표준 예방법에 따른다.

거대세포바이러스 (cytomegalovirus(CMV)) 양성 기증자로부터 신장을 이식 받은 모든 CMV 음성 환자는 이식 후 CMV 감염 예방을 위한 표준 예방 요법을 받아야 한다.

## 6.2 권장 치료

guidelines 에 의거한 고지혈증 및 고혈압 치료.

입원기간 중 CMV prophylaxis 및 CMV monitoring(CMV antigenemia)을 통한 CMV infection시 CMV 치료.

BK monitoring(Blood PCR)을 통한 면역 억제제 감량 치료

## 6.3 허용 치료

다른 선행 또는 응급 질환의 치료.

수술중 및 수술후 표준 약물 요법. (예방적 항생제 및 진균 예방 요법)

진균감염의치료를위해 fluconazole 사용은허용된다. 이때 CNI 혈중농도 변화에더욱유의해야하고용량의감량을요할수도있다.

금지치료

- 다른 임상연구에 등록되어 투여되는 시험용 약물.
- 다음에 열거된강력한 cytochrome P450 유도제/억제제는 피험자의 임상 경과에 따라 연구자가 반드시 필요하다고 판단한 경우를 제외하고는 연구의 유효성 평가를 위하여 제한된다. 연구자의 판단에 따라 이들 약물은 신중히 사용될 수 있으며,약물 사용 시 Tacrolimus혈중농도를 검토하여 용량 조절을 필요로 한다.Tacrolimus 용량 조절에도 연구계획서에 제시된 범위의 혈중농도를 유지하기 불가능한 경우 피험자는 연구에서 중도 탈락하고, 연구 기관의 표준치료로 전환한다.

| A면역 억제제의약동학적변화를유발하는약제 | | |
| --- | --- | --- |
| Tacrolimus 의농도를  증가시킬가능성이높은약물 | | Tacrolimus 의농도를  감소시킬가능성이높은약물 |
| diltiazem*  nicardipine*  verapamil*  danazol  doxycycline  clotrimazole  metoclopramide  bromocriptine | erythromycin & derivatives  clarithromycin  telithromycin  troleandomycin  ketoconazole (except topical)  itraconazole  ritonavir  indinavir | carbamazepine  phenobarbital  phenytoin  rifampin  rifabutin  rifapentine  octreotide (경구용제형만해당함) |

Cytochrome P450 억제제는 Tacrolimus의 용량 감소를 용이하게 할 목적으로 사용될 수 없다.

구강 칸디다증 예방 목적으로 경구 또는 주사 경로로fluconazole을 전신 투여할 수 없다.

연구 시작 전에 중단되지 않은 aminoglycosides, amphotericin B, cisplatin 또는 그외의 신장기능저하와 연관된 약물 요법은 가급적 제한한다.

Tacrolimus 의생체이용율변화를막기위하여자몽이나자몽주스는자제하여야한다.

신장 이식 후 면역억제 치료기간 동안일반적으로 백신접종의효과가낮을수있으며,생백신 또는 독백신사용은금지된다.

사전치료

연구자는 신장 이식 수술 전 최소 4주동안환자가투여받은모든관련치료 또는 약물을규명하기위한최대한 노력한다.피험자의 모든관련정보는증례기록 프로그램에 기록해야하며,치료명및약물명과치료기간이포함되어야한다..

1. 급성 거부반응의 진단에 대한 진단 및 치료

거부 반응이 생겼다고 의심(명확한 이유 없이 creatinine 이 30% 이상 상승) 되는 모든 피험자에게는 임상연구자의 판단에 따라, 환자를 관리할 방법을 결정하기 위해 신장 생검, 신장 스캔, 신장 초음파 또는 기타 혈액학적 진단 처치를 시행할 수 있다. 급성 거부가 의심되는 경우에 생검이 금기가 아니면 진단을 확정하기 위한 치료를 시작하기 전에 우선적으로 생검을 시행해야 한다. 그러나 생검 결과가 나오기 전에 치료를 개시할 수도 있다. 만약 치료를 시작하기 전에 신장 생검을 할 수 없다면, 치료 개시 후 가능한 빨리(48시간 이내에) 생검을 시행해야 한다. 신장 동종이식 거부에 대하여07 Banff 기준을 사용하여연구 기관의 병리학자가 판독한다.

급성 거부반응은 해당 연구 기관의 진료 방침에 따라 치료를 한다. 급성 거부반응의 치료는 최소 3회에 걸쳐 최소500mg/dayIV methylprednisolone(생물학적동등 용량으로 투여 시 다른 스테로이드도 무방함)을 사용 한다. 첫 거부반응은 Steroid 를 사용하여 치료하고 연구자 판단에 따라 스테로이드 중단을 시도 해 볼 수 있다.

스테로이드저항성거부반응의경우 7-14일동안적절한용량으로항림프구치료를시작할수있다. 그러나, 스테로이드치료에부적절한반응이나타나거나항림프구치료시작이생검으로확증된급성거부반응으로부터 14일이상지연된경우, 생검을다시실시하여항림프구항체치료시작이전에현재의거부반응을확인하는 것이 바람직하다.

체액성 거부반응이 확인된 경우에도 항림프구치료를진행할 수 있으며, 혈장 교환술 (plasmapheresis), Rituximab 및 면역글로불린 (gamme-globulin)을 투여할 수 있다.

급성 거부반응 치료 중 심각한 감염이 발생하여 항생제, 또는 항진균제, 항 바이러스제의투여가 필요한 경우 해당 연구 기관의 표준치료를 받는다.

이식 신장의 중심부 생검 조직 검사 결과상 Banff 분류법 Grade III 이상 (Grade III 포함)의 소견이 있거나 스테로이드 내성 거부반응, 또는 치료기간 동안 2회 이상의 거부반응을 경험한 피험자는 임상연구를 중단하고 해당 연구 기관의 표준치료를 받는다.

1. 예상되는 약물 상호작용

8.1.CNI 약물 상호작용

Calcineurin inhibitors (Tacrolimus) 은 cytochrome P450 효소계에 의해 대사되므로 이들 효소를 억제하는 것으로 알려진 물질들은 두 약물 모두의 대사를 감소시켜 전혈이나 혈장 농도를 증가시키는 결과를 가져올 수 있다. 이들 효소계를 유도하는 것으로 알려진 약물들은 대사를 증가시켜 전혈이나 혈장 농도를 감소시키는 결과를 가져올 수 있다. CNI 농도를 증가시키는 약물들은 칼슘채널 차단제 (Diltiazem, Nicardipine, Verapamil), 항진균제 (Ketoconazole, Fluconazole, Itraconazole), 항생제 (Clarithromycin, Erythromycin), 글루코코티코이드, 기타약물 (Allopurinol, Bromocriptine, Danazol, Metoclopramide) 등이 있다. CNI 농도를 감소시키는 약물들은 항생제 (Naficillin, Rifampin), 항전간제 (Carbamazepine, Phenobarbital, Phenytoin), 기타약물 (Octreotide, Ticlopidine, Troglitazone) 등이 있다. 이와 같은 약물을 병용할 때에는 혈중농도 검사를 반영하여 적절한 용량 조절을 하여야 한다.

CNI 제제는 신독성이 있으며, 다음 약물은 상호 작용에 의하여 신독성이 가중될 수 있다. 또한, 특히 탈수 상태에서, 비스테로이드성 항염제(NSAIDs) 병용은 신기능 부전을 악화시킬 수 있다. 신기능 부전을 악화시킬 수 있는 약물들은 항생제 (Gentamycin, Tobramycin, Vancomycin 등), 항진균제 (Amphotericin B, Ketoconazole), 위장관계 악물 (Cimetidine, Ranitidine), 항암제 (Melphalan), 항염제 (Azapropazon, Diclofenac, Naproxen, Sulindac) 등이 있다.

CNI와 함께 투여했을 때 prednisolone, digoxin, lovastatin 그리고 simvastatin의 배출 감소가 관찰되었다. 또한, CNI 투여 후 digoxin 반감기의 명백한 감소가 보고되었다. Digoxin 을 투여하는 환자 몇 명에서 Tacrolimus 투여 시작 후 수일 이내에 중증의 디기탈리스 독성이 나타났다. 고칼륨혈증이 나타날 수 있기 때문에 Tacrolimus 는 칼륨보존성 이뇨제와 함께 투여할 때 신중하여야 한다.

CNI 치료기간동안, 백신접종은 효과가 낮을 수 있다. 생독백신 사용은 피해야 한다. Lovastatin 및 simvastatin과 병용시 근염, nifedipine과 병용시 종종 치은 증식, 그리고 고용량의 methylprednisolone과 병용시 경련이 나타날 수 있다.

8.2 MMF (Mycophenolate Mofetil) 또는 Myfortic®(enteric-coated mycophenolate sodium) 약물 상호작용

현재까지MMF (Mycophenolate Mofetil) 또는 Myfortic®(enteric-coated mycophenolate sodium)과 상호작용이 있는 약물로는 다음과 같은 것들이 알려져 있다.

Azathioprine (계획서에 허락하지 않는 면역억제제), tacrolimus, acyclovir, 수산화 마그네슘과 수산화 알루미늄을 함유하는 제산제, cholestyramine, probenecid와 같이 신장 세뇨관으로 분비된다고 알려진 약물 또는 위장관 내의 균(flora)을변화시키는 약물은 간-장간 재순환에 변화를 초래하여MMF (Mycophenolate Mofetil) 또는 Myfortic®(enteric-coated mycophenolate sodium)과 상호작용을 일으킬 수 있다. MMF 는 위산 분비 억제제와 병용 사용시 노출이 감소되는 것으로 알려져 있다. 일반적으로 MMF (Mycophenolate Mofetil) 또는 Myfortic®(enteric-coated mycophenolate sodium)의 제품 설명서에 명시된 주의사항을 따라야 한다.

1. 임상 연구약 중단

피험자는 어느 때, 어떤 이유로든 동의 철회 과정을 통하여 임상연구를 자발적으로 중단할 수 있으며, 연구 약제 복용을 중단할 수 있다.

연구 계획서에서 규정한 임상 연구 과정에서 연구약인 Thymoglobuline® 을중단하게 되는 사유가 발생하는 경우, 연구약인 Thymoglobulin ® 을투여한마지막 날짜를증례기록 프로그램에 기록하여 관리한다.

연구자는 다음 사유가 있는 경우 피험자의 연구 과정을 조기 중단할 수 있다.

1. 연구약으로 인하여 피험자의 안전에 유의한 위험을 야기할 것이라고 연구자가 판단하는 경우

- 중증과소 면역억제 징후(Banff G3 이상, 스테로이드 저항성 급성거부반응, 2회 이상의 급성거부반응)
- 중증과잉 면역억제
- 악성질환
- 임신

2. 이식편 소실 (소실 가능성이 있어 제외한 경우도 이식편 소실로 간주)

3. 사망

4. 추적 조사 실패 (무작위 배정 후 연구약을 한번 이라도 투약 받은 후 연구가 중단된 모든 경우를 포함함. )

1. 연구계획서에 명시된 면역억제 요법을 유지하기에 불가능한 경우의 피험자는 연구에서 중도 탈락하고, 연구 기관의 표준치료로 전환
2. 기타 유효성 실패와 무관한 행정적 문제, 동의 철회, 시험 절차 무시
3. 계획서 위반

연구 과정이 조기에 중단된모든피험자는가능한때에언제라도거부반응, 이식장기및환자생존율, 및면역억제약물에대한정보 등 안전성에 관한 추적조사하기위하여방문해야한다. 피험자가 사망하거나 또는 방문 예정에 내원하지 않거나, 연구 동의를 철회하거나 또는추적조사실패의사유로더이상시험을지속하지않는경우에는 임상연구를 중단한 것으로 간주한다.

방문하지 않는 환자나 추적 조사가 중단된 경우 전화 통화를 시도하거나 등기 우편을 보낸 날짜 등 피험자와 접촉하기 위해 취한 조치들을 근거 문서에 기록으로 남김으로써 “주의 의무”를 다한다.

피험자가 연구약인 Thymoglobulin ® 을계속 투여하면 피험자의 안전에 유의한 위험을 야기할 것이라고 연구자가 판단하는 경우 피험자는 연구약인 Thymoglobulin ® 을 중단할 수 있다. 연구약중단을 고려할 수 있는사유는 다음과 같다.

1) 중증 과소 면역억제의 징후

Banff 분류법 Grade III 이상의 생검으로 확인된 급성 거부 반응, 스테로이드 저항성 급성 거부 반응, 이식 신장 생검 소견과 관계없이 2 회 이상 (2회 포함)하는 급성 거부 반응

2) 중증 과잉 면역억제의 징후

중증의 전신 감염 발생(세균, 진균, 바이러스, 원생동물, 또는 미지 생명체)

조직 생검 또는 소변검사 등으로 확진된 BK polyoma 감염.

3) 악성질환(Malignancy)

4) 임신

이와 같은 Thymoglobulin ®의중단 요건과 함께 연구자가 이익과 위험을 고려해 볼 때 연구약을지속하는 것이 환자의 안녕에 해가 된다고 판단하는 경우 연구자는 이러한 환자에서 Thymoglobulin ®을 중단해야 한다.

임상연구 종료 시점인 신장 이식 후 12개월 이전에 임상연구를 중단하는 모든 피험자는 해당 연구 기관의 표준 치료 관행에 따라 다른 면역억제제로 전환할 수 있다. 추적조사 자료는 가능한 경우 이식 후 12개월 시점까지 수집한다.임상약 투여를 중단한 사실은 마지막으로 투여한 날짜와 중단의 주원인과 함께 증례기록 프로그램에 기록되어야 한다.

1. 임상시험 종료

임상연구를 조기에 종료한 경우가 아니라면 피험자는 신장이식 후 12개월 시점에 임상연구를 종료한다. 연구 기간 종료된 피험자의 연구 결과는 취합되어 증례기록 프로그램에 기록되며, 모든 연구에 관련된 자료는 해당 연구 기관에서 자료를 활용할 수 있다. 피험자의 개인 신상에 관한 정보 (이름, 주민등록번호, 주소, 전화번호, 등)는 비밀 보장이 준수 되어야 한다. 어느 시점에서든 피험자는 자발적으로 임상연구를 그만 둘 수 있고, 연구자는 연구 탈락의 사유 또는 연구약 중단의 사유가 있는 피험자를 임상연구에서 중도 탈락시킬 수 있다. 임상연구 종료 시점인 신장이식 후 12개월이지나 임상 연구를 종료한 피험자는 해당 연구 기관의 방침에 따른다.

임상연구를 종료하기 전에 어떤 이유로든 임상연구를 조기에 중단하는 경우 피험자는 가능한 한 빨리 방문을 예약하여 이 때 최종 방문 시에 열거된 모든 평가를 마칠 수 있도록 한다.

1. 방문일정 및 평가, 방문 일정별 면역 억제 치료

방문과 평가에 대한 일정은 “방문일정”을 참고한다. “V”표는 방문해서 실시하는 것을 표시한다. 피험자는 예정된 날짜에 모두 내원해야 하는데 방문1,2,3의 “방문 여유기간(visit window)”은 7일 (방문일 ± 7일)이며, 방문 4의 “방문 여유기간(visit window)”은 14일 (방문일 ± 14일)이다.

| **방문일정 및 평가항목** | | | | | |  |  |
| --- | --- | --- | --- | --- | --- | --- | --- |
| **시행항목** | **Screen/**  **Baseline** | **Visit 1 (D14)**  **±7** | **Visit 2 (M1)**  **±7** | **Visit 3 (M3)**  **±14** | **Visit 4 (M6)**  **±14** | **Visit 5 (M9)**  **±30** | **Visit6**  **(M12)**  **±30** |
| 피험자 동의서 | V |  |  |  |  |  |  |
| 선정/제외 기준 | V |  |  |  |  |  |  |
| 수혜자 배경 정보 | V |  |  |  |  |  |  |
| 공여자 배경 정보 | V |  |  |  |  |  |  |
| 이식수술 정보 | V |  |  |  |  |  |  |
| 과거력 정보 | V |  |  |  |  |  |  |
| 활력 징후 | V | V | V | V | V | V | V |
| 이학적 검사 | V | V | V | V | V | V | V |
| 병용 약물 투여 기록 | V | V | V | V | V | V | V |
| 바이러스 검사^[[1]](#footnote-1)^ | V |  |  |  |  |  |  |
| 임신 검사^[[2]](#footnote-2)^ | V |  |  |  |  |  |  |
| 혈액학 검사^[[3]](#footnote-3)^ | V | V | V | V | V | V | V |
| 혈액화학 검사^[[4]](#footnote-4)^ | V | V | V | V | V | V | V |
| 지질검사 (Cholesterol, TG, HDL, LDL) | V | V | V | V | V | V | V |
| 소변검사^[[5]](#footnote-5)^ | V | V | V | V | V | V | V |
| 당화혈색소 (HbA1C) | V |  |  | V |  |  | V |
| CNI 투여 기록 |  | V | V | V | V | V | V |
| Steroid 투여 기록 |  | V | V | V | V | V | V |
| MMF 또는 Myfortic® 투여 기록 |  | V | V | V | V | V | V |
| Thymoglobulin ® 투여 기록 |  | V |  |  |  |  |  |
| 기타 면역억제제 투여 기록 |  | V | V | V | V | V | V |
| 경구혈당강하제 또는 인슐린 투여 기록 | V | V | V | V | V | V | V |
| CNI 기저치 혈중농도 |  | V | V | V | V | V | V |
| 신생검 기록 (해당 사항의 경우) |  | V | V | V | V | V | V |
| 신생검 기록 (해당 사항의 경우) |  | V | V | V | V | V | V |
| (중대한) 이상반응 (해당 사항의 경우) |  | V | V | V | V | V | V |
| 감염 (해당 사항의 경우) |  | V | V | V | V | V | V |
| 이식 후 투석 (해당 사항의 경우) |  | V | V | V | V | V | V |
| 이식편 소실 (해당 사항의 경우) |  | V | V | V | V | V | V |
| 악성질환 (해당 사항의 경우) |  | V | V | V | V | V | V |
| 피험자 사망 (해당 사항의 경우) |  | V | V | V | V | V | V |
| 계획서 위반 (해당 사항의 경우) |  | V | V | V | V | V | V |
| 치료 종료 및 임상연구 종료 |  |  |  |  |  |  | V |

1. 연구약과 면역 억제제에 대한 노출 및 복약 순응도

임상연구 기간 중 복용 한 모든 면역억제제 (Tacrolimus/cyclosporine, steroid, MMF (Mycophenolate Mofetil) 또는Myfortic®(Enteric-Coated Mycophenolate Sodium), Thymoglobulin ® 등의 투약 정보는 증례기록 프로그램에 기록한다. 모든 면역억제제의 종류 시작 날짜, 용량, 중단 날짜, 복용 이유 또는 변경 이유를 기록한다.

임상연구 치료를 시작하기 전부터 복용하기 시작하여 임상연구 시작 시점까지 지속하고 있거나 임상연구 치료 기간 중 시작한 다른 약물은 증례기록 프로그램에기록한다. 시작 날짜, 중단 날짜, 복용 이유 또는 변경 이유를 기록한다.

방문 시마다 연구자나 연구 담당자는 복약순응도를 평가해야 한다.

1. 유효성 평가

연구약의 유효성을 평가하기 위하여 다음 항목에 대한 분석을 시행한다.

- 신장이식 수술 후 12 개월 까지의 BPAR (Biopsy proven Acute rejection)의 발생빈도
- 신장 이식 수술후12개월까지의 overall rejection 의 발생빈도
- 신장이식 수술 후 12 개월째 Steroid free (protocol success) rate
- 신장 이식 수술후12개월째에 예측 계산된 사구체 여과율
- 신장 이식 수술후12개월까지의 환자 및 이식편생존율.
- 신장 이식 수술후2 주 1, 3, 6, 12개월째소변 단백뇨
- 신장 이식 수술 후 DGF 발생빈도

이식 신장 기능

다음의유효성분석을 위하여수집되는 변수에 대한 구체적인 사항은 다음과 같다.

a. 혈청크레아티닌 : 혈청크레아티닌분석은정맥혈을채취하여기관의지역실험실에서분석한다.

b. 신사구체여과율 (GFR; glomerular filtration rate)

신사구체여과율(GFR)은이식 신장의 기능을임상적으로가장정확하게 가늠할 수 있는지표이며신기능장애의임상적중증도와상관관계가있다.

본 연구에서MDRD 법(MDRD, Modification of Diet in Renal Disease)에따라계산된 GFR 값을일차결과변수로사용한다.

거부반응

모든추정된거부반응은처음으로거부반응이의심된날짜, 생검실시여부, 추정조사실시여부(해당날짜도기록), 항거부반응치료여부, 급성거부반응이확인되었는지또는최종임상적진단이밝혀졌는지여부, 그리고최종임상적결과등을증례기록 프로그램에 기록한다.

연구자는 급성 거부반응으로 의심되는 상황에 대하여도 치료 내용과 함께 증례기록 프로그램에 기록한다. 급성 거부반응으로 의심되는 경우 이식편에 대한 생검을 실시하여야 하며, 생검 결과 확인 전에도 치료를 시작할 수 있다. 생검 결과는 연구 기관 내 병리학자가 Banff 07 분류법에 의거하여 판독하고 증례기록 프로그램 생검란에 기록한다. 유효성 및 안전성 분석 할 때에는 급성 거부반응 *Grade I이상* (Grade I 포함)으로 조직학적 확인되어 급성 거부반응에 대한 치료를 받은 경우 급성 거부반응으로 분류되어 분석된다.

거부반응은 1차 유효성 변수로 취급되는 사건으로서 이상반응으로 기록하지 않으며, 급성 거부반응 의심되었으나 다른 질환으로 판명된 경우에는 이상반응 (AE; Adverse Events)으로 기록한다. 임상적으로 추정되어 치료되거나생검을통해확인된급성거부반응이발생할때마다기록, 관리한다. 한건의거부반응발생으로부터 14일이상이후에나타난거부반응은새로운거부반응으로간주한다.

신장생검

신장생검은임상 경과에 따라 거부반응이 의심되거나 단백뇨, 기타 이식 신장의 상태 변화에 따라 연구자의 결정에 의하여 언제든지 진행될 수 있다.

모든추정된거부반응에서, 항거부반응치료시작이전이나치료시작후늦어도48시간이내에이식신 생검을실시한다. 생검을연구 기관의병리학자가판독하고해석한다. 결과는기록되고유효성분석에사용한다. 생검을통해확인된급성거부반응은Banff 분류 등급 IA, IB, IIA, IIB, 또는 III로구분한다.신장 생검을 시행한 경우 급성 거부반응이외 모든 소견도 기록하여야 한다.

임상적 거부반응

임상경과에서 거부반응이 의심되나 조직학적 진단을 할 수 없는 경우는 clinical rejection으로 진단하고 치료를 시작 할 수 있다.

이식편소실 또는 사망

이식편이 소실은 1) 피험자가 투석을 시작하고 그 후 투석을 중단할 수 없을 때, 2) 이식 신장 절제술을 시행하였을 때, 3) 적절한 영상기술에 의해 이식편의 혈류가 비가역적으로 중단되었다고 확인될 때로 정의되며, 투석을시작하여이후투석을중단할 수 없을 때 투석시작일에이식편이소실되었다고간주한다. 피험자가이식 신장절제술을실시하는경우, 신장절제술날짜를이식편소실일로한다. 또한 영상진단에 의하여 확인된 경우 영상 진단일자로 하며, 상기 3 항목이 중복 확인된 경우 가장 앞선 날자로 평가한다. 이식편소실의사유는 증례기록 프로그램에기록한다. 이식편소실은중대한이상반응으로간주되며증례기록 프로그램에도 기록하고 중대 이상반응 보고서를 작성하여 총 책임연구자에게24시간 이내에 팩스로송신해야한다. 이식 신장이 기능하며 환자가 사망한 경우 사망일자에 이식편 소실로 간주하여 이식편 생존을을 산출한다.

1. 안전성 평가

감염

감염 발생 시그동정균, 배양유무, 시작일, 소멸일, 최대강도, 보고방법, 경과, 경중, 결과, 인과관계, 예측가능성, 취해진 조치, 면역억제제의 조절 등을 기록 한다.

CMV 감염이란음성환자에서혈청전환(seroconversion)이나타나거나뇨, 타액, 혈액또는기타조직에서 CMV가분리된경우확진 될 수 있다. CMV 바이러스혈증의 근거가 있는 경우복합적으로 판단하여 치료를 결정할 수 있다. 치료의 시기 및 방법은 연구자의 판단에 따라 연구 기관의 표준 치료를 적용한다.

BK 감염이란소변 세포 검사상 Decoy cell이 확인되며, 소변 또는 혈청에서 정량 또는 정성 PCR (polymerase chain reaction) 양성이면 진단하고, 이식신생검에서 조직학적으로 확인되면 BKVN 으로 확진 될 수 있다. 임상적병인은없으면서혈청 크레아티닌 상승이 있는 경우 의심할 수 있으며 복합적으로 판단하여 치료를 결정할 수 있다. 치료의 시기 및 방법은 연구자의 판단에 따라 연구 기관의 표준 치료를 적용한다. 면역억제제의 일부 변경이 필요한 경우가 연구를 유지하면서 변경할 수 있다(leflunomide 등)

감염을 치료하기 위해 약물을 사용한 경우 약물의 명칭, 투여시작일, 투여중지일, 용량, 투여경로, 적응증 항목이증례기록 프로그램에 기록된다.

혈액학적 부작용의 발현빈도(Anemia, Leukopenia,thrombocytopenia)

종양의 발생 빈도

AE/SAE 발생 빈도

이상반응

이상반응이란 연구 약물 투여 시작 이후 바람직하지 않은 징후, 증상 또는 질환이 나타나거나 악화되는 경우를 말하며 반응이 연구약과 관련 있는 것으로 간주되지 않는 경우도 포함한다. 연구약이란 임상연구의 모든 단계에서 투여되는 평가대상인 연구약물, 대조약물 또는 위약으로 정의된다. 연구약 투여 시작 이전에 존재하던 의학적 상태/질환이 연구약 투여 시작 이후 악화되는 경우에만 이상반응으로 간주한다. 검사실 수치 또는 검사결과 이상은 이들이 임상적 징후 또는 증상을 야기하여 임상적으로 유의하거나 치료를 요하는 경우에만 이상반응으로 간주한다. 이상반응 발생은 연구기간 동안 각 방문 시 연구자에 의하여 피험자에게 문진 및 진찰, 검사를 진행하여 확인될 수 있으며, 방문 동안이나 방문 사이의 기간에 피험자가 자발적으로 보고하거나 신체 검사, 검사실 검사, 또는 기타 평가를 통하여 확인될 수도 있다. 모든 이상반응이 발생하는 경우 이상반응명, 시작일, 소멸일, 최대강도(경증, 중등도, 중증), 보고방법(질문, 측정, 관찰, 자발적 보고), 경과 (계속됨, 간헐적, 1회), 경중(심각하지 않음, 심각함), 결과 (회복 후유증 없음, 회복 후유증 있음, 부작용 존재 진행 없음, 부작용 존재 지속중, 사망, 추적관찰 불필요), 인과관계(확실함, 상당히 확실함, 가능함, 가능성 적음, 평가곤란, 평가불가), 예측가능성(예측된 부작용, 예측하지 못한 부작용), 취해진조치(취해진 조치 없음, 치료약물 병용투여, 비약물치료, 입원/입원기간의 연장), 시험약의 취해진 조치(취해진 조치 없음, 용량조절, 일시적 중단, 영구적 중단) 등의 정보를 평가하여 증례기록 프로그램에 기록되어야 한다:

이상반응이 나타나면, 해소되거나 영구적인 이상반응이라고 간주 될 때까지 추적되어야 한다. 중증도의 변화가 있는지 여부, 연구약물과의 추정되는 관련성, 치료하기 위하여 필요한 중재법, 그리고 그 결과에 대한 평가를 매 방문마다(또는 필요하다면 더 자주) 실시 한다. 연구약은 현재 시판되고 있는 약물로 이미 알려진 흔한 부작용 정보는 제품설명서에 기재되어 있다. 이러한 정보는 피험자 동의설명서에도 기재되어 있으며 필요한 경우 임상연구기간 동안 피험자와 논의 한다.

- 중대한 이상반응(SAE)은 어느용량에서든발생하는 모든 의도하지 않은 의학적 사건이며 다음에 해당하는 결과를 초래한 경우이다.
- 본 연구에서 중대한 이상반응(SAE; Serious Adverse Event)이란 다음과 같이 정의된다:
  - 치명적이거나 생명을 위협하는 경우
  - 지속적이거나 중대한 불구/무능력을 초래하는 경우
  - 선천적 결함/ 태아기형이 우려되는 경우
  - 입원이 필요하거나 입원기간 연장이 필요한 경우(아래의 경우 제외)
    - - 상태 악화와 관련되지 않은 정기치료 또는 모니터링
      - 연구의 적응증과 관련 없으며 연구 약 투여 시작 이후로 악화되지 않은 기존 질환의 선택적 또는 사전 계획상의 치료
      - 상기에 언급된 중대한 이상반응의 정의를 만족하지 않으며, 병원 입원이 필요 없는 응급 외래환자의 치료
      - 사회적 사유 또는 피험자의 전반적 상태가 악화되지 않은 상황에서 일시적 입원 조치
- 의학적으로 위급한 상태, 즉 피험자가 위험하거나 위에 명기한 사항들을 방지하기 위해 의학적 또는 외과적 처치를 필요로 하는 경우
- 이식편 소실

정기적인 안전성 평가와는 달리, 중대한 이상반응(SAE; Serious Adverse Event)에 대하여 지속적으로 모니터링 하며 별도의 보고 절차를 따른다. 중대한 이상반응이 발생하는 경우 연구 기관에서는 중대한 이상반응 보고 양식에 서면 기록하여 반드시 24 시간 이내에 책임연구자에게 보고하며, 책임연구자는 타 연구자에게 보고 한다.

**1.2안전성 보고에 대한 연구자의 의무**

**1.2.1 이상반응(AE)**

- 모든 이상반응(중대한 이상반응을 포함)의 안전성 관찰 기간은 환자가 피험자 동의서에 서명한 시점부터 시작된다.
- 피험자 동의서 서명일부터(즉, 시험약을 투여하지 않은 스크리닝 기간 중 발생했더라도) 투여 기간 중, 마지막 시험약 투여 후 30일까지 발생한, 중대성 또는 시험약과의 관련성 여부에 관계없이 모든 이상반응은 증례보고서의 해당페이지에 기록되어야 한다.
- 가능한 한, 증상보다는 단일 증후군 또는 진단명으로 보고되어야 한다. 연구자는 발현 날짜, 중증도, 시험약과 관련한 조치, 실시한 교정적 치료/요법, 추가적으로 실시한 검사, 이상반응이 시험약에 의해 발생하였다는 합리적인 가능성이 있는지 여부에 대한 시험자의 의견을 명시해야 한다.
- 이상반응으로 인하여 실시하거나 관련된 추가 절차 또는 임상시험계획서에 명시하지 않았으나 안전성 평가를 뒷받침하기 위하여 실시되는 추가 검사는 증례보고서에 기록하여야 한다.
- 연구 흐름도 상 시행하는 모든 실험실 검사들은 위의 이상반응 기준에 관계없이 증례보고서의 해당 페이지에 보고한다(혈액학, 생화학 등).
- 실험실 결과, 활력 징후 또는 ECG 비정상은 다음에 해당하는 경우에만 이상반응으로 기록한다:
- 증상이 있으며(and)/있거나(or)
- 교정적 치료 또는 협진을 요하며(and)/요하거나(or)
- 중대성 기준을 만족하며(and)/만족하거나(or)
- 시험약 투여 중단/용량 변경 혹은 연기를 요하는 경우

**1.2.2 중대한 이상반응(SAE)**

중대한 이상반응이 발생하는 경우, 연구자는 즉시 다음을 실시해야 한다.

*주의: 연구자가 중대한 이상반응을 인지한 지 24시간 이내에 IRB에 보고한다.*

기입(ENTER): 중대한 이상반응과 관련된 정보를 증례보고서의 해당 페이지에 기입한다.

이후의 모든 갱신된 자료는 증례보고서에 적절하게 기록되어야 하고 추가 문서와 정보(실험실 자료, 병용 약물, 피험자의 상태 등) 또한 인지한 지 근무일 1일 이내에‘추적 보고(Follow-up report)’로서 IRB에 보고한다. 사망 또는 생명의 위협에 해당하는 중대한 이상반응의 경우 최초 통지로부터 7일 이내에 추적 관찰이 실시되고 해당 추가 기록을 하도록 모든 노력을 취하여야 한다.

**1.2.3 추적 관찰**

- 시험자는 환자의 안전을 보장하기 위해 적절한 모든 조치를 다해야 하며 환자의 상태가 정상화될 때까지 그 결과를 추적 관찰해야 한다.
- 치료가 조기 중단된 환자의 경우, 임상시험계획서에 정의된 시험 종료일까지 해당 환자에 대한 관찰을 지속한다.
- 중대한 이상반응의 경우, 임상적 회복이 완전할 때까지 그리고, 실험실 결과가 정상으로 회복될 때까지 또는 진행이 안정화될 때까지 환자를 추적 관찰해야 한다.
- **1.2.4 임신 보고를 위한 가이드라인**

임신은 모든 경우 즉각적으로 보고해야 하는 신속 보고의 대상이며,‘이상반응’으로 기록하되 중대한 이상 반응의 기준을 만족할 시에만 ‘중대한 이상반응’으로 보고한다. 임신 발생 시 임상 시험용 의약품은 투여 중단되어야 하며 이상반응/신속 보고 양식에 따라 연구자의 인지일로부터 24시간 이내에 안전성정보관리 담당자와 책임연구자에게 보고되어야 한다. 임신에 대한 결과가 확인될 때까지 추적 관찰을 요한다.추적조사 내용에는 자연 유산 또는 인공 유산, 상세한 출산 내용, 선천적 기형의 유무, 출산 이상 혹은 산모나 신생아의 합병증 여부 등이 포함된다.시험약과 임신 결과와의 관련 가능성에 대한 평가를 포함해야 하며, 임신 중에 중대한 이상반응을 경험했다면 반드시 ‘중대한 이상반응 보고 양식(SAE Report Form)’을 이용하여 보고해야 한다.

**1.2.5 과량 투여 보고를 위한 가이드라인**

임상시험약의 과량 투여는 의도적이든 실수든 ‘이상반응’으로 기록되어야 한다. 증상을 동반한 과량 투여의 경우에는 중대성의 기준을 만족하지 않더라도 이상반응/신속 보고 양식에 따라 연구자의 인지일로부터 24시간 이내에 안전성정보관리 담당자에게 보고하여야 한다. 통상적으로는 매 주기에 체표 면적 당 투여되어야 하는 용량보다 30%를 초과할 때에 과량 투여로 지칭한다.

종양

임상 연구기간 모든 형태의 악성 종양(피부 종양 포함)은 증례기록 프로그램의 이상반응 (AE; Adverse Events) 항목에 기록되며, 중대한 이상반응(SAE)으로 간주하여 중대한 이상반응 보고 양식에 서면 기록하여 반드시 24 시간 이내에 책임연구자에게 보고하며, 책임연구자는 타 연구자 에 보고 되어야 한다. 피험자가 임상연구 치료를 조기에 중단한 후 악성 종양이 발생하는 경우에도 증례기록에 기록하여야 한다.

신체검사

스크리닝/베이스라인기간이나 연구기간 중 6회의 피험자 방문시에 전체 신체검사를 실시한다. 신장(height)은 스크리닝/베이스라인만 증례기록 프로그램에 기록된다. 여기에는 연구약과 관련된 이상반응(피부 변화, 부종, 탈모, 근육 및 골격 통증, 혈압, 선택된 일부 검사실 파라미터 등)에 대한 준 정량적 평가가 포함된다.

신체검사에 대한 정보는 연구기관의 근거 문서에 기록되어 보관되어야 한다. 임상연구 개시 이전에 나타난 유의한 사항은 증례기록 프로그램에 기록하여야 한다. 이상반응의 정의에 부합하며, 임상약 투여 이후 발생한 유의한 사항은 증례기록에 기록한다.

활력징후

혈압과 맥박은 매 측정 시 동일한 팔에서 측정하며 피험자가 최소 5분 이상 앉은 상태에서 휴식을 취한 후에 측정한다. 체중은 kg 으로 기록 한다. 결과는 증례기록에 기록한다.

검사 Lab

연구자는 연구 기간 동안 연구기관의 표준진료지침에 따라 각종 검사를 진행할 수 있다. 검사는 연구자의 판단에 따라 환자 및 이식 신장의 안전과 변동을 확인하기 위하여 시행되며, 의심되는 질환이나 이상 반응을 확진하기 위하여 진행할 수 있다. 비정상 검사 결과가 확인되면 연구자는 이상반응에 기록 보고하여야 한다.

임신검사(소변 또는 혈청)는 스크리닝/베이스라인 시점에 실시하고 (결과는 연구약 복용 전에 확인 되어야 한다) 임신검사결과음성인 경우만 연구에 등록할 수 있다. 등록된 피험자는 임상연구에 참여하는 기간 동안 임상연구계획서에서 정의하고 있는 피임법을 따라야 한다. 만약 피험자의 임신이 의심되는 경우, 소변 또는 혈청검사를 통하여 확인될 수 있다. 피험자가 연구 기간 중 임신이 확인되면 그 사실을 인지한 연구자는 중대한 이상반응 보고 양식에 기록하여 반드시 24시간 이내에 책임연구자에게 보고하고, 책임연구자는 타 연구자에게 보고 한다. 자연 유산이나 인공 유산, 출생에 관한 상세 내용, 출생 결손(birth defect)이나 선천성 이상의 존재 유무 등의 결과를 판정하기 위해 임신 사례를 추적 조사하며 별도의 서면 증례기록지에 기재한다. 임상연구 치료를 시작한 후 임신이 됐을 가능성이 있는 모든 여성 피험자는 임상연구를 중단하고 지역의 표준 치료법으로 전환 한다.

시험기간동안기관의지역실험실에서아래지정된대로임상적실험실및진단적데이터를분석한다.

검사주기별로방문일정 및 평가항목을 참고하여 다음의변수를검사한다.

- 혈액학 (Hematology): 적혈구, 헤모글로빈, 헤마토크릿, 혈소판,백혈구및 분획백혈구 비율 (percentage of differential counts).
- 혈액화학 (Chemistry): 나트륨, 칼륨, 칼슘, 마그네슘, 인산, 요소(BUN), 크레아티닌, 혈당, 요산, AST,ALT, alkaline phosphatase, 알부민, 빌리루빈, 콜레스테롤, HLD, LDL, Triglyceride
- 혈청바이러스검사: B형간염(HbsAg), C형간염, HIV, CMV.
- 소변검사: 표준뇨분석 및 단회뇨PCR (Protein/Creatinine Ratio)
- 임신검사: 소변 또는 혈청 B-hCG (가임기여성)

모든피험자는연구등록 이전에 B형간염(HBsAg), C형간염, HIV, CMV 검사를실시한다. 임상적으로 변동이 의심되는 경우를 제외하고, 피험자 등록 전12개월 이내에 실시한 검사 결과로 판단하여 피험자 등록을 진행할 수 있다. B형간염표면항원또는 HIV 양성피험자는 연구에서제외된다. .

Tolerability

각 요법의 내약성 또는 용인성은 이상반응으로 인해 연구약의 감량이나 중단이 필요한 경우의 피험자 수를 백분율로 평가한다.

1. ***데이터 관리***

## *15.1 연구 기관 모니터링 (Monitoring)*

본 연구는 따로 모니터요원이 존재하지 않는다. 다만 연구진들이 정기적으로 피험자 기록의 완전성, 증례기록 프로그램의 정확성, 임상연구 계획서 및 등록 진행 현황 등을 확인하고 연구 과정이 규정에 맞게 시행되고 있는지 검토한다.

연구자는 인구학적 자료 및 의학적 정보, 실험실 데이터, 다른 검사와 평가 결과가 포함된 근거문서를 임상연구 참여 피험자 별로 유지한다. 증례기록 프로그램에 포함된 모든 정보는 피험자 파일의 근거문서 상으로 추적 가능하도록 한다. 연구자는 피험자가 서명한 피험자 동의서 원본을 보관 하며, 서명된 동의서의 사본은 피험자에게 제공한다.

또한 피험자 동의서의 존재, 선정/제외 기준의 준수, 중대한 이상반응의 기록, 모든 유효성 결과변수 및 안전성 결과변수로 이용될 자료 기록의 표준적 검증을 본원 내부점검을 통해 시행한다. 근거자료와 증례기록 프로그램의 일관성에 관한 추가 확인이 수행될 수 있다. 근거문서 상의 피험자의 신원에 대한 어떠한 정보도 외부로 노출되지 않는다.

수집된 자료는 잠금 장치에 보관하고 접근이 제한된 컴퓨터에 저장한다. 또한 증례 기록서 접근은 암호로 제한하고 접근 가능자는 연구책임자 및 공동연구자로 제한한다.

## *15.2 증례기록 및 데이터베이스 관리*

*연구기관 담당자가* 작성한 증례기록 프로그램의 완전성과 정확성을 참여 연구진들이 검토하여 필요한 수정 및 데이터 추가를 시행한다. 명백한 오류는 데이터 관리자에 의해 수정이 되며, 확인이 요구되는 데이터에 대해서는 연구기관에 데이터 확인 요청서 (Data clarification form: DCF)를 발행한다. 연구기관 담당자는 해당 데이터를 수정하여 데이터 담당자에게 발송한다.

데이터 처리 품질 관리(data management quality control procedure)절차가 완료되면 데이터를 동결한다.

연구대상자의 신상을 보호하기 위해 독립된 공간에서 면담, 설명, 동의 취득하고 연구 대상자 식별정보를 익명화 한다. 수집된 자료는 잠금 장치에 보관, 접근이 제한된 컴퓨터에 저장, 증례 기록서 접근 암호제한, 접근 가능자 (연구책임자, 공동연구자) 제한 등의 조치를 한다.

1. 자료 및 통계분석

모든 통계 분석은 PI가 수행한다. 최종 분석은 연구 대상 마지막 피험자가 연구 종료 되는 시점에서 6개월 이내에 실시한다.

분석대상 집단

모든 분석 집단은 Full analysis set(FAS), PP(per protocol) 군 모두를 분석한다

- 1차 변수의 분석을 위하여 Full analysis set(FAS) 집단을 이용한다. 이 집단은 유효성 분석과 안정성 분석을 위하여 피험자 동의서를 작성한 후 연구약을 투여 받은 모든 피험자를 대상으로 구성된다. 시험 계획서 위반, 연구약에 대한 순응도 또는 시험의 조기 중단과는 관련이 없다. 연구약 투여 전 시점에서 동의 철회 등의 이유로 종기 종료된 환자들은 제외한 후 분석한다.
- 보조적인 목적으로 Per-protocol(PP)집단을 대상으로 분석을 실시하여, 결측 자료 및 시험 계획서 위반자의 영향을 조사한다. 이러한 방식에서도 FAS 집단에서 제외시킨 동의 철회 등의 이유로 종기 종료된 환자들은 제외하고 분석한다. PP군과 FAS군의 차이가 FAS 군의 환자의 10% 미만일 경우 분석을 실시하지 않는다.
- 안정성 분석은 치료된 대로의 모든 피험자 All patients as treated(APaT)집단으로 구성된다. 이 집단 역시 연구약을 투여 받지 않은군은 제외된다.

치료 순응도

병용 면역억제제, 기타 병용약, 치료약등은 종류별로 하루 평균 복용량을 방문별로 요약한다.

**시험약물**

시험약물은 Thymoglobuline®이며다른면역억제제 이외의 처방구성약물은병용약물로간주한다.

Thymoglobuline®시험약물의 총 투여 용량 (mg/kg) 을요약한다. (체중은 screening 당시의 건체중을 기준으로 한다).계획서에준한(PP) 지침상의안전성의사유로인한용량감량(일시적투여중단포함)의빈도및평균용량을정리한다. 용량조정(일시적투여중단포함)의사유를도수분포로정리한다. 영구적치료중단을빈도별로분석한다. 이러한분석은 치료 의향(ITT; Intention-to-treat) 집단에대하여 실시한다.

**병용 면역억제제**

피험자에게 투여된 모든 면역억제제를 치료군 및시험기간별로요약하고도수분포표로정리한다. 모든면역억제제 약물은 CNI(Tacrolimus/Cyclosporin), 스테로이드, Antimetabolite(MMF 또는 Myfortic®) 그리고 기타 면역억제제를개별적으로요약한다.

순응도는처방된용량의 80%에서 120% 사이로피험자에게투여되었을때로정의된다.

**병용약제**

병용약물의모든분석결과는시기별로정리한다.

**유효성 평가**

유효성 평가 분석의 일차목적으로 다음 사항을 비교 검증한다

- 신장 이식 수술후12개월까지 생검으로 확인된 급성 세포성 거부반응 (BCAR; Biopsy-Confirmed Acute Rejection) 의 발생빈도
- 신장 이식 수술후12개월까지 모든 거부반응의(overall rejection) 발생빈도
- 신장이식 수술 후 12개월 까지의 유효성 실패(steroid re-usage, 이식편 소실율, 사망 그리고 추적조사 실패)의 발생빈도
- 신장 이식 수술후12개월까지 피험자 및 이식 신장 생존율
- 신장 이식 수술후12개월 시점의 예측 계산된 사구체 여과율 : MDRD (Modification of Diet in Renal Disease)

**안전성평가**

안전성 평가 분석의 일차목적으로 다음 사항을 비교 검증한다

- 신장 이식 수술후 각 시기의 빈혈, 백혈구감소증, 혈소판감소증의빈도빈도
- 신장 이식 수술후면역억제제에 따른 1개월, 12개월시점의 새로이 발현된 새로이 발현된 당뇨 및 당 대사 장애의 빈도를 비교한다
- 약물치료가 필요한감염의빈도와중증도
- 신장 이식 수술후 12개월까지 BK virus 및 CMV virus의 발현 빈도
- 심혈관계사건(Acute Myocardial Infarct, Unstable Angina Pectoris, Heart Failure, 뇌졸중).
- 이상반응 및 중대한 이상반응의 빈도와 중증도
- 시험약 중단을야기하는이상반응의빈도

1. 참고문헌

1. Gurk-Turner C. et al. Transplantation 2008;85: 1425–1430

2. Hrick DE et al. American Journal of Transplantation 2002; 2: 19-24

3. Martin ST et al. Pharmacotherapy 2011; 31(6): 566-573

4. Klem P et al. (Transplantation 2009;88: 891–896)

5.Kramer BK et al. American Journal of Transplantation 2010; 10: 2632–2643

6. Veenstra DL et al. Am J Kidney Dis. 1999 May;33(5):829-39.

7. Pascual J etal.Cochrane Database of Systematic Reviews 2009, Issue 1

8. Birkeland SA. [Transplantation.](http://www.ncbi.nlm.nih.gov/pubmed/9825819)1998 Nov 15;66(9):1207-10

9. Birkeland SA. Transplantation. 2001 Apr 27;71(8):1089-90.

10. Matas AJ. et al. Am J Transplant. 2005 Oct;5(10):2473-8.

11. Woodle ES. Et al. Ann Surg2008;248: 564–577)

12. Woodle ES. Et al. Clin Transplant 2010: 24: 73–83

13.Hardinger KL et al. J Transplantation 2010: 1-8

14. Stratta RJ et al. Transplantation Proceedings 2005: 37, 3546–3548

15. Brennan DC et al. N Engl J Med 2006;355:1967-77.

17. Meijer E. et al. Biol Blood Marrow Transplant. 2009 Nov;15(11):1422-30

18. Hamadani M et al. Biol Blood Marrow Transplant. 2009 Nov;15(11):1422-30

# 부록 2. 임상 연구 진행에 따른 책임과 권한

**관련 법규 및 윤리 원칙 준수**

본 임상연구는 법규 및 헬싱키선언에 기술된 윤리 원칙을 준수하고, 연구에 관련된 계획 및 예산 등 모든 자료가 연구기관의 임상연구심의위원회(IRB;institutional review board) 검토 승인 하에 진행된다. 피험자의 안전과 관련된 사항이 발생하는 경우 모든 정보는 *CRO (Clinical Research Organization)을* 통하여 수집되어 각 연구 기관에게 전달되며 연구 책임자가 연구 진행 여부를 결정할 수 있도록 한다.

**연구자 및 임상연구심의위원회(IRB;institutional review board)책임**

임상연구 개시 전에 임상연구계획서와 제안된 피험자 동의서를 적절하게 구성된 임상연구심의위원회(IRB;institutional review board)에 제출하여 검토 및 승인을 받는다. 임상연구계획서와 피험자 동의서가 승인되면 서명 및 기일이 기록된 임상연구심의위원회(IRB;institutional review board)승인 진술서를 임상연구 개시 이전에 확인한다. 연구자는 임상연구 개시 이전에 계획서의 서명페이지에 서명하여 이들 문서 및 계획서에 기술된 모든 지시사항과 절차에 따라 임상연구를 시행하고 모니터요원 및 관련당국에 모든 관련 정보와 기록을 열람할 수 있도록 동의한다.

**피험자 동의**

선정기준에 적합한 환자에 한하여 (법률 또는 규제로 요구되는 경우, 제3자 입회 하에) 임상연구심의위원회(IRB;institutional review board)승인을 받은 피험자 동의서에 서명하여 제출하거나, 본인이 직접 동의할 수 없을 경우, 환자의 위임을 받거나 법률상 유효한 대리인의 동의를 얻은 후 임상연구에 등록되어 참여할 수 있다. 모든 임상연구 관련 절차 (연구계획서에 기술된 모든 절차) 시행 이전에 피험자 동의 절차가 선행된다.

관련 규정을 준수하고 본 임상연구에 적절한 것으로 고려되는 제안된 피험자 동의서가 부록에 첨부된다. 연구자가 제시한 모든 동의서 변경사항들은 임상연구심의위원회(IRB;institutional review board)제출 전에 연구의뢰자의 동의를 얻으며, 임상연구심의위원회(IRB;institutional review board)승인을 받은 후에 승인된 동의서 사본을 모니터요원에게 제출한다.

**임상연구계획서 변경**

계획서의 모든 변경 및 추가는 연구의뢰자, 보건당국 (필요한 경우에) 및 임상연구심의위원회(IRB;institutional review board)승인을 받은 서면 변경계획서 형태로 진행된다. 변경사항이 피험자의 안전에 관계된 경우 임상연구심사위원회(IRB; institutional review board)승인 이전에 시행될 수 있다. 본 임상연구의 계획서 위반의 경우라도 참여하는 모든 피험자의 안전을 가장 우선하여 조치가 즉각적으로 (임상연구심의위원회(IRB;institutional review board)승인 이전) 진행될 수 있으며, 이러한 경우 연구 의뢰자에 그 조치 내용과 이유를 통보하고 연구기관 임상연구심의위원회(IRB;institutional review board)에도 근무일 10일 이내에 통보한다.

**임상 연구 중단**

연구자 및 연구 sponsor는 임상연구 계약에 명시된 상황에서 본 임상연구를 중단할 수 있는 권한을 유지한다. 연구가 중단되는 경우 피험자로 등록되어 있는 환자는 각 연구 기관의 표준 치료로 치료하며, 중단 시점 이전까지의 연구 결과를 취합하여 분석할 수 있다.

**결과의 출판 및 발표**

임상연구sponsor는연구결과를기초로한모든계획된발표 (communications, presentations, broadcasts 등) 및출판논문 (manuscripts)을검토할수있다. 그러나임상연구 sponsor는연구자에의한임상연구결과의출판또는기타다른유포를제한하거나방해할수없으며, 연구sponsor는연구결과의발표및출판을지원한다.

**문서의보관**

임상연구 관련문서 및 매체에 저장된 자료는 연구기관 내에서 최소 연구 종료 후 3년간 보존된다.

# 부록 3. 피해자 보상에 대한 규약

**목적**

임상연구 책임자는 모든 임상연구(이하 “임상연구”이라 약칭함)의 실시과정에서 발생하는 임상연구 피험자 (이하 “피험자”라 약칭함)의 신체 손상 등을 보상하기 위하여 약사법 시행규칙 제 31조에 따라 본 규약을 규정합니다

**보상원칙**

임상 연구 sponsor, 연구 책임자, 연구 담당자는 피험자가 임상연구에 관계된 어떠한 불이익이라도 받지 않도록 관계법규와 규범, 상호 합의한 임상연구계획서의 내용을 충실히 준수하는 데 최선을 다해야 합니다. 이러한 노력에도 불구하고 임상 연구 도중 임상 연구 방법 또는 약제간에 인과 관계가 있다고 판단되는 (임상연구용 의약품과 관련이 없음을 입증하지 못한 경우를 포함), 유해하고 의도되지 않은 반응에 의한 다음과 같은 피험자의 신체상의 손해가 발생할 경우에 연구자는 본 규약상의 보상평가기준에 따라 임상 연구 전반에 대한 임상연구배상보험을 통해 보험 혹은 정부 프로그램, 혹은 다른 제3자가 지불하지 않는 치료비용에 대해 합리적인 치료비 또는 보상금을 지급합니다.

(1) 입원 또는 입원 기간의 연장이 필요한 경우; 또는

(2) 지속적이며 의미 있는 불구나 기능 저하를 초래하는 경우; 또는

(3) 선천적 기형 또는 이상을 초래하는 경우; 또는

(4) 사망을 초래하거나 생명을 위협하는 경우

**보상요건**

임상연구 책임자는 임상연구 기간동안 피험자에게 발생하는 신체상의 피해에 대해 다음 요건을 모두 충족할 경우 책임을 부담합니다.

(1) 합의된 연구계획서에 따라 연구가 진행되는 과정 중 처방된 ATG 으로 인하여 발생한 신체상의 손해의 경우:

(2) 피험자가 임상연구계획서의 제반 내용을 준수한 경우;

(3) 피험자의 명백한 과실이나 의무태만에 기인하지 아니한 경우;

(4) 피험자가 연구책임자 또는 연구담당자의 제반 지시사항을 모두 준수하였을 경우;

(5) 피험자가 당해 신체상의 손상으로 인한 손해의 발생을 최소화하기 위하여 필요한 조치를 취하였을 경우

**보상 범위 및 대상 제외 사유**

전 보상요건에도 불구하고, 다음의 각 경우에는 본 보상규약에 따른 보상 범위에서 제외됩니다.

(1) 임상연구 과정 중 의약품으로부터 기대된 효과, 효능의 불충분으로 인한 손상 (피험자의 기왕증의 진행 및 악화로 인한 경우를 포함함); 또는

(2) 피험자의 부주의로 인하여 발생한 손상

또한 다음의 경우는 피험자가 보상 대상에서 제외됩니다.

(1) 임상연구계획서를 준수하지 않았거나 연구자의 지시를 어긴 경우 또는 연구자가 제공하지 않은 임상연구용 의약품으로 발생한 부작용에 의한 손상; 또는

(2) 질병의 치료 경과 중 발생하는 통상적 합병증에 의한 손상; 또는

(3) 임상연구용 의약품 적응증에 대해 유효한 결과가 나타나지 않는데 대한 보상; 또는

(4) 서로 합의한 임상연구계획서 또는 계약서, 설명문, 동의서 등 합의된 내용에서 이탈함으로써 야기된 손상; 또는

(5) 피험자 또는 보호자의 부주의로 인하여 초래된 손상

**보상 평가 기준**

(1) 예상된 이상반응에 대하여 당사자들간에 미리 합의한 보상액 또는 조치가 있는 경우, 당해 기준에 따라 이를 보상합니다.

(2) 그 외의 경우에는 신체손상의 본질, 정도, 성격, 기간, 지속성, 유사사례 등을 종합적으로 고려하여 적절한 액수에 따라 이를 보상합니다.

(3) 당사자들간에 전항의 합의가 이루어지지 아니한 경우에는 양자가 수용할 수 있는 전문가로부터 자문을 구하여 해결하도록 하고 이 경우에도 합의가 이루어지지 아니한 경우에는 법원의 판결 및 이에 준하는 결정의 확정내용에 따라 보상합니다.

**보상절차**

(1) 본 보상규약에 따른 신체상의 손상을 입은 피험자는 임상연구의 연구책임자나 연구기관에 먼저 필요한 의료 조치를 요청하여야 합니다.

(2) 연구책임자나 연구기관의 조치에도 불구하고 신체상의 손상이 완치되지 아니한 피험자는 임상연구 책임자에 대하여 이에 대한 보상을 요청할 수 있습니다.

(3) 임상연구 책임자는 위 보상 요청을 받은 후 14일 이내 보상 대상 해당여부 및 보상기준에 대한 조사를 마치고 이에 관한 내용을 피험자에게 통보합니다.

(4) 피험자는 위 통보내용에 대하여 이의가 있는 경우, 위 통보를 받은 날로부터 5 영업일 이내에 이에 대한 이의내용을 임상연구 책임자에게 통보하여야 합니다.

(5) 피험자가 제(3)항의 통보를 받고도 이에 대한 이의를 통보하지 아니한 경우, 양 당사자는 위 통보내용에 따른 보상에 합의한 것으로 양해합니다.

(6) 피험자가 제(4)항의 규정에 따라 이의를 통보한 경우, 임상연구 책임자는 피험자에게 위 보상대상 해당여부 및 보상기준에 관하여 판단할 객관적인 전문가를 복수로 추천하고, 피험자가 위 추천일로부터 3 영업일 내에 추천인 중 1명을 지명합니다 (피험자가 지명하지 않을 경우 임상연구 책임자가 임의 택일합니다).

(7) 피험자와 제(6)항의 규정에 따라 선택된 전문가의 자문 결과에 합의가 이루어지지 않는 경우 법원의 판결 및 이에 준하는 결정의 확정내용에 따라 보상합니다.

**적용범위**

(1) 본 보상규약은 임상연구 책임자가 시행하는 모든 임상연구에 참여하는 피험자에 대하여 임상연구 책임자와 피험자간에 다른 약정이 없는 한 그 범위 내에서 일반적으로 적용됩니다.

(2) 피험자가 임상연구에 관한 보상에 대하여 임상연구 책임자의 승인을 받지 아니하고 임상연구와 관련된 다른 제3자와 체결한 일체의 합의내용은 본 연구에 관련된 보상 규약에 대하여 효력이 없습니다.

임상연구 책임자는 앞에서 언급한 여러 제반 내용을 참고하여 환자가 임상연구에 의해 어떠한 불이익이라도 받지 않도록 주의하며, 만약 임상연구에서 문제점이 발생한 경우 연구 피험자가 입는 피해에 대해 상기 내용에 의거하여 책임질 것을 서약합니다.

임상연구 책임자 서명

#

1. 스크리닝 이전 12개월 전부터 검사결과 사용가능함. (HBsAg, HCV, HIV, CMV) [↑](#footnote-ref-1)
2. 임신검사: 소변 또는 혈청 B-hCG (가임기 여성)으로 확인하고 연구약 투여 전 확인되어야 한다. [↑](#footnote-ref-2)
3. 혈액학 (Hematology)검사: 적혈구, 헤모글로빈, 헤마토크릿, 혈소판,백혈구및 분획백혈구 비율 (percentage of differential counts). [↑](#footnote-ref-3)
4. 혈액화학 (Chemistry) 검사: 나트륨, 칼륨, 칼슘, 마그네슘, 인산, 요소(BUN), 크레아티닌, 혈당, 요산, AST, ALT, alkaline phosphatase, 알부민, 빌리루빈, 콜레스테롤, HLD, LDL, Triglyceride [↑](#footnote-ref-4)
5. 소변검사: 표준 뇨분석(Routine Urine analysis) [↑](#footnote-ref-5)
