# Supplementary material for: A prospective, randomized, non-blinded, non-inferiority pilot study to assess the effect of low-dose anti-thymocyte globulin with low-dose tacrolimus and early steroid withdrawal on clinical outcomes in non-sensitized living-donor kidney recipients
Source: PLoS One. 2023 Mar 1;18(3):e0280924. doi: 10.1371/journal.pone.0280924 (PMC9976999; doi:10.1371/journal.pone.0280924)
Supplement: S5 File — (DOCX) [file pone.0280924.s005.docx]

**Informed Consent for Clinical Research (For Living Donors)**

**Research Title: Effect of low-dose anti-thymocyte globulin with early steroid withdrawal in acute rejection and de novo donor-specific antibody formation in non-sensitized Asian living-donor kidney recipients**

**Clinical Research Director: Duck-jong Han, Department of Kidney and Pancreas Transplantation, Asan Medical Center**

**Research Institute: Asan Medical Center**

**Address: 88, Olympic-ro 43-gil, Songpa-gu, Seoul, Republic of Korea**

**1. Purpose of Research**

This research aims to investigate the clinical progress, survival rate, graft loss rate and complications after kidney transplantation in South Korea in order to increase the survival rate of patients submitted to transplantation and establish appropriate treatment guidelines.

You are asked to participate in a clinical research. In general, a clinical research is conducted for the purpose of medical advancement. The following background information is pertinent to this clinical research.

Since the first successful kidney transplantation in the United States in the 1950s, transplantation has rapidly developed globally and has become an important medical and social issue. In South Korea, more than 3,000 organ transplantations are currently being performed annually since the first successful kidney transplantation in 1969.

Transplantation requires determination of clear and objective medical criteria, such as proper donor selection and standardized patient management. Moreover, the policy on the decision processes regarding various social issues, such as refunding of related expenses, should be established in a transparent and persuasive manner. Therefore, there is a need to establish criteria that serve as the basis to objectively promote legislation, regulation, and policy establishment.

Therefore, there is an urgent need to establish a kidney transplant cohort consistent with the characteristics of the Korean population to understand the clinical course of organ transplantation; to prepare basic data related to the onset of adverse factors affecting the function of transplant organs and various complications; and to establish guidelines for the treatment for Korean patients.

Therefore, this research aims to continuously investigate data on the recovery progress, complications, and health status after kidney donation in Korean living donors. This research is a domestic, large-scale, multi-center, prospective (continued investigation over time after participation in clinical research) research funded by the Korea Centers for Disease Control and Prevention.

You could choose whether or not to participate in this clinical research. Your decision will not affect the normal kidney transplantation treatment process. Please take the time to read this informed consent. Please talk to our researchers in case of queries to make an informed decision.

**2. Expected Participation Period and Estimated Number of Total Individuals Participating in This Research**

This research is expected to begin from the time of approval by the Institutional Review Board (IRB) and will continue for about 9 years until December 2022. The total number of individuals is expected to be 9,200 pairs.

**3. Research Method and Procedure**

The following tests and procedures are required for your participation until termination of this clinical research:

Items for observation include your height, weight, history of other systemic diseases in the past or present, number of pack-years, etc.

Items for clinical tests include your renal function, viral infection status, blood type, HLA genes, donor-specific antibodies, etc.

In addition, renal function test results and postoperative complications are examined every one year after surgery.

In addition, prior to transplantation, blood and genes of patients that provided consent to such terms are collected and stored in separate storage facilities.

**4. Other Treatment Options for This Disease**

You may decide not to participate in this research. Regardless of your consent to participate in the research, the generally accepted methods of treatment for living donors in kidney transplantation apply to you and you may discuss these treatment options with your doctor.

**5. Costs, Expenses, and Compensation for Participation in Clinical Research**

Since the tests performed during the period of participation to clinical research are usually related to the treatment of living donors and are necessary for the diagnosis and treatment of patients, the expenses are incurred upon individuals. There is no financial reward for participating in this research, and there are no additional costs incurred upon individuals by participating in the study.

**6. Compensation and Treatment for Damage Related to This Research**

This is a follow-up cohort study in which you are treated according to conventional guidelines, and there is no risk of further damage due to participation in this research. A cohort study refers to a prospective follow-up investigation, which indicates that patients undergoing kidney transplantation will be recruited for follow-up investigations during the observation period of this research. However, since it is a long-term (9 years) research, we will ensure not to infringe on your personal information and privacy and take immediate action in the event of a breach. If it is determined that any infringement is related to this research, the researchers participating in the cohort study (Cohort Secretariat) will be responsible for the damage and appropriate compensation.

**7. Matters to Follow for This Research**

Be sure to follow the instructions of your attending physician to ensure successful treatment, as well as to contribute to accurate scientific research. If you experience any damage related to this research, please inform the attending physician immediately.

**8. Benefits of Participating in This Research**

There will be no difference in terms of treatment outcomes, regardless of participation in this research. Although there no direct additional benefit is expected for individuals participating in this research, the results obtained may benefit others who will be treated for chronic kidney failure in the future.

**9. Risks, Adverse Effects or Inconveniences Expected from Participation in Clinical Research**

This research is an observational study in which the effect of treatment is observed regularly by evaluating observational items or examining clinical test results; therefore, participation in this clinical research does not increase the risk compared to non-participation.

However, the following process may cause discomfort:

- A blood sample is collected once (only from patients who have provided consent to blood collection) prior to transplantation for the entire research period (9 years).

**10. Potential Risks**

This research is an observational study, and it is not conducted to test the effects of any specific drug or treatment. There is no specific risk, because the conventional guidelines for living donors in kidney transplantation will be followed. However, the usual adverse effects associated with living donors in kidney transplantation still remain, and you may discuss precautions with your attending physician. Moreover, adverse events (pain, phlebitis, etc.) may occur while drawing blood during blood sample collection.

**11. Premature Discontinuation of Clinical Research**

The clinical research director may decide to exclude you from the study in the following cases:

- Meeting the selection exclusion criteria
- Withdrawal of consent
- Transfer to another medical institute not participating in the research
- Difficulty in performing the follow-up investigation
- Other (cases for which the research doctor determines that he/she is unable to perform further clinical research due to reasons such as poor patient adherence to the instructions, or IRB decision to terminate the research, etc.)

**12. Collection and Storage of Research Samples**

Some institutes may collect approximately 8 mL of your blood prior to transplantation for this research. The sample collected for research will not be used for any other purpose, and it will be discarded after being stored for up to 10 years from the end of the research unless you specify otherwise. The sample is attributed a unique number, without personal information, such as medical record number and name. In the event of your request for discontinuation of the research or disposal of the sample, the sample will be discarded with any identifiable label removed.

**13. Confidentiality of Identity**

Your record containing personally identifiable information will be kept confidential and will not be subjected to public disclosure. However, your record may be viewed by a monitor agency, an investigator or a review committee to verify the reliability of the research procedure and data in accordance to relevant laws or regulations. Even in this case, we will try to keep it as confidential as possible. By signing this consent form, you allow direct access to these materials, and your identity will be kept confidential if the results of the research are published. Your personal information is collected due to participation in this clinical research, but this information is not directly used or required for this research; it is used solely for the purpose of linking your clinical data obtained as a result of the clinical research. Therefore, the collected information is used up to 10 years from the end of the study and is properly managed in accordance with the Personal Information Protection Act. In addition, if you transfer to another participating institute, you may continue participating in the research by providing another consent form with your information including your name, date of birth and date of transplantation to the research team at the receiving institute for collection of the follow-up data.

**14. Consent and Withdrawal of Consent to Participate in Research on a Voluntary Basis**

You may participate in this study voluntarily. Even if you do not agree to participate, you will not be penalized for further treatment. You may freely withdraw from participating in this study at any time during the research period, and this will result in no disadvantages for your treatment.

**15. New Information that May Influence Your Willingness to Participate**

Your participation in this study is entirely voluntary. When new information is collected that could affect your willingness to continue to participate in this research, we will notify you or your legal representative in a timely manner.

**16. Use as Future Research Materials or Sample Materials**

After the end of this research, patient data may be used for other related studies. However, the existing data collected at the beginning of the study will be used with no additional blood or urine sample collection required.

**17. Provision of Information on Rights and Interests of Participating Individuals**

You have the right to ask questions at any time about any known or potential risks associated with this research.

If you have any questions regarding this research, you may contact the Research Ethics Officer at the Human Research Protection Center of Asan Medical Center Institutional Review Board (IRB), who is responsible for protecting the rights, safety and welfare of participating individuals. You may also contact the researchers:

Clinical Research Director

Duck-jong Han, Professor, Department of Kidney and Pancreas Transplantation, Asan Medical Center

02-3010-5693

Clinical Research Nurse

Joo-hee Jung, Advanced Practice Nurse, Department of Kidney and Pancreas Transplantation, Asan Medical Center

010-4895–0727

If you have any questions about your welfare and rights as a participant in clinical research study, or if you would like to consult with someone who is not directly related to the research, please contact the numbers below.

Human Research Protection Center 02-3010-7161

Institutional Review Board 02-3010-7166

**Consent for Clinical Research (For Living Donors)**

**Research Title: Establishment and Operation of a Cohort for Organ Transplantation; a Cohort Study on Kidney Transplantation**

**Clinical Research Director: Duck-jong Han, Department of Kidney and Pancreas Transplantation, Asan Medical Center**

**Research Institute: Asan Medical Center**

**Address: 88, Olympic-ro 43-gil, Songpa-gu, Seoul, Republic of Korea**

1. I have read and understood all information provided prior to this clinical research, and received satisfactory answers to all my questions.
2. I intend to voluntarily participate in this clinical research, and I understand that I will be provided with a copy of this consent form after providing my consent.
3. I agree to the collection of my medical records and clinical information by gathering and providing personal information such as my name under strict confidentiality while participating in this clinical research.
4. If I transfer to another participating institute, I agree to provide information such as my name, date of birth and date of transplantation to the research team at the receiving institute.
5. I agree to the storage and disposal of my blood samples collected during the clinical research period for the period agreed upon in the human material research agreement.
6. I agree that my clinical information collected during the clinical research period may be transferred and stored at the Korea Centers for Disease Control and Prevention of the Ministry of Health and Welfare, and then used secondarily (provided as non-personally identifiable information to researchers who have not participated in this study) for a research to overcome the disease for public benefit.
7. I agree that my clinical information collected during the clinical research period may be used secondarily (provided as non-personally identifiable information to researchers who have not participated in this study) for an international cooperative research in the future for public benefit and overcoming disease.

※ The collected information will be provided after deliberation in accordance with the regulations of the Korea Centers for Disease Control and Prevention, and it is strictly managed in accordance with the “Bioethics and Safety Act.” Under national law, if you are a minor under the age of 19, both you and your legal representative must sign and date the consent form.

| Research Subject | Name: | Signature: | Date of Signature: |
| --- | --- | --- | --- |
| Legal Representative  (If required) | Name: | Signature: | Date of Signature: |
|  | Relationship: | Cause: | |
| Witness  (If required) | Name: | Signature: | Date of Signature: |
|  | Relationship: | Cause: | |
| I confirm that I have provided the above applicant a detailed explanation of the purpose, testing process and risk factors of this clinical research. | | | |
| Researcher | Name: | Signature: | Date of Signature: |
