# Supplementary figures and images for: A prospective, randomized, non-blinded, non-inferiority pilot study to assess the effect of low-dose anti-thymocyte globulin with low-dose tacrolimus and early steroid withdrawal on clinical outcomes in non-sensitized living-donor kidney recipients
Source: PLoS One. 2023 Mar 1;18(3):e0280924. doi: 10.1371/journal.pone.0280924 (PMC9976999; doi:10.1371/journal.pone.0280924)

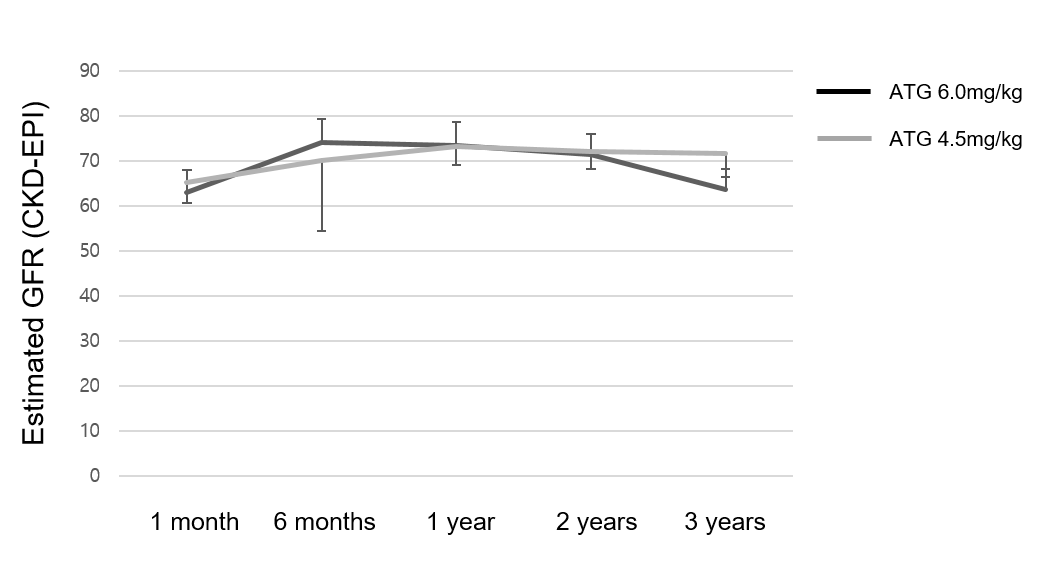

Supplement: S6 File — ATG, anti-thymocyte globulin; GFR, glomerular filtration rate. (TIF) [file pone.0280924.s006.tif]

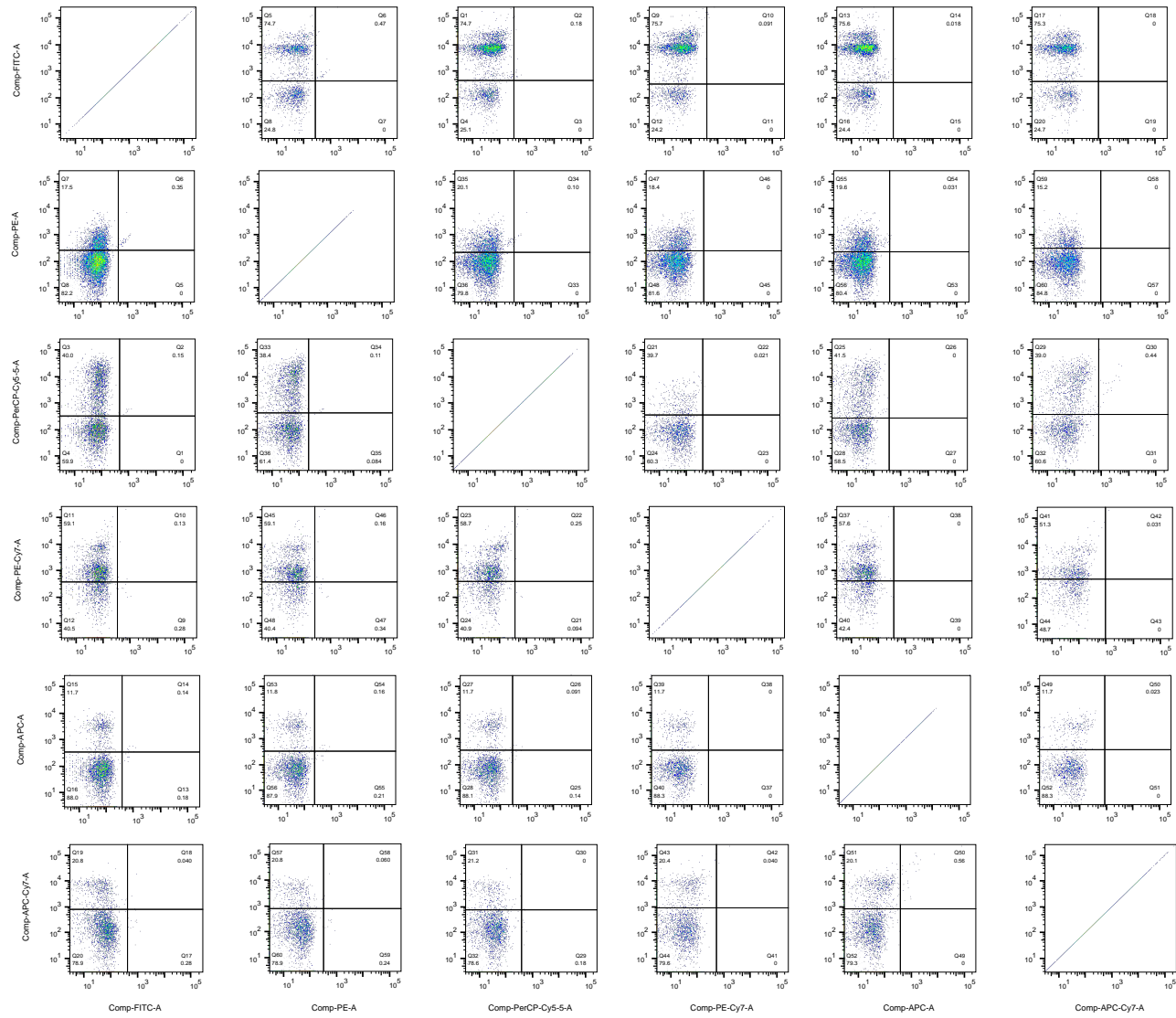

Supplement: S7 File — (PDF) [file pone.0280924.s007.pdf]
