# Supplementary material for: A prospective, randomized, non-blinded, non-inferiority pilot study to assess the effect of low-dose anti-thymocyte globulin with low-dose tacrolimus and early steroid withdrawal on clinical outcomes in non-sensitized living-donor kidney recipients
Source: PLoS One. 2023 Mar 1;18(3):e0280924. doi: 10.1371/journal.pone.0280924 (PMC9976999; doi:10.1371/journal.pone.0280924)
Supplement: S8 File — (DOCX) [file pone.0280924.s008.docx]

The clinical trial protocol

**Title: A prospective randomized controlled study to evaluate feasibility and safety of early steroid withdrawal after 6mg/kg vs 4.5mg/kg Thymoglobulin^®^ induction therapy in living donor kidney transplantation.**

Protocol No. 2016V5.3

Phase Phase IV IIT (Investigator initiated Trial)

Principle Investigator Duck Jong Han

Collaborator Young Hoon Kim

Collaborator Sung Shin

Collaborator Youngmin Ko

Collaborator Hyunwook Kwon

Collaborator Yu-Mee Wee

Collaborator Joo Hee Jung

**Summary of the protocol**

| Name of the clinical trial | A prospective randomized controlled study to evaluate feasibility and safety of early steroid withdrawal after 6mg/kg vs 4.5mg/kg Thymoglobulin^®^ induction therapy in kidney transplantation |
| --- | --- |
| Purpose of the clinical trial | The aim of this study was to compare the efficacies of 4.5 mg/kg ATG and 6.0 mg/kg ATG in non-sensitized living-donor kidney recipients with early steroid withdrawal in an Asian population, and to investigate the immunologic profiles thereof during follow-up. |
| Design of the clinical trial | This was a prospective, open-label, randomized, non-inferiority pilot study in living- donor kidney transplant recipients at Asan Medical Center (Seoul, South Korea) |
| Sample size | 154 |
| Study period | Recruitment of participants : 24 months, Follow up : 12 months |
| Drug | Thymoglobuline® (Rabbit Anti-thymocyte globulin) |
| Other immunosuppressants | Tacrolimus, Corticosteroids, Mycophenolate mofetil (or EC-MPS) |
| Inclusion criteria | • Patients above 18 and below 70 years of age who were prepared for living donor kidney transplantation  • Patients who have the ability and will to consent to participation in this study. |
| Exclusion criteria | • A multi-organ transplant recipient  • A panel-reactive antibody of more than 20% or pre-transplant donor-specific antibody  • A recipient who prepared for ABO- or HLA-incompatible kidney transplantation  • A recipient who had a kidney allograft from an HLA-identical donor  • A recipient who had re-transplantation  • A recipient who had a known contraindication to the administration of ATG. |
| Methods | Patients are randomly assigned to receive either 4.5 mg/kg or 6.0 mg/kg of ATG; all patients have corticosteroid withdrawal within 7 days. |
| End points | The aim of this study was to compare the efficacies of 4.5 mg/kg ATG and 6.0 mg/kg ATG in non-sensitized living-donor kidney recipients with early steroid withdrawal in an Asian population, and to investigate the immunologic profiles thereof during follow-up.  **The primary efficacy end point**  - A composite of biopsy-proven acute rejection (BPAR), de novo donor-specific antibody formation, and graft failure  **The secondary efficacy end point**  - Renal function determined by eGFR (CKD-EPI) at one and six months post-transplant and one and two years post-transplant.  **The safety end points**  - Infection, leukopenia, thrombocytopenia, and malignancy |

1. **Research background**

In order to prevent adverse rejection episodes after kidney transplantation, immunosuppressants are essential components of the treatment and they include CNI agents such as Tacrolimus or Cyclosporin, steroid agents, and antimetabolites such as MMF (Mycophenolate mofetil). CNI agents can be very effective but nephrotoxicity is possible during the period of use and steroid agents have some side-effects.

Another way to prevent acute rejection is to use an induction therapy. Currently, induction therapy agents used in Korea include Simulect, which inhibits IL-2 receptors, and Thymoglobuline®, which is a type of polyclonal Ab. Advantages of induction therapy in kidney transplantation include reducing the frequency of acute rejections and reducing side effects by reducing the dose of immunosuppressive drugs (especially CNI agents) used after surgery.

In addition, many attempts have been made to reduce dose of steroids after transplantation using Thymoglobuline®, a potent induction therapy agent, and good results have been reported. However, it is unknown whether the dose can be applied to Koreans as most of the studies were conducted in the foreign countries.

Therefore, the aim of this study is to compare and evaluate the efficacy and safety of different doses of Thymoglobuline® during steroid discontinuation therapy through induction therapy by Thymoglobuline® induction therapy in kidney transplants.

1. **Research purpose**

Primary goal

-Rate of combined events of first histologically confirmed acute rejection, delayed graft function, graft failure, and death-censored survival rates up to 12 months after transplantation

Secondary goals

- Histological extent of rejection (according to Banff criteria)

- Overall rejection rate up to 12 months

- Rate of steroid-free periods at 12 months

-Renal function (GFR) at 12 months

- Observation of changes in immune cells in blood, various proteins and cytokines in urinary samples for 12 months

-CMV, BK virus expression frequency

-Frequency and severity of infections requiring medical interventions

-Hematologic AE frequency (anemia, Leukopenia, thrombocytopenia)

-NODM incidence rate.

1. **Research design**

3.1 Subject Participation Period

Subjects enrolled in the study are required to be observed for up to 12 months (365 days ± 30 days) after kidney transplant surgery.

3.2 Clinical study period

36 months after IRB study approval (subject recruitment period: 24 months, treatment period: 12 months)

3.3 Definition and Activities of the Clinical Study Period

Screening/Baseline: This is defined as the time from the time the subject consented to the clinical research and the final subject suitability evaluation and baseline examination were completed to the time immediately after the kidney transplant surgery was performed. All procedures must be performed only after the subject signed the agreement form. The researcher makes a decision on whether or not to include the patient in the clinical study according to the selection/exclusion criteria. The final evaluation of suitability must be conducted as soon as possible after the patient has signed the consent form, and if 12 weeks have elapsed from the time of consent to the clinical research, a new consent procedure is required. Baseline examination can be selected among the examinations within 12 weeks before renal transplantation, unless there was a change in clinical course. (PRA test up to 1 year before the transplantation can be selected)

Evaluation lists: subject consent form, selection/exclusion criteria confirmation, recipient background information, donor background information, kidney transplant surgery information, past history information, vital signs, physical examination, concomitant drug administration record, serum viral test, pregnancy test, hematology test, blood chemistry test, lipid test, glycated hemoglobin (HbA1c), PRA test

- Blood and urine collection for clinical research to check the degree of changes in immune cell concentration, protein, and cytokines according to the administration of clinical research drugs: Blood (10 cc in a Heparin bottle) and urine (20 cc in a sterile bottle) for clinical research are collected to check the concentration and changes in characteristics of immune cells (T cells, B cells, NK cells) according to Thymoglobulin administration before and 1 week, 1 month, 3 months, 6 months, and 12 months after surgery and requested to Asan Life Sciences Research Institute for analysis. After analysis for this study, both blood and urine are discarded.

**Visit 1 (POD 14 days, ± 7 days):** Defined as the period from the subject's kidney transplantation to the first visit to the out-patient clinic between 14th ± 7th postoperative days when immunosuppressive therapy and clinical examinations are evaluated.

Evaluation lists: *Vital signs, physical examination, concomitant drug administration record, hematology test, blood chemistry test, lipid test, urine test, immunosuppressant administration record, oral hypoglycemic agent or insulin administration record, CNI trough level, graft rejection, renal biopsy record, (serious) adverse events, infection, post-transplant dialysis, graft loss*

**Visit 2 (POD 1 month, ± 7 days):** Defined as the period from the subject's kidney transplantation to the visit to the out-patient clinic between 30th ± postoperative days when immunosuppressive therapy and clinical examinations are evaluated.

Evaluation lists: *Vital signs, physical examination, concomitant drug administration record, hematology test, blood chemistry test, lipid test, urine test, immunosuppressant administration record, oral hypoglycemic agent or insulin administration record, CNI trough level, graft rejection, renal biopsy record , (serious) adverse events, infection, post-transplant dialysis, graft loss*

**Visit 3 (POD 3 months, ± 14 days):** Defined as the period from the Visit 2 (1 month, ± 7 days) to the visit 3 months ± 14 days after transplant surgery when immunosuppressive therapy and clinical examinations are evaluated.

Evaluation lists: *Vital signs, physical examination, concomitant drug administration record, hematology test, blood chemistry test, lipid test, urine test, glycated hemoglobin (HbA1c), immunosuppressant administration record, oral hypoglycemic agent or insulin administration record, CNI trough level, graft Rejection, renal biopsy records, (serious) adverse events, infections, post-transplant dialysis, graft loss*

**Visit 4 (POD 6 months ± 14 days):** Defined as the period from Visit 3 (3 months ± 14 days) to the visit 6 months ± 14 days after transplant surgery.

Evaluation lists: *Vital signs, physical examination, concomitant drug administration record, hematology test, blood chemistry test, lipid test, urine test, immunosuppressant administration record, oral hypoglycemic agent or insulin administration record, CNI trough level, graft rejection, renal biopsy record, (serious) adverse events, infection, post-transplant dialysis, graft loss*

**Visit 5 (POD 9 months ± 30 days**): Defined as the period from Visit 4 (6 months ± 14 days) to the visit 9 months ± 30 days after transplant surgery.

Evaluation lists: *Vital signs, physical examination, concomitant drug administration record, hematology test, blood chemistry test, lipid test, urine test, immunosuppressant administration record, oral hypoglycemic agent or insulin administration record, CNI trough level, graft rejection, renal biopsy record, (serious) adverse events, infection, post-transplant dialysis, graft loss*

**Visit 6 (POD 12 months ± 30 days):** Defined as the period from Visit 4 (6 months ± 14 days) to the visit 12 months ± 30 days after transplant surgery.

Evaluation lists: *Vital signs, physical examination, concomitant drug administration record, hematology test, blood chemistry test, lipid test, urine test, glycated hemoglobin (HbA1c), immunosuppressant administration record, oral hypoglycemic agent or insulin administration record, CNI trough level, graft Rejection, renal biopsy records, (serious) adverse events, infections, post-transplant dialysis, graft loss*

1. Subject

Selection criteria

Male or female end-stage renal failure patients aged 18 to 70 years

Patients who are to receive a kidney transplant from a non-blood related or related living donor or cadaveric donor due to brain death and cardiac death

Patients who have the ability and willingness to consent to participate in clinical research, have signed the research consent form in accordance with appropriate procedures, and can visit and participate in clinical research as planned

Exclusion criteria

Patients who are scheduled to receive transplantation of multiple organs other than kidneys or two kidneys, or who have previously received or will receive other organ transplants (pancreas, heart, lung, bone marrow, stem cell)

Patients with a PRA of 50% or more (DSA-positive patients: when single bead PRA is performed)

Patients who are to receive a kidney from ABO blood type mismatch donor or lymphocyte cross-

Patients who have been diagnosed with cancer within the last 5 years (excluding squamous cell or basal cell carcinoma skin cancer that had been treated)

Patients with active infection

Patients who will receive a transplant from a donor with the same HLA type

Patients who received a kidney transplant from a donor with a positive HIV, HBsAg or anti-HCV RNA test result, or tested positive for viral tests

Patients who have a history of severe allergy or hypersensitivity that requires acute (within the last 4 weeks) or chronic treatment to drugs used in clinical research or drugs with similar chemical structures, or if have contraindications to ATG administration

Patients who have received other clinical investigational drugs within 30 days prior to study enrollment

Female patients who are planning a pregnancy, who are pregnant and/or lactating, and who do not intend to use effective contraception during the study period

Patients with an uncontrolled disease or a medical condition that requires ongoing treatment

Patients with a history of addiction to alcohol or drugs within the last 24 weeks

Patients with the following conditions at the time of pre-kidney transplant evaluation:

- If two or more of the liver function tests (AST, ALT, ALKP, total bilirubin) values ​​are increased by more than three times the normal range

- Patients with absolute neutrophil count <1,000/mm^3^, white blood cell count <3,000/mm^3^, or platelet count <75,000/mm^3^

- If the donor is over 65 years of age

※ Matters concerning pregnancy and contraception in women of childbearing age: In the case of pre-menopausal female subjects under the age of 60, a urine or serum pregnancy test is performed at the beginning of the clinical study, and only women with negative test results ought to participate in the study. The test results should be confirmed before taking the drug for clinical trials. All women of childbearing potential should continue to use reliable methods of contraception throughout the study. Reliable methods of contraception include total abstinence or intrauterine devices or other essure, condoms, diaphragms, shields, caps, sponges, and spermicides. Neither periodic abstinence (e.g., ovulation cycles, symptom temperature, etc.) nor external ejaculation are considered reliable methods of contraception. All types of hormonal contraception are not permitted during this study because other immunosuppressants that can be used in combination may reduce the contraceptive effect of oral contraceptives. Postmenopausal women (with 12 months of natural amenorrhea) can participate without using contraception.

Basis for calculating the target number of subjects

Incidence rate of 50.4% reported in previous reports (Brennan DC, Daller JA, Lake KD, et al. N Engl J Med 2006; 355: 1967-1977) which were based on composite end point (mortality, graft failure, biopsy proven acute rejection, delayed graft function) and based on the retrospective analysis data at the institute, the incidence rate in the experimental group was expected to be at 40%. And through the calculation as a non-inferior study, the number of subjects in the control and experimental groups are expected to be 77/77, respectively.

1. Treament

5.1 Research drug

**Study drug**:

Thymoglobuline® (ATG) : Rabbit anti-thymocyte globulin

**Dosing schedule:**

Dose : 1~1.5mg/kg/dose (Total 6mg/kg vs. 4.5mg/kg)

Frequency : post-operative days 0,1,2,3,4,5

*Note: The first dosing should be initiated prior to perfusion on the day of surgery.

Weight is based on preoperative weight

- 1. Concomitant immunosuppressive agents:

CNI agent

- Use Tacrolimus.

- Start with an initial oral dose of 0.05 mg/kg BID (recommended) within 2 days before and after transplantation (from 2 days before transplantation to 2 days after transplantation) to reach the baseline blood level (C0: trough blood level). Maintain 7-10 ng/mL within 3 months after surgery, 5-8 ng/mL within 3-12 months, and 4-7 ng/mL after 12 months after surgery.

Corticosteroids (methylprednisolone / prednisone)

- For all subjects, intravenous administration of 500 mg of methyl-prednisolone on the day of kidney transplantation, 250 mg on the 1st day after surgery, 125mg on the 2nd day of surgery, 60mg on the 3rd day of surgery, 40mg on the 5th day of surgery, and 20mg on the 6th day of surgery is recommended, but it can be controlled by each center’s protocol.

- In the case of DGF, extended use is possible at the discretion of the researcher, but it must be stopped within 14 days

Antimetabolite

- MMF (Mycophenolate Mofetil) or Myfortic (enteric-coated mycophenolate sodium) is recommended

- MMF (Mycophenolate Mofetil) 500 mg - 1,000 mg BID is administered orally within 2 days before and after transplantation (from 2 days before transplantation to 2 days after transplantation), and the dose is reduced or increased depending on the clinical course of the kidney graft. Myfortic (Enteric Coated Mycophenolate Sodium) is prescribed and taken at 0.72 times the dose of mycophenolate mofetil. For example, a patient on mycophenolate mofetil 500 mg BID would switch to Myfortic® 360 mg BID.

- Anti-histamine and acetaminophen are administered 30 minutes before administration of the study drug, Thymoglobulin® (prescribed and administered according to the normal procedure of the research institution)

5.3 Treatment group assignment

In this clinical trial, test group/control group is assigned by random assignment (Block Random).

5.4 Prescription and administration of concomitant immunosuppressive drugs

The researcher prescribes an immunosuppressive drug, taking into account of additional dose requirements for possible loss or damage, reservation problems, etc. Grapefruit or grapefruit juice should be avoided to prevent changes in the bioavailability of tacrolimus.

Researchers should guide subjects to take concomitant immunosuppressive drugs exactly as prescribed and increase medication compliance by mentioning that medication compliance is essential for the safety of subjects and the effectiveness of the study.

All immunosuppressive drugs should be administered orally with the following exceptions:

a. Methylprednisolone is administered intravenously.

b. Thymoglobulin ® is administered intravenously;

c. In cases where oral administration of the drug is impossible due to gastrointestinal complications or when drug absorption is judged to be impaired, an immunosuppressant drug may temporarily be administered intravenously, and the dose of the intravenous drug is determined according to the bioavailability index. (For example, if Prograf® is to be injected intravenously, administer 1/3 of the oral dose)

Delayed graft function (DGF) is defined as requiring dialysis within the first 7 days after transplantation, in which case the dose of Tacrolimus may be reduced or temporarily discontinued.

5.5 Dose adjustment and discontinuation of clinical drugs

Patients enrolled in the study undergo a daily general blood test during the administration period of the study drug (Thymoglobulin), and adjust the dose according to the following criteria.

- Leukocytes> 3,000 cell/mm^3^: normal dose administration

2000 < leukocytes < 3,000 : 1/2 dose reduction

2,000 > Leukocytes: discontinuation of administration

- Platelets > 75,000 cell/mm^3^ : normal dose administration

75,000 > platelets > 50,000 : 1/2 dose reduction

50,000 > platelets: Discontinue administration

If the subject is enrolled in the study and needs elective surgery while taking CNI and antimetabolite, or emergency surgery is performed during the course of the study, CNI and antimetabolite can be injected intravenously, reduced or discontinued up to 14 days while maintaining the subject's study registration according to the investigator's judgment.

Moderate to severe thrombocytopenia, anemia, and leukopenia that do not respond to standard treatment due to immunosuppressive s drugs (Tacrolimus, MMF or Myfortic®, Azathioprine, etc.) specified in the study protocol and in case of other serious adverse reactions, proper and routine treatment should be initiated. If there is no response to general and conventional treatment or if the process is worsening, the immunosuppressive agent can be reduced or discontinued. If it is necessary to reduce or discontinue some of the immunosuppressants specified in the research protocol, the subject will drop out of the study and switch to the standard treatment of the reseac center.

All concomitant immunosuppressive drugs, including clinical drugs in this study, can be changed according to medical reasons and research plans.

Steroid discontinuation therapy is not performed if ATG is not sufficiently administered (Thymoglobulin ® < 4.3mg/kg) for the above reasons.

1. Concomitant Medications or Treatments

All information regarding the subject's concomitant treatment should be recorded, including the name of treatment or drug, duration of treatment, and reason for treatment. All immunosuppressive therapies (Tacrolimus, MMF or Myfortic® Corticosteroids, other antibody therapies, etc.) are recorded in the case record program, and the name of the drug, total daily dose and duration of treatment should also be recorded.

## 6.1 Essential treatment

Standard prophylaxis to prevent the onset of Pneumocystis infection pneumonia (Pneumocystis Carinii) is performed in all subjects for at least 6 months after kidney transplantation, and specific prophylaxis should follow the standard protocol of the research institute.

All CMV-negative patients receiving a kidney transplant from a cytomegalovirus (CMV)-positive donor should receive standard prophylaxis to prevent CMV infection after transplantation.

## 6.2 Recommend treatment

Treatment of hyperlipidemia and hypertension according to guidelines.

CMV treatment during CMV infection through CMV prophylaxis and CMV monitoring (CMV antigenemia) during hospitalization.

Immunosuppressant reduction treatment through BK monitoring (Blood PCR)

## 6.3 Acceptable treatment

Treatment of other preceding or emergent conditions.

Standard drug therapy during and after surgery. (Prophylactic antibiotics and fungal prophylaxis)

The use of fluconazole is permitted for the treatment of fungal infections. At this time, more attention should be paid to the change in CNI blood concentration, and a dose reduction may be required.

## 6.4 Forbidden treatment

- Investigational drugs registered and administered in other clinical studies.
- The potent cytochrome P450 inducers/inhibitors listed below are limited for the purpose of evaluating the effectiveness of the study, except when the researcher makes a clinical decision that it is absolutely necessary according to the subject's clinical course. Depending on the researcher's judgment, these drugs can be used cautiously, and when using the drug, it is necessary to adjust the dose by examining the blood concentration of Tacrolimus. If target level is not met, patients ought to be dropped out and switched to standard treatment at the research institute.

| A. Agents that cause changes in the pharmacokinetics of immunosuppressants | | |
| --- | --- | --- |
| Drugs that can potentially increase the concentration of Tracrolimus | | Drugs that can potentially decrease the concentration of Tracrolimus |
| diltiazem*  nicardipine*  verapamil*  danazol  doxycycline  clotrimazole  metoclopramide  bromocriptine | erythromycin & derivatives  clarithromycin  telithromycin  troleandomycin  ketoconazole (except topical)  itraconazole  ritonavir  indinavir | carbamazepine  phenobarbital  phenytoin  rifampin  rifabutin  rifapentine  octreotide (경구용제형만해당함) |

Cytochrome P450 inhibitors cannot be used to facilitate dose reduction of Tacrolimus.

For the prophylaxis of oral candidiasis, fluconazole cannot be administered systemically by oral or injection route.

Aminoglycosides, amphotericin B, cisplatin, or other pharmacological therapies associated with decreased renal function that have not been discontinued prior to the start of the study should be limited as much as possible.

Grapefruit or grapefruit juice should be avoided to prevent changes in the bioavailability of tacrolimus.

During immunosuppressive treatment after kidney transplantation, vaccination may generally be less effective, and the use of live or toxic vaccines is contraindicated.

Pretreatment

Investigators should make every effort to identify all relevant treatments or medications that the patient has been receiving for at least 4 weeks prior to kidney transplant surgery. All relevant information on the subject should be recorded in a case record program including the name of treatment and medication and duration of treatment.

1. Diagnosis and Treatment of Acute Rejection

Any subject suspected of having a rejection reaction (a rise in creatinine of 30% or more for no apparent reason) will undergo a kidney biopsy, kidney scan, kidney ultrasound, or other hematologic diagnostic procedure at the discretion of the clinical investigator to determine the proper management. In cases of suspected acute rejection, biopsy should be performed first before initiating treatment to confirm the diagnosis, unless biopsy is contraindicated. However, treatment may be initiated before biopsy results are available. If a kidney biopsy cannot be performed prior to initiation of treatment, a biopsy should be performed as soon as possible (within 48 hours) after initiation of treatment. Renal allograft rejection will be determined by a pathologist according to 2007 Banff criteria.

Acute rejection is treated according to the treatment policy of the research institution. For the treatment of acute rejection, use at least 500 mg/day IV methylprednisolone (other steroids are acceptable when administered at bioequivalent doses) for at least 3 doses. The first rejection reaction can be treated with steroids, and discontinuation of steroids can be attempted at the investigator's discretion.

In case of steroid-resistant rejection, anti-lymphocyte therapy can be initiated at appropriate doses for 7-14 days. However, if there is an inadequate response to steroid therapy or if initiation of anti-lymphocyte therapy is delayed by more than 14 days from biopsy-confirmed acute rejection, it is advisable to perform a biopsy again to confirm the rejection prior to initiation of anti-lymphocyte antibody therapy

If humoral rejection is confirmed, anti-lymphocyte therapy can be performed along with plasmapheresis, rituximab, and immunoglobulin injections.

If a serious infection occurs during acute rejection treatment and requires administration of antibiotics, antifungals, or antivirals, patients should receive the standard treatment at the relevant research institution.

Subjects who have a finding of Banff classification Grade III or higher (including Grade III) as a result of a biopsy of the transplanted kidney, or who have experienced steroid resistance rejection, or who have experienced rejection two or more times during the treatment period, should discontinue the clinical study and receive standard treatment.

1. Anticipated Drug Interactions

8.1. CNI drug interactions

Calcineurin inhibitors (Tacrolimus) are metabolized by the cytochrome P450 enzyme system, so substances known to inhibit these enzymes may reduce the metabolism of both drugs, resulting in increased whole blood or plasma concentrations. Conversely, drugs known to induce these enzyme systems may increase metabolism, resulting in decreased whole blood or plasma concentrations. Drugs that increase CNI concentration include calcium channel blockers (Diltiazem, Nicardipine, Verapamil), antifungals (Ketoconazole, Fluconazole, Itraconazole), antibiotics (Clarithromycin, Erythromycin), glucocorticoids, and other drugs (Allopurinol, Bromocriptine, Danazol, Metoclopramide). . Drugs that decrease CNI levels include antibiotics (Naficillin, Rifampin), antiepileptic drugs (Carbamazepine, Phenobarbital, Phenytoin), and other drugs (Octreotide, Ticlopidine, Troglitazone). When using these drugs in combination, appropriate dose adjustments should be made by reflecting the blood concentration test.

CNI drugs are nephrotoxic, and the nephrotoxicity may be aggravated by interactions with drugs. In addition, concomitant use of nonsteroidal anti-inflammatory drugs (NSAIDs), particularly in dehydrated conditions, can exacerbate renal insufficiency. Drugs that can aggravate renal insufficiency include antibiotics (Gentamycin, Tobramycin, Vancomycin, etc.), antifungals (Amphotericin B, Ketoconazole), gastrointestinal ailments (Cimetidine, Ranitidine), anticancer drugs (Melphalan), and anti-inflammatory drugs (Azapropazon, Diclofenac, Naproxen, Sulindac), etc.

Reduced excretion of prednisolone, digoxin, lovastatin, and simvastatin was observed when administered with CNI. In addition, a clear decrease in digoxin half-life was reported after CNI administration. Severe digitalis toxicity occurred within several days of initiation of tacrolimus in a few patients receiving digoxin. Because hyperkalemia may occur, caution should be exercised when administering tacrolimus with potassium-sparing diuretics.

During CNI treatment, vaccination may be less effective. The use of live vaccines should be avoided. Myositis with lovastatin and simvastatin, gingival hyperplasia with nifedipine, and convulsions with high-dose methylprednisolone can be occurred.

8.2 MMF (Mycophenolate Mofetil) or Myfortic® (enteric-coated mycophenolate sodium) drug interactions

To date, the following drugs have been known to interact with MMF (Mycophenolate Mofetil) or Myfortic® (enteric-coated mycophenolate sodium).

Drugs that are known to secrete into the renal tubules or alter the flora in the gastrointestinal tract, such as azathioprine (an immunosuppressive drug not permitted by the protocol), tacrolimus, acyclovir, antacids containing magnesium and aluminum hydroxide, cholestyramine, and probenecid may cause changes in enterohepatic recirculation and interact with MMF (Mycophenolate Mofetil) or Myfortic® (enteric-coated mycophenolate sodium). MMF is known to reduce exposure when used concomitantly with gastric acid secretion inhibitors. In general, the precautions specified in the product label for Mycophenolate Mofetil (MMF) or enteric-coated mycophenolate sodium (Myfortic®) should be followed.

1. Discontinuation of Clinical Study Drug

Subjects may voluntarily discontinue clinical research at any time and for any reason through the consent withdrawal process, and may stop taking study medications.

If a reason for discontinuing the study drug, Thymoglobulin®, occurs during the clinical study process stipulated in the study protocol, the last date of administration of the study drug, Thymoglobulin®, should be recorded in the case record program.

The researcher may terminate the subject's research process early for the following reasons.

1. If the researcher judges that the research drug will cause a significant risk to the safety of the subjects

- Signs of severe hypoimmune suppression (Banff G3 or higher, steroid resistant acute rejection, 2 or more acute rejection)
- Severe hyperimmune suppression
- malignant disease
- pregnancy

1. Loss of graft (even cases excluded due to possibility of loss are considered graft loss)
2. Death
3. Failure to follow-up (including all cases where the study was discontinued after receiving at least one dose of study drug after randomization)
4. If it is impossible to maintain the immunosuppressive therapy specified in the study protocol, the subject will drop out of the study and switch to the standard treatment of the research institution
5. Administrative issues unrelated to other validation failures, withdrawal of consent, disregard for testing procedures
6. Violation of the agreement

All subjects who are prematurely discontinued from the study process should, whenever possible, be visited for safety follow-up for monitoring of rejection, transplanted organ and patient survival rates, and information on immunosuppressive drugs. If the subject dies, does not visit the hospital for the scheduled visit, withdraws consent to the study, or does not continue the study due to failure of follow-up, the clinical study is considered to have been discontinued.

In the case of a patient who does not visit or the follow-up is interrupted, the “duty of care” is fulfilled by recording the steps taken to contact the subject in supporting documents such as the date of attempting a phone call or sending a registered mail.

Subjects may discontinue the study drug Thymoglobulin® if the investigator determines that continued administration of the study drug, Thymoglobulin®, will pose a significant risk to the safety of the subject. Discontinuation of study drug may be considered for the following reasons:

1) Signs of severe hypoimmune suppression

Banff classification Grade III or higher biopsy-confirmed acute rejection, steroid-resistant acute rejection, two or more (including twice) acute rejection regardless of transplant kidney biopsy findings

2) Signs of severe excess immunosuppression

Severe systemic infections occur (bacteria, fungi, viruses, protozoa, or unknown organisms)

BK polyoma infection confirmed by tissue biopsy or urinalysis.

3) Malignancy

4) Pregnancy

In addition to Thymoglobulin ® discontinuation requirements, if the investigator determines that continuing study drug would be detrimental to the patient's well-being, considering the benefits and risks, the investigator should discontinue Thymoglobulin ® in the relevant patients.

All subjects who discontinue the clinical study before the end of the clinical study (12 months after renal transplantation) may be switched to other immunosuppressive drugs according to the standard treatment practice of the study institution. Follow-up data should be collected up to 12 months after transplantation, if possible. Discontinuation of clinical drug administration should be recorded in the case record program, along with the date of last administration and the main reason for discontinuation.

1. End of clinical trial

Unless the clinical study is terminated early, the subject ends the clinical study at 12 months after kidney transplantation. The research results of subjects who have completed the study period are collected and recorded in the case record program, and data related to all studies can be utilized by the relevant research institute. The subject's personal information (name, resident registration number, address, phone number, etc.) must be kept confidential. Subjects may voluntarily withdraw from the clinical study at any point, and the researcher may remove the patients from the enrollment if they have reasons for dropping out of the study or discontinuing the study drug. Subjects who have completed the clinical study after 12 months after the kidney transplant follow the policy of the relevant research institute.

If the study is prematurely discontinued for any reason prior to the end of the study, the subject will be scheduled for a visit as soon as possible so that all assessments listed on the final visit can be completed at this time.

1. Visit schedule and evaluation, immunosuppressive regimens by visit schedule

For schedules for visits and evaluations, see “Visit Schedule and Evaluation” bleow. The “V” sign indicates the practice on visitation. Subjects must visit the hospital on the scheduled date. The “visit window” for visits 1, 2, and 3 is 7 days (visit days ± 7 days), and the “visit window” for visit 4 is 14 days. days (visit days ± 14 days).

| **Visit Schedule and Evaluation** | | | | | |  |  |
| --- | --- | --- | --- | --- | --- | --- | --- |
| **Lists** | **Screen/**  **Baseline** | **Visit 1 (D14)**  **±7** | **Visit 2 (M1)**  **±7** | **Visit 3 (M3)**  **±14** | **Visit 4 (M6)**  **±14** | **Visit 5 (M9)**  **±30** | **Visit6**  **(M12)**  **±30** |
| Informed consent form | V |  |  |  |  |  |  |
| Selection/exclusion criteria | V |  |  |  |  |  |  |
| Recipient background information | V |  |  |  |  |  |  |
| Donor background information | V |  |  |  |  |  |  |
| Transplant information | V |  |  |  |  |  |  |
| Past history information | V |  |  |  |  |  |  |
| Vital signs | V | V | V | V | V | V | V |
| Physical examination | V | V | V | V | V | V | V |
| Concomitant medication | V | V | V | V | V | V | V |
| Viral tests | V |  |  |  |  |  |  |
| Pregnancy test^[[1]](#footnote-1)^ | V |  |  |  |  |  |  |
| Hematology test^[[2]](#footnote-2)^ | V | V | V | V | V | V | V |
| Blood chemistry test^[[3]](#footnote-3)^ | V | V | V | V | V | V | V |
| Lipid test (Cholesterol, TG, HDL, LDL) | V | V | V | V | V | V | V |
| Urine test | V | V | V | V | V | V | V |
| Glycated hemoglobin (HbA1C) | V |  |  | V |  |  | V |
| CNI dosing record |  | V | V | V | V | V | V |
| Steroid dosing record |  | V | V | V | V | V | V |
| MMF or Myfortic® dosing record |  | V | V | V | V | V | V |
| Thymoglobulin® dosing record |  | V |  |  |  |  |  |
| Other immunosuppressant dosing record |  | V | V | V | V | V | V |
| Oral hypoglycemic agents or insulin dosing record | V | V | V | V | V | V | V |
| CNI baseline blood level |  | V | V | V | V | V | V |
| Record of renal biopsy (if applicable) |  | V | V | V | V | V | V |
| (Serious) Adverse events (if applicable) |  | V | V | V | V | V | V |
| Infection (if applicable) |  | *V* | *V* | *V* | V | V | V |
| Dialysis after transplant (if applicable) |  | V | V | V | V | V | V |
| Graft loss (if applicable) |  | V | V | V | V | V | V |
| Malignancy (if applicable) |  | V | V | V | V | V | V |
| Subject mortality (if applicable) |  | V | V | V | V | V | V |
| Violation of the agreement (if applicable) |  | V | V | V | V | V | V |
| End of treatment and clinical study |  |  |  |  |  |  | V |

1. Exposure to Study Drugs and Immunosuppressant Drugs and Medication Compliance

All immunosuppressive medications (Tacrolimus/cyclosporine, steroid, MMF (Mycophenolate Mofetil) or Myfortic® (Enteric-Coated Mycophenolate Sodium), Thymoglobulin ®, etc.) taken during the clinical study are recorded in the case record program (start date, medication dose, date of discontinuation, reason for dosing or change).

Other medications that were started prior to the start of study treatment and continued until the start of the study, or started during study treatment, are recorded in the case record program. (start date, medication dose, date of discontinuation, reason for dosing or change).

At each visit, the investigator or study staff should evaluate medication adherence.

1. Efficacy Assessment

In order to evaluate the efficacy of the study drug, the following items are analyzed.

- Incidence of BPAR (Biopsy Proven Acute rejection) up to 12 months after kidney transplantation
- Frequency of overall rejection up to 12 months after renal transplantation
- Steroid free (protocol success) rate at 12 months after kidney transplant
- Calculated glomerular filtration rate at 12 months after renal transplantation
- Patient and graft survival rates up to 12 months after renal transplantation.
- Urine proteinuria at 2 weeks 1, 3, 6, and 12 months after renal transplantation
- Frequency of DGF after kidney transplantation

Transplant kidney function

The details of the variables collected for the following effectiveness analysis are as follows:

a. Serum creatinine: For serum creatinine analysis, venous blood is drawn and analyzed at the institution's regional laboratory.

b. Glomerular filtration rate (GFR): The glomerular filtration rate (GFR) is the clinically most accurate indicator of transplant kidney function and correlates with the clinical severity of renal dysfunction. In this study, the GFR value calculated according to the Modification of Diet in Renal Disease (MDRD) method is used as the primary outcome variable.

Rejection

All presumptive rejections were recorded on the date when rejection was first suspected, whether a biopsy was performed, whether a presumptive investigation was performed (also record that date), whether anti-rejection treatment was administered, whether acute rejection was confirmed, or a final clinical diagnosis was established, and the final clinical results are recorded in the case record program.

The researcher also records suspected cases of acute rejection in the case record program along with treatment details. If acute rejection is suspected, a biopsy of the graft should be performed, and treatment may begin even before biopsy results are confirmed. Biopsy results are read by an in-house pathologist according to the Banff 07 classification and recorded in the biopsy section of the case record program. When analyzing efficacy and safety, acute rejection *Grade I or higher* (including Grade I), which are histologically confirmed and treatment is undertaken, are considered as acute rejections.

Rejection is an event that is considered as the primary efficacy variable and is not recorded as an adverse event. If an acute rejection is suspected but is found to be due to another disease, it is recorded as an adverse event (AE). Whenever an acute rejection reaction that is presumed clinically and treated or confirmed through biopsy occurs, events are recorded. A rejection that occurs more than 14 days after the occurrence of a single rejection is considered a new rejection.

Kidney biopsy

Kidney biopsy can be performed at any time upon the decision of the researcher according to the clinical progress, suspicion of rejection, proteinuria, or other changes in the transplanted kidney.

In all presumed rejection, a graft biopsy is performed before initiation of anti-rejection therapy or at least 48 hours after initiation of therapy. The biopsy is read and interpreted by a pathologist at the research institution. Results are recorded and used for efficacy analysis. Acute rejection confirmed by biopsy is classified as Banff classification grade IA, IB, IIA, IIB, or III. If a kidney biopsy is performed, all findings other than acute rejection should be recorded.

Clinical rejection

If rejection is suspected in the clinical course but histological diagnosis cannot be made, it can be diagnosed as clinical rejection and treatment can be started.

Graft loss or death

Graft loss occurs when: 1) the subject started dialysis and was unable to discontinue dialysis thereafter, 2) transplant graftectomy was performed, and 3) when appropriate imaging techniques confirmed irreversible cessation of blood flow to the graft. If the subject underwent transplant graftectomy, the date of graftectomy is the date of graft loss. In addition, if it is confirmed by imaging diagnosis, it is the date of imaging diagnosis, and if the above three items are duplicated, the earliest date is selected as the date of graft loss. The reason for graft loss is recorded in the case record program. Graft loss is considered a serious adverse event, and should be recorded in the case record program and a serious adverse event report should be prepared and faxed to the principal investigator within 24 hours. If the transplanted kidney is functioning and the patient dies, the date of death is considered graft loss from which the graft survival is calculated.

1. Safety Assessment

Safety Assessment

Infection

When infection occurs, the identification of bacteria, culture, antibiotics start date, extinction date, maximum intensity, reporting method, progress, severity, outcome, causal relationship, predictability, measures taken, adjustment of immunosuppressive drugs, etc. shall be recorded.

CMV infection can be confirmed when seroconversion occurs in negative patients or when CMV is isolated from urine, saliva, blood, or other tissues. If there is evidence of CMV viremia, treatment can be determined based on a complex judgment. The timing and method of treatment depend on the researcher's judgment, and the research institution's standard treatment is applied.

BK infection is diagnosed when decoy cells are identified in urine cytology, quantitative or qualitative PCR (polymerase chain reaction) positive in urine or serum, and BKVN can be confirmed if confirmed histologically in transplant biopsy. If there is no clinical etiology and there is an increase in serum creatinine, it can be suspected, and treatment can be determined based on a complex judgment. The timing and method of treatment depend on the researcher's judgment, and the research institution's standard treatment is applied. If some changes in immunosuppressive drugs are necessary, they can be changed while still enrolling in the study (leflunomide, etc.)

When a drug is used to treat an infection, the name of the drug, the date of start of administration, the date of discontinuation of administration, dose, route of administration, and indications shall be recorded in the case record program.

Frequency of hematological side effects (Anemia, Leukopenia, thrombocytopenia)

Incidence of tumors

Frequency of AE/SAE occurrence

Adverse reaction

An adverse reaction is any undesirable sign, symptom, or disease that appears or worsens after the initiation of study drug administration, including cases where the reaction is not considered related to the study drug. Study drug includes the actual study drug, reference drug or placebo that are to be evaluated administered at all stages of a clinical study. An adverse event is considered only if the medical condition/disease present before the initiation of the study drug worsens after the initiation of the study drug. Abnormalities in laboratory values ​​or laboratory results are considered adverse events only if they cause clinical signs or symptoms and are clinically significant or require treatment.

The occurrence of an adverse reaction can be confirmed by interviewing, examining, and testing the subject at each visit by the researcher during the study period. If all adverse reactions occur, adverse reaction name, onset date, extinction date, maximum intensity (mild, moderate, severe), reporting method (question, measurement, observation, spontaneous report), course (continuous, intermittent, once), severity (not serious, serious), outcome (no sequelae of recovery, with sequelae of recovery, presence of side effects, no progression, presence of side effects, death, no follow-up required), causation (certain, fairly certain, possible, unlikely, difficult to evaluate, not evaluable), predictability (predicted side effects, unexpected side effects), actions taken (no action taken, concomitant administration of therapeutic drugs, non-drug treatment, hospitalization/extension of hospitalization period), actions taken of study drug (no action taken, dose adjustment, temporary discontinuation, permanent discontinuation) should be evaluated and recorded in the case record program.

If an adverse reaction occurs, it should be followed until resolved or considered permanent. Whether there is a change in severity, the presumed relationship with the study drug, the intervention required for treatment, and the result of the treatment are evaluated at each visit (more frequently if necessary). The study drug is a drug that is currently on the market, and information on common side effects that are already known is enlisted in the product description. This information is also written in the subject consent form, and if necessary, is discussed with the subject during the clinical research period.

- A serious adverse event (SAE) is any unintended medical event that occurs at any dose and results in:
- In this study, a serious adverse event (SAE) is defined as:
  - Fatal or life-threatening
  - If it results in persistent or significant disability/incapacity
  - If congenital defects/fetal malformations are concerned
  - If hospitalization is required or an extension of the hospitalization period is required (except for the cases below)
    - - Routine treatment or monitoring not related to deterioration
      - Elective or preplanned treatment of pre-existing conditions that are not related to the study indication and that have not worsened since the start of study drug administration
      - Emergency outpatient treatment that does not meet the definition of a serious adverse event mentioned above and does not require hospitalization
      - Temporary hospitalization for social reasons or in circumstances where the subject's general condition has not deteriorated
  - A medically emergency condition, i.e. the subject is in danger or requires medical or surgical intervention to prevent any of the above
  - Graft loss

Unlike regular safety evaluation, serious adverse events (SAEs) are continuously monitored and a separate reporting procedure is followed. In the case of a serious adverse reaction, the research institute must record it in writing on the serious adverse reaction report form and report it to the principal researcher within 24 hours, and the principal researcher should then report to other researchers.

**1.2 Obligation of Researchers for Safety Reporting**

**1.2.1 Adverse Events (AE)**

- The safety observation period for all adverse events (including serious adverse events) begins from the time the patient signs the subject consent form.
- All adverse reactions, regardless of severity or relevance to the study drug, that occurred during the administration period from the date the subject signed the consent form (i.e., even if it occurred during the screening period without study drug administration) and up to 30 days after the last study drug administration should be recorded on the relevant page of the case report.
- Where possible, it should be reported as a single syndrome or diagnosis rather than a symptom. The investigator should specify the date of onset, severity, measures related to the study drug, corrective treatment/therapy performed, additional tests performed, and the investigator's opinion on whether there is a reasonable possibility that the adverse reaction was caused by the study drug.
- Additional tests performed due to adverse reactions or related additional procedures or tests not specified in the protocol but performed to support safety evaluation should be recorded in the case report.
- All laboratory tests performed in the study flow chart are reported on the relevant page of the case report regardless of the above adverse reaction criteria (hematology, biochemistry, etc.).
- Record abnormal laboratory results, vital signs, or ECG as adverse events only if:
- symptoms present (and) / (or)
- Requires corrective treatment or consultation (and) / (or)
- Satisfies materiality criteria (and) / (or)
- In case of need to discontinue study drug administration/dosage change or postponement

**1.2.2 Serious Adverse Events (SAEs)**

If a serious adverse reaction occurs, the researcher should immediately do the following.

*Note: Report serious adverse reactions to the IRB within 24 hours of the investigator's awareness of them.*

ENTER: Enter information related to serious adverse events on the corresponding page of the case report.

All subsequent updated data must be appropriately recorded in the case report, and additional documents and information (laboratory data, concomitant medications, subject's condition, etc.) should be recorded in a 'follow-up report' within one working day and reported to the IRB. Every effort should be made to ensure that follow-up is conducted within 7 days of initial notification in the case of serious adverse reactions that are death or life-threatening, and additional records should be made accordingly.

**1.2.3 Follow-up**

- The investigator must take all appropriate measures to ensure the patient's safety and follow-up the results until the patient's condition is normalized.
- In the case of a patient whose treatment was prematurely discontinued, observation of the patient is continued until the trial end date defined in the clinical trial protocol.
- For serious adverse reactions, patients should be followed until clinical recovery is complete and until laboratory results return to normal or progression has stabilized.

**1.2.4 Guidelines for Pregnancy Reporting**

Pregnancy is subject to rapid reporting, which must be reported immediately in all cases. When pregnancy occurs, administration of clinical trial drugs must be discontinued and reported to the person in charge of safety information management and the principal researcher within 24 hours from the date of recognition by the researcher according to the adverse reaction/expedited report form. Follow-up is required until the results of the pregnancy are confirmed. The follow-up information includes spontaneous or artificial abortion, details of childbirth, presence or absence of congenital abnormalities, birth abnormalities, or maternal or neonatal complications. If a subject experiences a serious adverse reaction during pregnancy, the researcher in charge must report it using the 'Serious Adverse Event Report Form' and should include an assessment of possible relation between the test drug and the event.

**1.2.5 Guidelines for reporting overdose**

An overdose of study drug, whether intentional or accidental, should be recorded as an ‘adverse event’. In the case of an overdose accompanied by symptoms, it must be reported to the person in charge of safety information management within 24 hours from the date of recognition by the researcher according to the adverse reaction/rapid report form, even if it does not meet the criteria of seriousness. It is commonly referred to as overdose when it exceeds 30% of the dose that should be administered per body surface area in each cycle.

Tumor

All types of malignant tumors (including skin tumors) during the clinical study period are recorded in the Adverse Events (AE) section of the case record program, and are considered serious adverse events (SAEs) and must be recorded in writing on the Serious Adverse Event Reporting Form. Report to the lead researcher within 24 hours, and the lead researcher must report to other researchers. If a subject develops a malignancy after premature discontinuation of clinical study treatment, it should also be recorded in the case report.

Physical examination

A full physical examination is performed during the screening/baseline period for 6 times during the study period. Height is recorded in the screening/baseline only case report program. This includes a semi-quantitative assessment of study drug-related adverse events (skin changes, edema, alopecia, muscle and skeletal pain, blood pressure, and some selected laboratory parameters, etc.).

Information on the physical examination should be recorded and stored in the supporting documents of the research institute. Significant matters that appeared prior to the start of the clinical study should be recorded in the case record program. Materials that conform to the definition of an adverse reaction, and significant matters that occur after administration of clinical drugs are recorded in the case record.

Vital signs

Blood pressure and pulse were measured on the same arm for each measurement, and were measured after the subject had rested in a sitting state for at least 5 minutes. Body weight is recorded in kilogram. The results are recorded in the case record.

Laboratory tests

During the research period, the researcher may conduct various tests according to the standard treatment guidelines of the research institution. The test is performed to confirm the safety and changes of the patient and the transplanted kidney according to the researcher's judgment, and can be performed to confirm suspected diseases or adverse reactions. If an abnormal test result is confirmed, the researcher should record it in writing as the adverse event.

Pregnancy test (urine or serum) is performed at the time of screening/baseline (the result must be confirmed before taking the study drug), and only subjects with negative pregnancy test results can be enrolled in the study. Registered subjects must follow the method of contraception defined in the clinical research protocol during the period of participation in clinical research. If the subject's pregnancy is suspected, it can be confirmed through a urine or serum test. If a subject is confirmed to be pregnant during the research period, the researcher who recognizes the fact must record it on the serious adverse reaction report form and report it to the principal researcher within 24 hours, and the principal researcher reports to other researchers. Pregnancy cases are followed up to determine the outcome, such as natural or artificial abortion, details of birth, and the presence or absence of birth defects or congenital abnormalities, and are recorded on a separate written case record. All female subjects who may become pregnant after initiating clinical study treatment will be discontinued from the study and switched to the local standard of care.

During the study period, clinical laboratory and diagnostic data will be analyzed by the institution's regional laboratory as specified below.

Inspect the following variables by referring to the visit schedule and evaluation items for each inspection period.

- Hematology: red blood cells, hemoglobin, hematocrit, platelets, white blood cells, and percentage of differential counts.
- Blood Chemistry: sodium, potassium, calcium, magnesium, phosphoric acid, urea (BUN), creatinine, blood sugar, uric acid, AST, ALT, alkaline phosphatase, albumin, bilirubin, cholesterol, HLD, LDL, triglyceride
- Serum virus test: hepatitis B (HbsAg), hepatitis C, HIV, CMV.
- Urinalysis: standard urine analysis and single urine PCR (Protein/Creatinine Ratio)
- Pregnancy test: urine or serum B-hCG (women of childbearing age)

All subjects are tested for hepatitis B (HBsAg), hepatitis C, HIV, and CMV before enrolling in the study. Subject registration can proceed based on the results of tests conducted within 12 months prior to subject registration, except for cases where clinical variation is suspected. Subject with hepatitis B surface antigen present or HIV-positive subjects are excluded from the study.

Tolerability

Tolerability of each regimen is assessed by the percentage of subjects who needed reduction or discontinuation of study drug due to adverse events.

1. ***Data Management***

## *15.1 Research Institute Monitoring (Monitoring)*

There is no separate monitoring agent in this study. However, researchers regularly check the completeness of subject records, the accuracy of case record programs, clinical research protocols and registration progress, and review whether the research process is being implemented according to regulations.

Investigators maintain supporting documents for each subject participating in clinical research, including demographic and medical information, laboratory data, and other test and evaluation results. All information included in the case record program should be traceable to the source documents of the subject file. The researcher keeps the original consent form signed by the subject, and provides a copy of the signed consent form to the subject.

In addition, standard verification of the existence of informed consent from subjects, compliance with selection/exclusion criteria, records of serious adverse events, and data records to be used as all efficacy endpoints and safety endpoints is conducted through internal inspection of the institute. Additional checks regarding consistency of source data and case-record programs may be performed. No information about the subject's identity in the source document should be exposed to the outside.

Collected data is stored in a locked device and stored on a computer with restricted access. In addition, access to the case report is restricted with a password, and access is limited to the research director and co-researchers.

## *15.2 Case record and database management*

15.2 Case record and database management

Participating researchers review the completeness and accuracy of the case record program prepared by *the person in charge of the research institution*, and make necessary corrections and data additions. Obvious errors are corrected by the data manager, and a data clarification form (DCF) is issued to the research institute for data requiring confirmation. The person in charge of the research institute corrects the data and sends it to the person in charge of the data.

Data is frozen when the data management quality control procedure is completed.

In order to protect the identity of research subjects, interviews, explanations, and consent are obtained in an independent space, and identification information of research subjects is anonymized. All collected data are stored in a locked device, stored on a computer with restricted access, with restricted access to case reports with passwords and individuals with permitted access (research director, co-researcher).

1. Data and statistical analysis

All statistical analyzes are performed by PI. The final analysis is performed within 6 months of the time denoted by the end of the trial date of the last subject.

Analysis target group

All analysis groups analyze both the Full analysis set (FAS) and PP (per protocol) groups.

The Full analysis set (FAS) group is used for the analysis of the primary variables. This group is composed of all subjects who received the study drug after writing the consent form for efficacy and stability analysis. It was not associated with protocol violations, adherence to study drug, or early discontinuation of the trial. Patients who were prematurely terminated for reasons such as withdrawal of consent prior to study drug administration are excluded and analyzed.

 For an auxiliary purpose, analysis is conducted on the Per-protocol (PP) group to investigate the impact of missing data and protocol violators. Even in this method, patients who were prematurely terminated for reasons such as withdrawal of consent excluded from the FAS group are excluded from the analysis. If the difference between the PP and FAS groups is less than 10% of the patients in the FAS group, the analysis is not performed.

 Stability analysis consists of All patients as treated (APaT) group. In this group, the group that did not receive the study drug was also excluded.

Adherence to treatment

For concomitant immunosuppressive drugs, other concomitant drugs, and therapeutic drugs, the average daily dose by type is summarized by visit to monitor the adherence of the patients to drugs.

**Test drug**

The test drug is Thymoglobuline®, and prescribed drugs other than immunosuppressants are considered concomitant drugs.

Summarize the total administered dose (mg/kg) of Thymoglobuline® test drug. (Weight is based on dry weight at the time of screening). Summarize the frequency and average dose of dose reduction (including temporary discontinuation) according to the protocol (PP) guidelines. Reasons for dose adjustment (including temporary discontinuation of administration) are summarized by frequency distribution. Analyze permanent treatment discontinuation by frequency. This analysis is performed on the intention-to-treat (ITT) population.

**Concomitant immunosuppressive drugs**

All immunosuppressive drugs administered to subjects should be summarized by treatment group and study period, and organized in a frequency distribution table. All immunosuppressive drugs are summarized individually: Tacrolimus/Cyclosporin (CNI), Steroids, Antimetabolite (MMF or Myfortic®), and other immunosuppressive drugs.

Compliance is defined when subjects are administered between 80% and 120% of the prescribed dose.

**Concomitant drug**

All analysis results of concomitant drugs should be organized by period.

**Efficacy evaluation**

The primary purpose of the efficacy evaluation analysis is to compare and verify the following items

- Incidence of Biopsy-Confirmed Acute Rejection (BCAR) up to 12 months after renal transplantation
- Incidence of overall rejection by 12 months after renal transplantation
- Incidence of efficacy failure (steroid re-usage, graft loss rate, death and loss of follow-up) up to 12 months after renal transplantation
- Survival of subjects and transplanted kidneys up to 12 months after renal transplantation
- Predicted calculated glomerular filtration rate at 12 months after kidney transplantation: Modification of Diet in Renal Disease (MDRD)

**Safety evaluation**For the primary purpose of safety evaluation analysis, the following items are compared and verified

- Frequency of anemia, leukopenia, and thrombocytopenia at each period after kidney transplantation
- Compare the frequency of newly developed diabetes and glucose metabolism disorder at 1 month and 12 months after renal transplantation with immunosuppressive drugs
- Frequency and severity of infections requiring drug treatment
- Expression frequency of BK virus and CMV virus up to 12 months after kidney transplantation
- Cardiovascular events (Acute Myocardial Infarct, Unstable Angina Pectoris, Heart Failure, Stroke).
- Frequency and severity of adverse events and serious adverse events
- Frequency of adverse events leading to study drug discontinuation

1. **References**

1. Gurk-Turner C. et al. Transplantation 2008;85: 1425–1430

2. Hrick DE et al. American Journal of Transplantation 2002; 2: 19-24

3. Martin ST et al. Pharmacotherapy 2011; 31(6): 566-573

4. Klem P et al. (Transplantation 2009;88: 891–896)

5.Kramer BK et al. American Journal of Transplantation 2010; 10: 2632–2643

6. Veenstra DL et al. Am J Kidney Dis. 1999 May;33(5):829-39.

7. Pascual J etal.Cochrane Database of Systematic Reviews 2009, Issue 1

8. Birkeland SA. [Transplantation.](http://www.ncbi.nlm.nih.gov/pubmed/9825819)1998 Nov 15;66(9):1207-10

9. Birkeland SA. Transplantation. 2001 Apr 27;71(8):1089-90.

10. Matas AJ. et al. Am J Transplant. 2005 Oct;5(10):2473-8.

11. Woodle ES. Et al. Ann Surg2008;248: 564–577)

12. Woodle ES. Et al. Clin Transplant 2010: 24: 73–83

13.Hardinger KL et al. J Transplantation 2010: 1-8

14. Stratta RJ et al. Transplantation Proceedings 2005: 37, 3546–3548

15. Brennan DC et al. N Engl J Med 2006;355:1967-77.

17. Meijer E. et al. Biol Blood Marrow Transplant. 2009 Nov;15(11):1422-30

18. Hamadani M et al. Biol Blood Marrow Transplant. 2009 Nov;15(11):1422-30

**Appendix 2. Responsibilities and Authority for Clinical Research**

**Compliance with relevant regulations and ethical principles**

This clinical study complies with the ethical principles described in the institution’s regulations and the Declaration of Helsinki, and all data related to the study, such as plans and budgets, are conducted under the review and approval of the institutional review board (IRB). When matters related to the safety of subjects occur, all information is collected through the CRO (Clinical Research Organization) and delivered to each research institution so that the research director can decide whether or not to proceed with the research.

**Investigator and institutional review board (IRB) responsibility**

Prior to the commencement of a clinical study, the clinical study protocol and the proposed consent forms are submitted to an appropriately constituted institutional review board (IRB) for review and approval. If the clinical research protocol and consent form are approved, the institutional review board (IRB) approval statement with the signature and date recorded is checked prior to the commencement of clinical research. Prior to the start of the clinical study, the researchers sign the signature page of the protocol, and are entitled to conduct the clinical study according to all instructions and procedures described in these documents and the protocol, and give consent to monitoring agents and related authorities to have access to all relevant information and records.

**Subject consent**

Only patients who meet the selection criteria (if required by law or regulation, in the presence of a third party) may sign and submit a consent form approved by the institutional review board (IRB), or they may give their consent in person. One can register and participate in clinical research after receiving the patient's entrustment or obtaining the consent of a legally valid representative. Prior to the implementation of all clinical research-related procedures (all procedures described in the research protocol), the subject consent procedure should be preceded.

A proposed informed consent form that complies with relevant regulations and is considered appropriate for this clinical study is attached in the appendix. All changes to the consent form proposed by the researcher should be followed by obtaining the consent of the research sponsor before submission to the institutional review board (IRB), and after obtaining approval from the institutional review board (IRB), a copy of the approved consent form is submitted to monitoring agents.

**Change of clinical research protocol**

All changes and additions to the protocol are made in the form of a written change protocol approved by the research sponsor, health authorities (if necessary), and the institutional review board (IRB). If changes are related to subject safety, they may be implemented prior to institutional review board (IRB) approval. Even in the case of a violation of the protocol of this clinical study, measures can be taken immediately (prior to institutional review board (IRB) approval) with considering the safety of all participating subjects as the highest priority and if this occurs, the institutional review board (IRB) shall be notified within 10 working days.

**Discontinuation of clinical study**

Investigators and research sponsors retain the right to discontinue this clinical research under circumstances specified in the clinical research contract. When the study is discontinued, patients registered as subjects are treated with the standard treatment of each research institute, and the research results up to the point of discontinuation can be collected and analyzed.

**Publication and presentation of results**

Clinical research sponsors can review all planned presentations (communications, presentations, broadcasts, etc.) and published manuscripts (manuscripts) based on research findings. However, clinical research sponsors cannot restrict or hinder the publication or other dissemination of clinical research results by researchers, and research sponsors support the publication and publication of research results.

**Document storage**

Data stored in documents and media related to clinical research are preserved at the research institute for at least 3 years after the completion of the research.

**Appendix 3. Compensation Protocol**

**Purpose**

In accordance with Article 31 of the Enforcement Rules of the Pharmaceutical Affairs Act, the person in charge of clinical research is responsible for compensating for physical damage and all other damages occurred in the subjects.

**Compensation principle**

Clinical research sponsors, research directors, and research staff must do their best to faithfully comply with the relevant laws and regulations and the contents of the mutually agreed clinical research protocol so that subjects do not suffer any disadvantages related to clinical research. Despite these efforts, the subjects suffered from harmful and unintended reactions that are judged to have a causal relationship between clinical research methods or drugs during clinical research (including cases where it is not proven that they are not related to clinical research drugs) that resulted in the following events should be paid with sufficient compensation for treatment costs that are not paid by insurance, government programs, or other third parties through clinical research compensation insurance for overall clinical research in accordance with the compensation evaluation criteria.

(1) Extension of hospitalization is required; or

(2) Significant impairment or impairment of function; or

(3) Congenital malformations or abnormalities; or

(4) Death or endangered life

**Compensation requirements**

The person in charge of clinical research is responsible for compensating all physical damage to the subjects during the clinical research period if all of the following requirements are met.

(1) In the case of physical damage caused by ATG prescribed during the course of the research according to the agreed research protocol;

(2) When the subject complied with all contents of the clinical research protocol;

(3) If it was not attributable to the subject's apparent negligence or negligence;

(4) When the subject complied with all instructions of the principal researcher or the person in charge of the research;

(5) If the subject took necessary measures to minimize the occurrence of damages due to damage to the body

**Scope of compensation and reasons for exclusion**

Notwithstanding the previous compensation requirements, each of the following cases is excluded from the scope of compensation according to this compensation agreement.

(1) In the course of clinical research, damage due to insufficiency of expected effect or efficacy of pharmaceuticals (including cases due to the progression and aggravation of a subject's anesthesia);

or

(2) Damage caused by the subject's negligence

In addition, the subject is not eligible for compensation in the following cases.

(1) Failure to comply with the clinical research protocol or violating the researcher's instructions, or damage caused by side effects caused by clinical research drugs not provided by the researcher; or

(2) Damage due to common complications occurring during the course of treatment of the disease; or

(3) Compensation for not showing valid results for clinical research drug indications; or

(4) Damage caused by deviation from the agreed contents, such as mutually agreed clinical research protocols or contracts, explanatory statements, and written consent; or

(5) Damage caused by negligence of the subject or guardian

**Compensation Evaluation Criteria**

(1) If there is a compensation amount or action agreed between the parties in advance for an expected adverse reaction, it will be compensated accordingly

(2) In other cases, the nature, extent, duration, continuity, and similar cases of bodily damage are comprehensively considered and compensated according to an appropriate amount.

(3) If the agreement in the preceding paragraph has not been reached between the parties, the parties shall seek advice from experts acceptable to them to resolve the matter, and in this case also, if an agreement is not reached, the contents of the court's judgment shall be considered to determine the compensation accordingly.

**Compensation procedure**

(1) Subjects who have suffered bodily damage according to the studyl’s compensation policies must first request necessary medical measures from the research director or research institute of the clinical research.

(2) Subjects whose bodily damage has not been cured despite the measures of the research director or research institution may request compensation from the clinical research director.

(3) The clinical research director completes an investigation to determine whether or not the subject is eligible for compensation within 14 days after receiving the above compensation request, and notifies the subject of the details.

(4) If the subject has an objection to the above notification, he/she must notify the person in charge of the clinical research of the objection within 5 business days from the date of receipt of the above notification.

(5) If the subject does not present the objection even after receiving the notification in paragraph (3), it is understood that both parties have agreed on the compensation according to the above notification.

(6) If the subject notifies the objection pursuant to paragraph (4), the clinical research director recommends multiple objective experts to determine whether the subject is eligible for compensation, and the subject is notified of the above recommendation and can choose one expert within 3 business days (If the subject does not choose, the clinical trial director will make a random choice).

(7) If an agreement cannot be reached on the results of the consultation between the subject and the expert selected according to the provisions of paragraph (6), compensation will be given according to the final judgment of the court and the corresponding decision.

**Extent of coverage**

(1) This compensation agreement is generally applied to the subjects participating in all clinical research conducted by the clinical research director, within the scope of which there is no other agreement between the clinical research director and the subjects.

(2) All agreements entered into by the subject with other third parties related to the clinical research without obtaining approval from the clinical research director for compensation related to the clinical research are not effective with respect to the compensation rules related to this research.

By signing below, it is affirmed that the person in charge of the clinical research takes care that the patient does not suffer any disadvantages due to the clinical research by referring to the various contents mentioned above, and promises to take responsibility for the damage suffered by the research subject in accordance with the above if a problem occurs during clinical research trials.

Director of Clinical Research Signature

#

1. [↑](#footnote-ref-1)
2. 1 Pregnancy test: Urine or serum B-hCG (female of childbearing age) should be confirmed and confirmed prior to study drug administration.

   Hematology tests: red blood cells, hemoglobin, hematocrit, platelets, white blood cells and percentage of differential counts. [↑](#footnote-ref-2)
3. Blood chemistry tests: sodium, potassium, calcium, magnesium, phosphate, urea (BUN), creatinine, blood sugar, uric acid, AST, ALT, alkaline phosphatase, albumin, bilirubin, cholesterol, HLD, LDL, triglyceride [↑](#footnote-ref-3)
